# Electronic properties of poly (triazine imide) distance-controlled of the NH and BH substitutions and their impacts on the photocatalytic descriptors

**DOI:** 10.1007/s00894-026-07005-x

**Published:** 2026-10-20

**Authors:** Letícia C. S. Faria, Bárbara M. Brum, Letícia M. S. V. Queiroz, Josiane R. C. Silva, Francisco B. C. Machado, Leonardo T. Ueno, Luiz F. A. Ferrão

**Affiliations:** 1 Department of Chemistry, Aeronautics Institute of Technology, São José dos Campos, 12228-900 Brazil; 2 Advanced Scientific Computing and Modeling Laboratory, Aeronautics Institute of Technology, São José dos Campos, 12228-900 Brazil

**Keywords:** Poly(triazine imide), Photocatalytic water splitting, DFT calculations, Substitution distance, Charge transfer, Electrophilicity index

## Abstract

**Context:**

The development of efficient photocatalysts for water splitting using carbon-nitrogen materials, such as poly (heptazine imide) and poly (triazine imide), shows promise due to the stability, cost, and visible light absorption of these materials. However, their low light absorbance and high recombination (low charge transfer) pose key challenges that limit their application and require further development. One way to address these limitations is through dual functionalization, which is tailored to increase absorbance and charge transfer. In this context, we characterize dually modified poly(triazine imide) (PTI) systems in which one fragment contains a nitrogen site functionalized with a hydrogen atom (herein referred to as NH functionalization), while another fragment contains a nitrogen atom replaced by a BH group (herein referred to as BH substitution). In particular, we investigate how the spatial distance between these modified sites modulates the electronic properties of the PTI systems. The results reveal a clear exponential dependence of these properties on substitution distance, reflecting the short-range nature of electronic interactions in π-conjugated systems. Furthermore, a detailed thermodynamic analysis indicates that only structures with short substitution distances satisfy the rigorous potential requirements for driving both hydrogen and oxygen evolution reactions. These findings provide critical microscopic insights for the rational design and structural optimization of PTI-based materials for enhanced photocatalytic performance.

**Methods:**

Geometry optimizations and vibrational frequency analyses were performed using DFT with the ωB97X-D functional, which should effectively describe local and charge- transfer excitations in conjugated organic structures, combined with the def2-SV(P) basis set. Vertical excitations were calculated using TD-DFT with the same methodology. We evaluated ionization energy, charge-transfer character, electrophilicity (Δω^±^), and the ε^3^ stability index. Regression models were used to evaluate the relationship between substitution distance and the electronic properties of the studied structures.

**Supplementary Information:**

The online version contains supplementary material available at https://doi.org/10.1007/s00894-026-07005-x.

## Introduction

The global energy crisis and environmental pollution are among the most pressing challenges currently facing the world, driving nations to urgently seek sustainable and environmentally friendly energy solutions that combine cost-effectiveness with high efficiency to produce clean fuels, such as H_2_ [1]. In this context, photocatalysis emerges as a promising approach, as it directly harnesses solar energy to generate charge carriers capable of driving essential chemical transformations, including water oxidation and reduction. However, the development of materials that meet photocatalytic requirements while maintaining an attractive cost-benefit profile remains a significant challenge [2, 3].

The photocatalytic process is often described in terms of three fundamental steps: (1) photogeneration of charge carriers following light absorption, (2) migration of these carriers to the catalyst surface, and (3) initiation of redox reactions at the active sites. While the actual mechanism can be considerably more complex, involving recombination pathways, trapping states, and surface-mediated processes, this framework provides a useful conceptual basis for analyzing photocatalytic performance. For efficient operation, a semiconductor photocatalyst must absorb visible light and generate electron-hole pairs with sufficient lifetime and mobility [4–6]. Upon excitation, electrons are promoted to the conduction band (CB), leaving holes in the valence band (VB) [7, 8]. These photogenerated carriers may then reach the surface and participate in redox reactions associated with the oxygen evolution reaction (OER) and the hydrogen evolution reaction (HER). Therefore, appropriate alignment of the semiconductor band structure with the relevant energy levels of the redox species is essential to ensure both effective light harvesting and efficient charge transfer.

**Fig. 1:**
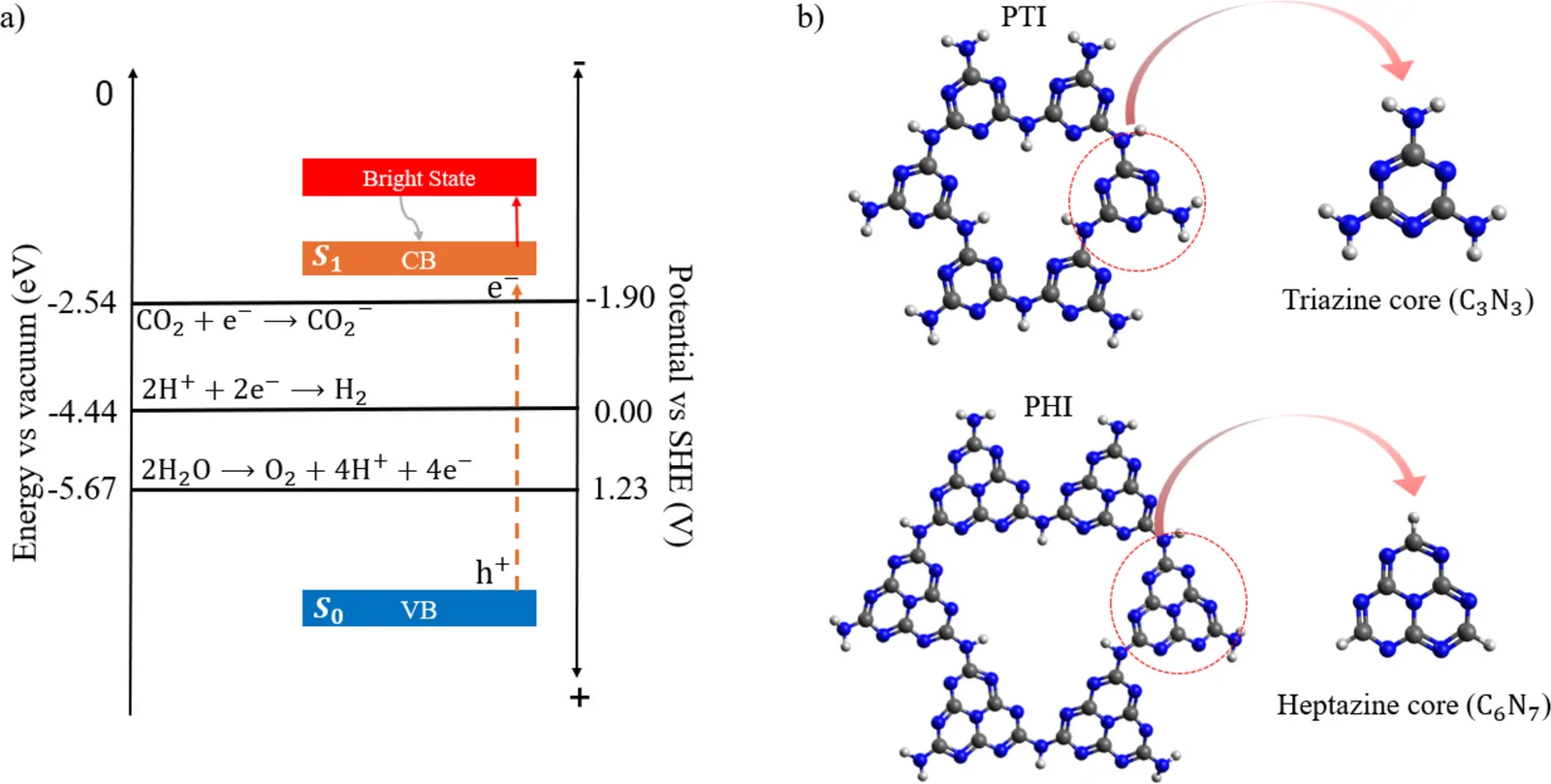
**a** Schematic diagram of the thermodynamic criteria referenced to the vacuum energy scale (eV) and to the standard hydrogen electrode (SHE). The thermodynamic potentials for the hydrogen evolution reaction (HER, 2H^+^ + 2e^-^ → H_2_) and the oxygen evolution reaction (OER, 2H_2_O → O_2_ + 4H^+^ + 4e^-^) are indicated at -4.44 eV (0.00 V vs SHE) and -5.67 eV (1.23 V vs SHE), respectively. The positions of the valence band (VB, S_0_) and conduction band (CB, S_1_) are shown together with the bright state, corresponding to the lowest excited state with significant oscillator strength. **b** Molecular structures of poly (triazine imide) (PTI) and poly (heptazine imide) (PHI). The highlighted regions indicate the fundamental structural units: the triazine core (C_3_N_3_) in PTI and the heptazine core (C_6_N_7_) in PHI

Besides solid catalysts, the present study considers the development and usage of molecular catalysts, in which the valence and conduction bands are translated into ground and excited states. The molecular energy levels are introduced into the scheme of Figure 1a. For a molecular catalyst, the position of the valence band (S_0_ energy level) was defined to be equal the ionization energy (in modulus). The conduction band level was represented by the first excited-state energy relative to vacuum, defined as the ionization energy minus the excitation energy (S_1_ energy level). The bright state, illustrated in red in Figure 1a, corresponds to the lowest electronic state with significant oscillator strength, indicating the energy levels at which the structure most effectively absorbs light. According to Kasha’s rule [9, 10], after excitation to the bright state, electronic relaxation to S_1_ occurs via internal conversion, if S_1_ is not a bright state, a process that typically occurs at a rate much faster than fluorescence [11].

Figure 1a presents the thermodynamic relations of the HER and OER reactions to molecular energy levels, with respect to the vacuum level, as well as to the standard hydrogen electrode (SHE) potential, based on the vacuum reference value of -4.44 eV.

The thermodynamic criteria for OER and HER are essential to define the requirements for water electrolysis. A minimum potential of 1.23 V (vs SHE) for OER at pH=0 and 25 °C establishes the thermodynamic limit [7, 12]. From a design perspective, it is desirable to identify structures that simultaneously satisfy the thermodynamic requirements for water oxidation and CO_2_ photoreduction. In the CO_2_ photoreduction mechanism, a key step is the activation of CO_2_ to form the ${CO}_{2}^{\bullet -}$ radical anion, which requires a potential of approximately 1.9 V [3, 13]. Structures whose energy levels fall within this range on the SHE scales, together with effective charge transfer characteristics, can donate an electron to CO_2_, inducing its bending from a linear to an angular geometry [14–17]. This structural distortion is a critical step in the activation process and plays a fundamental role in enabling subsequent reduction pathways.

Carbon-nitrogen materials have been widely recognized as influential organic polymeric semiconductors due to their diverse and outstanding properties. Among carbon nitrides, poly heptazine imide (PHI) [18–21] and poly triazine imide (PTI) [22] (Figure 1b) stand out because of their unique structures, which originate from distinct formation pathways of carbon nitride materials [23]. Both PHI and PTI retain the intrinsic characteristics of carbon nitride systems, such as suitable electronic structures, high thermal stability, and a graphite-like framework. PHI and PTI are nitrogen-rich polymers composed of cyclic heptazine and triazine units, respectively, interconnected through imine (-NH-) linkages. This structural arrangement provides both materials with extended π-conjugated systems at the molecular level, which play a key role in their optoelectronic and photocatalytic properties[24].

One of the main pathways described in the literature to synthesize PHI and PTI from melamine involves the initial conversion of melamine into Melam through calcination, followed by further condensation to form Melem. In the presence of molten salts during the subsequent steps, PTI can be obtained directly in a single process. Alternatively, Melem may evolve into PHI via the Melon intermediate and, upon prolonging the reaction time, it can ultimately be transformed into PTI. It has been established that the formation of PHI and PTI structures can be controlled by adjusting key parameters such as reaction temperature, atmosphere, type and proportion of molten salts, and reaction time [24–27]. In general, this synthetic route highlights the structural flexibility of carbon nitride materials and provides a convenient strategy to tune their crystalline, morphology, and electronic properties. These nanostructures, featuring a π-conjugated system, exhibit a band gap of approximately 2.7 eV in their polymeric conformers [28, 29]. Such a band gap enables visible-light absorption up to around 460 nm (2.7 eV); however, the absorption coefficient in this region remains relatively low, leading to limited light-harvesting efficiency [30].

Several polymeric structural modifications based on the PHI framework have been reported in the literature[31–35]. Functionalization such as hydrogenation (H) and borohydride substitution (BH) have demonstrated effectiveness in tuning the photocatalytic properties of these materials [36]. The substitution of BH groups into nitrogen sites in g-C_3_N_4_-based structures can facilitate electron charge transfer (CT) from the π-conjugated system to the empty 2p orbital of boron [37, 38]. Moreover, the electron-deficient character of boron enhances the migration of π-electrons, which is expected to improve the separation and transport of photogenerated charge carriers. Although dual functionalization with H and BH groups in heptazine-based structures has been shown to enhance photocatalytic properties, the influence of substitution topology in triazine-based structures, as well as the role of the interfacial distance between these active centers, remains largely unexplored.

In this work, we investigate how the relative spatial positioning of H and BH functionalization modulates the electronic structure, stability, and photocatalytic descriptors of PTI-based molecular frameworks. A systematic series of substituted structures was designed by varying the distance between substituents within the π-conjugated backbone, enabling the evaluation of distance-dependent electronic effects.

Density functional theory (DFT) and time-dependent DFT (TD-DFT) calculations were employed to determine ionization energies, excitation energies, CT characteristics, electrophilicity indices, and stability descriptors. Statistical exponential regression models were applied to establish quantitative relationships between substituent separation and key photocatalytic parameters.

The results reveal a clear exponential dependence of ionization energy, stability index, electrophilicity parameters, and CT on substituent distance, demonstrating that relative position acts as a control parameter for tuning the redox properties of carbon nitride systems. These findings provide a rational molecular-level design strategy for improving photocatalytic performance through precise structural engineering.

## Methodology

In this section, we provide a detailed description of the computational details and theoretical descriptors employed in the investigation of the properties of modified PTI. The methodology is organized into four main steps: firstly, the technical details of the simulations ("Computational Details"); secondly, the definition of global stability and reactivity descriptors ("Stability and Reactivity Descriptors"); thirdly, the evaluation of thermodynamic potentials for photocatalysis and CT analysis ("Photocatalytic Performance Analysis"); and finally, the validation of the applied theoretical level ("Methodology Calibration"). It should be noted that, due to the breadth of the analyses, only the most representative data and figures are presented in the main text. An extended set of data, including atomic coordinates and detailed orbital analyses, is found in Supplementary Information (SI).

### Computational details

Geometry optimizations and vibrational frequency analyses of all ground-state structures were performed in the gas phase using density functional theory (DFT) with the ωB97X-D [39, 40] functional and the def2-SV(P) basis set [41]. Vertical excitations were calculated using time-dependent DFT (TD-DFT) [42–44] with the same functional and basis set described above. For each structure, the lowest 10 singlet excited states (S_1_–S_10_) were computed, and the vertical S_0_→S_1_ excitation energy was used as the kinetic excitation descriptor in the ε^3^ stability index (Equation 1). The ωB97X-D functional was employed in all DFT and TD-DFT [42, 45] calculations due to its hybrid formulation with long-range correction, which effectively describes local and charge transfer excitations in conjugated organic structures. These functional combines 100% Hartree-Fock exchange at long range with a generalized gradient approximation (GGA) at short range, together with an empirical dispersion correction, and generally provides an accurate treatment of noncovalent and delocalized electronic interactions [39, 46].

The ionization energy, defined as the energy required to remove the weakest bound electron, was calculated vertically in all cases using the ground-state geometry of the neutral structure. The electron affinity, defined as the energy change of a neutral atom upon addition of an electron, was also calculated using the ground-state geometry. All electronic structure calculations were performed using the Gaussian 16 software package [47].

### Stability and reactivity descriptors

The ε^3^ stability ranking function was also calculated in this study [48]. This is a stability classification metric that can distinguish “magic numbers” in clusters and most stable isomers of molecules according to experimental results. The ε^3^ stability function includes both kinetic and thermodynamic aspects of stability in terms of three properties (Eq. 1). The properties that indicate reactivity and serve as kinetic descriptors include the ionization energy (IE) and the vertical excitation energy of the first singlet excited state (E_S0→S1_). In the original formulation of the ε^3^ function [48], this energy descriptor reflects the energetic separation between the ground and low-lying electronic states and is used as a measure of the kinetic stability of the system. Since ε^3^ is a relative ranking function, the consistent use of the spin-allowed S_0_→S_1_ energy throughout the series preserves the stability trends captured by the index. The thermodynamic descriptor corresponds to the Gibbs free energy of atomization (ΔG°_atom_, 298 K).

1 $${\varepsilon }^{3}=\left|\mathrm{I}\mathrm{E}\right|\cdot \left|{E}_{S0-S1}\right|\cdot \left|\Delta {G}_{atom,298K}^{^\circ }\right|$$

The reactivity of the studied molecules was evaluated using the net electrophilicity index Δω^±^ (Eq. 2)[49, 50], which quantifies the electrophilicity of a molecule relative to its own nucleophilicity. Higher Δω^±^ values indicate a more reactive molecule with electrophilic character, while lower Δω^±^ values also indicate high reactivity, but with nucleophilic character. The electrophilic power (ω⁺), which represents the capacity of a molecule to accept charge, was calculated according to Eq. 3, while the nucleophilic power (ω⁻), representing the tendency to donate charge, was determined using Eq. 4. Both indices are derived from the vertical ionization energy (IE) and electron affinity (EA). These parameters allow for the determination of the net electrophilicity index (Δω^±^), providing a quantitative scale to classify the molecules as predominantly electrophilic (high Δω^±^) or nucleophilic (low Δω^±^) in character.

2 $$\Delta\omega^\pm=\omega^+-\frac1{\omega^-}$$

3 $$\omega^+=\frac{\left(IE+3EA\right)^2}{16\left(IE-EA\right)}$$

4 $$\omega^-=\frac{\left(3IE+EA\right)^2}{16\left(IE-EA\right)}$$

### Photocatalytic performance analysis

Charge transfer in the excited states of PTI was analyzed using the TheoDORE program [51, 52], by performing a Löwdin-type population analysis of the squared elements of the transition density matrix using partial summations that define the matrix ${\Omega}_{AB}^{n\alpha }$ (Eq. 5) [51, 52]:

5 $${\Omega}_{AB}^{n\alpha }=\frac{1}{2}\sum_{a\epsilon A}\sum_{b\epsilon B}{({D}^{n\alpha ,\left[AO\right]}{S}^{\left[AO\right]})}_{ab} {({S}^{\left[AO\right]}{D}^{n\alpha ,\left[AO\right]})}_{ab}$$

where ${D}^{n\alpha ,\left[AO\right]}$ represents the one-particle transition density matrix, from state *n* to state α; *A* and *B* denote general definitions of molecular fragments; ${S}^{\left[AO\right]}$ is the overlap matrix in the atomic orbital (AO) basis, and the labels *a* and *b* refer to atomic orbitals.

**Fig. 2:**
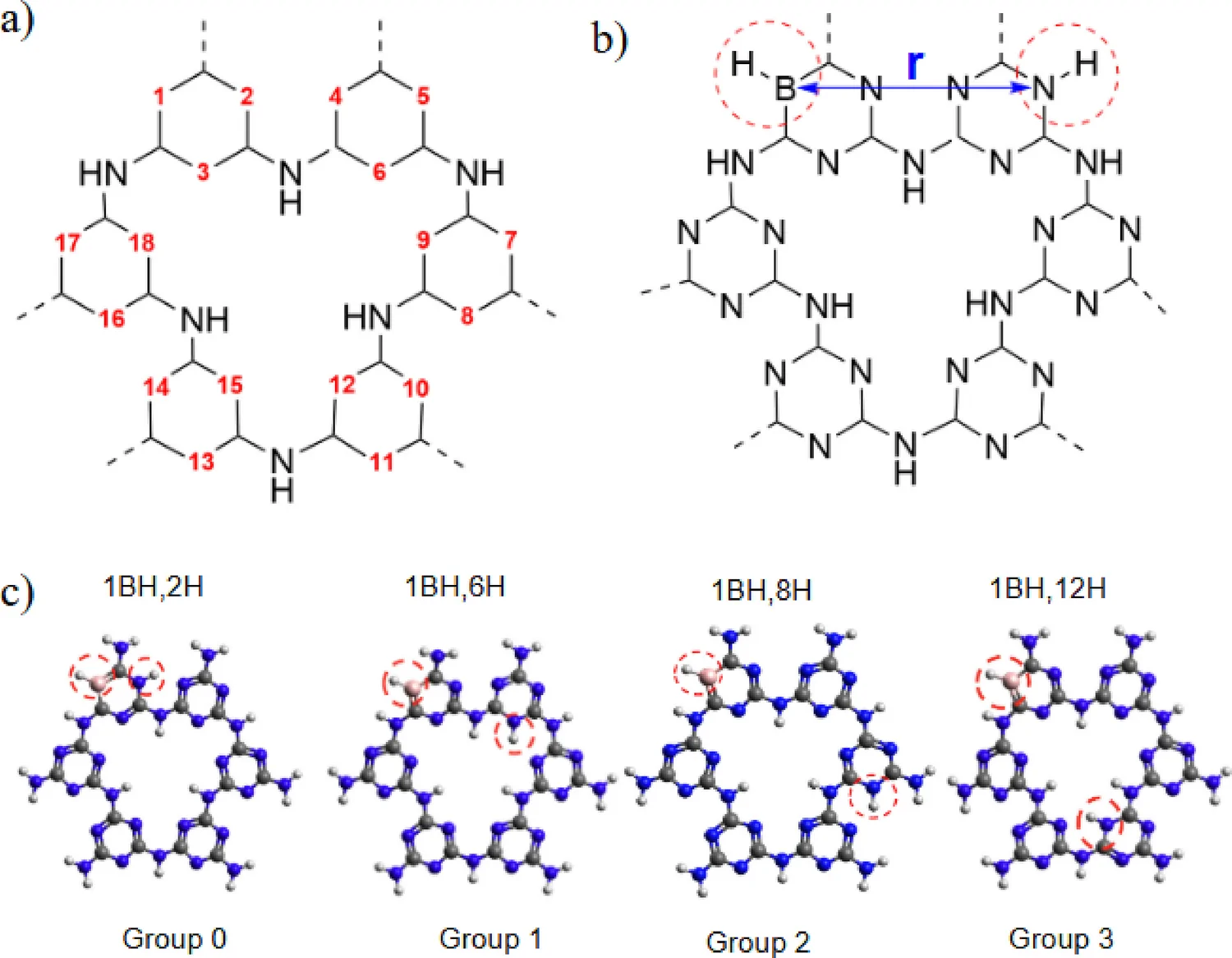
**a** Nitrogen atom numbering scheme used to define the nomenclature of NH- and BH-substituted PTI structures and their classification according to the relative position of the substituents. **b** Definition of the continuous structural descriptor given by the distance (Å) between BH and NH groups, including an illustrative example of how the substitution distances were determined. For the charge-transfer analysis, each PTI ring was treated as an individual fragment; the BH-substituted ring was defined as Fragment I and the subsequent fragments were numbered clockwise. **c** Representative examples of four substituted structures, each corresponding to a distinct group, with the substitution sites highlighted in red

The studied systems were classified according to the relative position of the functionalization, with one BH substituting a N atom, and one H added to a N atom. In Figure 2a, the nitrogen numbering is shown, which serves as the basis for defining the nomenclature of the substituted PTI structures. The grouping was defined based on the number of rings separating these substituents within the molecular structure. Group 0 corresponds to structures in which both substituents are located on the same ring (represented in blue in this study). Group 1 includes structures with one ring between the substituents (represented in red), Group 2 with two rings (represented in green), and Group 3 with three rings of separation (represented in black), see Figure S1 in Supplementary Information. The functionalized derivatives are denoted as PTI-*n*BH,*m*H, where n and m (ranging from 1 to 18) identify the specific functionalization sites within the PTI framework as numbered in Figure 2.

In addition to this categorical classification, a continuous descriptor was also considered, defined as the distance in angstroms between the BH and NH groups (Figure 2b). For the CT analysis, the fragments were defined based on the PTI rings, where each ring represents one fragment. Each PTI ring was treated as an individual fragment; the ring substituted with BH was always defined as Fragment I, and the remaining fragments were numbered sequentially in the clockwise direction (Fragments II–VI), as illustrated in Figure S1 of the SI.

Statistical analysis was performed to evaluate the relationship between substitution distance and the electronic properties of the studied structures [53]. Exponential regression models were applied to correlate the ionization energy and the ε^3^ stability index with the substitution distance, aiming to identify possible distance-dependent trends associated with electronic effects in π-conjugated systems. The quality of the fits was assessed using the coefficient of determination (R^2^), which quantifies the proportion of variance explained by the model.

### Methodology calibration

To calibrate the methodology employed in this work, comparisons were performed with other computational approaches. The calculations were carried out for the unsubstituted PTI, as well as for one representative PTI structure from each group. These four structures were selected due to the strong correlation observed within each group and to optimize computational cost, since extending the higher-level treatments to the full set of structures would be much more computationally demanding. Excited states were computed using different methodologies for these representative systems, chosen to probe two independent sources of error simultaneously: basis-set size and the description of charge-transfer excitations. The first calibration was performed using the CAM-B3LYP functional with empirical D3 [54] dispersion correction and the def2-TZVP basis set [41], as implemented in Gaussian 16; its range-separated formulation provides a balanced description of the charge-transfer states that dominate the present systems, for which conventional hybrid functionals are known to be less reliable. Additionally, SOS-ADC2 (Spin-Opposite Scaling Algebraic Diagrammatic Construction to second order) [55] calculations were carried out using TURBOMOLE [56] and the def2-TZVP basis set, providing a correlated wavefunction reference that is independent of the choice of exchange–correlation functional and well suited to charge-transfer excitations. The D1 diagnostic was evaluated, and all values were found to lie within the reliable range, confirming the predominantly single-reference character of the ground state and supporting the validity of the ADC(2) description. All methods show good agreement in the S_1_ energies and bright states, including the oscillator strengths that define the bright/dark character of each state, and these results are presented in Figure S2 in the SI.

## Results

In this section, the effects of positional substitution of NH groups by BH in the PTI structure are analyzed from two fundamental perspectives. First, in Section "Structural stability and substitution patterns" (Structural Stability and Substitution Patterns), a statistical analysis is performed, in which the discussion focuses on how the substitution topology determines thermodynamic stability and reactivity descriptors in the ground state, considering the distinct groups. In the following section, Section 3.2"UV–Vis Absorption Spectra, and thermodynamics and Photocatalytic Efficiency", the focus shifts to the material’s response to radiation and the nature of the electronic transitions, with particular emphasis on charge separation, as well as on thermodynamics and photocatalytic efficiency. This is an individual analysis, in which one PTI structure from each distinct group is selected to represent its group, chosen based on their distinct properties. It should be noted that, to ensure the conciseness of the main text, only the most relevant discussions are presented here. A comprehensive set of Supplementary Information, including atomic coordinates, orbital analyses, and spectra, is available in the SI, comprising Figures S1–S58 and Tables S1–S50.

### Structural stability and substitution patterns

**Fig. 3:**
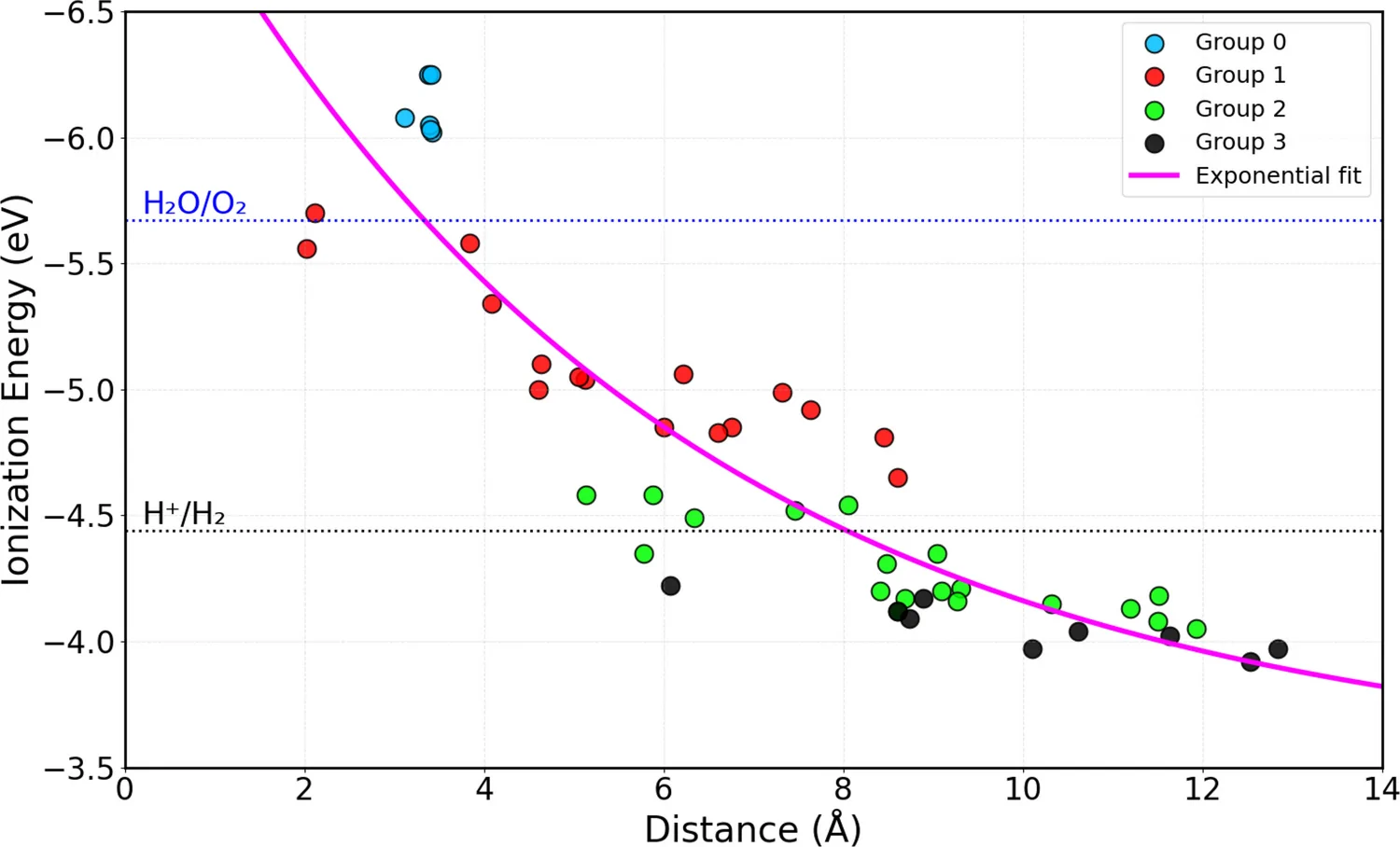
Relationship between ionization energy and substituent separation distance for the studied structures. The data are grouped according to the number of rings between NH and BH substituents: Group 0 (same ring - blue), Group 1 (one intervening ring - red), Group 2 (two rings - green), and Group 3 (three rings - black). The magenta curve represents the exponential regression fit to all data points. The horizontal dashed lines indicate the thermodynamic redox potentials referenced to the vacuum level: H⁺/H₂ (−4.44 eV) and H_2_O/O_2_ (−5.67 eV)

The exponential dependence between ionization potential and substitution distance is shown in Figure 3. The reduction potentials (H⁺/H_2_) are highlighted in black, correlating the ionization energy with the S_0_ level of the molecular structure. Structures positioned above this level (ionization energy lower than −4.44 eV) cannot reduce water. An analogous behavior is observed for the H_2_O/O_2_ pair, highlighted in blue, where values lower than −5.67 eV are not sufficient to oxidize water. These results indicate that structures capable of both reducing and oxidizing water must present ionization energy values higher than −5.67 eV, and only group 0 satisfies these conditions. Group 3 is unable to promote either water reduction or oxidation. In contrast, Group 1 reaches sufficient potential only for water oxidation. Most systems in Group 2 achieve both oxidation and reduction potential simultaneously, a behavior also observed for Group 3 under certain conditions (see Figure 3).

The observed exponential dependence between ionization energy and substitution distance reflects the short-range nature of electronic interactions in π-conjugated systems. When the substituent is in close spatial proximity, greater orbital overlap and stronger electronic coupling occur. As the distance between substituents increases, the direct electronic influence of the substituent on the π-system becomes progressively weaker. This reduction in local interaction decreases the perturbation of the ground-state electron density, favoring a more homogeneous electronic redistribution after ionization. Consequently, electron removal becomes energetically less demanding, leading to a decrease in the ionization energy.

The coefficient of determination obtained for the ionization energy was R^2^ = 0.81, indicating a reasonable quality of fit and suggesting that the trend in ionization energy is governed by the substituent distance. This result supports that the IE-distance relationship follows exponential behavior, characteristic of the spatial decay of molecular electronic interactions and molecular orbital overlap, associated with the progressive weakening of inductive effects and orbital coupling as the separation between the substituent increases.

A statistical analysis was also performed for each group individually. Group 1 presented an R^2^ value of 0.84, Group 2 showed an R^2^ of 0.69, and Group 3 exhibited an R^2^ of 0.82. Group 0 displayed very closely clustered values, resulting in an R^2^ approximately equal to 1. The statistical information for each group is presented in Figure S3.

**Fig. 4:**
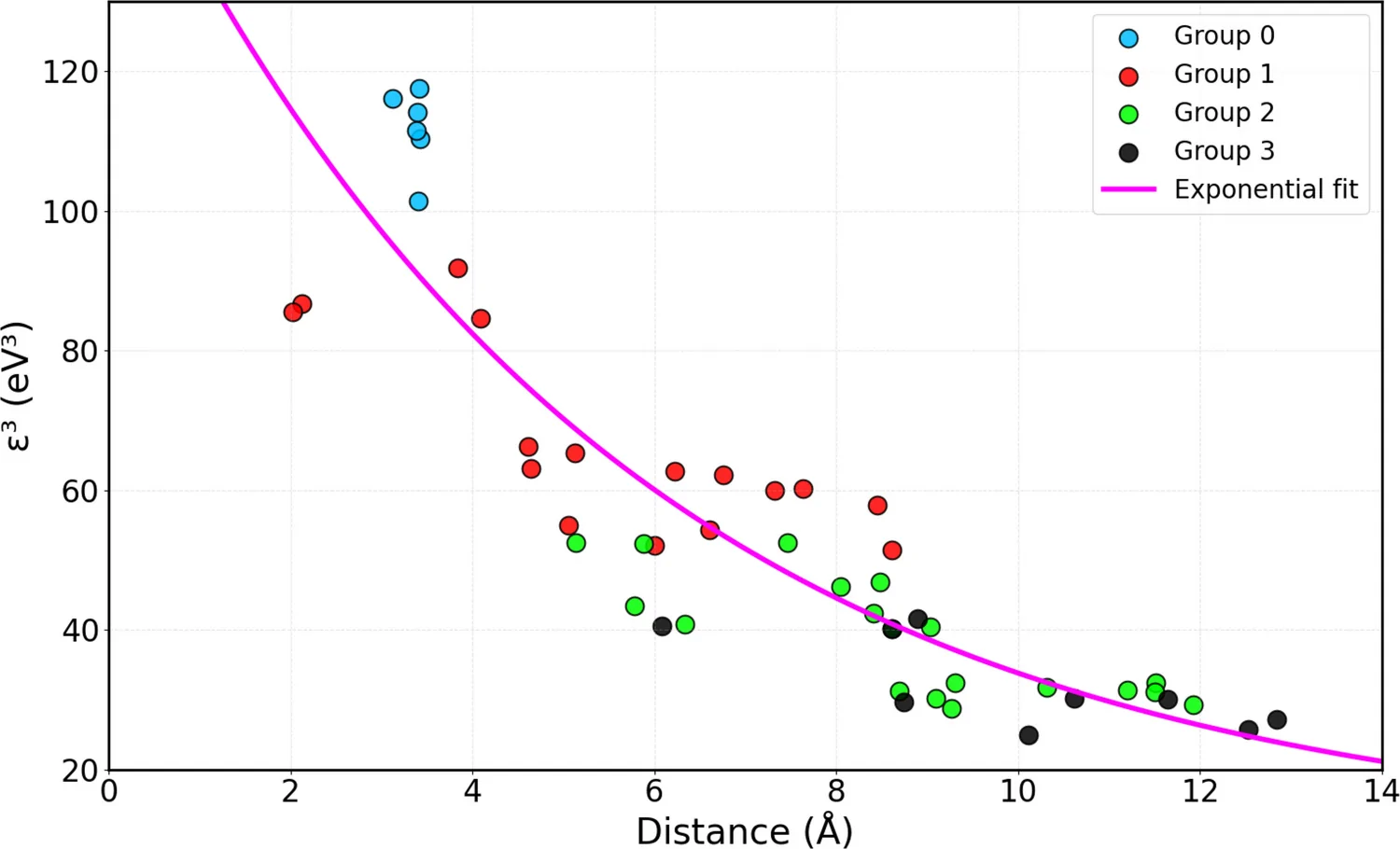
Dependence of the ε^3^ stability index on the substituent separation distance for the PTI substitutions structures. The magenta curve represents the exponential regression fitted to all data points

A similar analysis was performed using the ε^3^ stability index, as shown in Figure 4. The dependence of the ε^3^ stability index on substitution distance is also well described by an exponential model. The coefficient of determination obtained (R^2^ = 0.80) also indicates a reasonable fit, demonstrating that approximately 80% of the variation in the ε^3^ index is explained by the substitution distance.

**Fig. 5:**
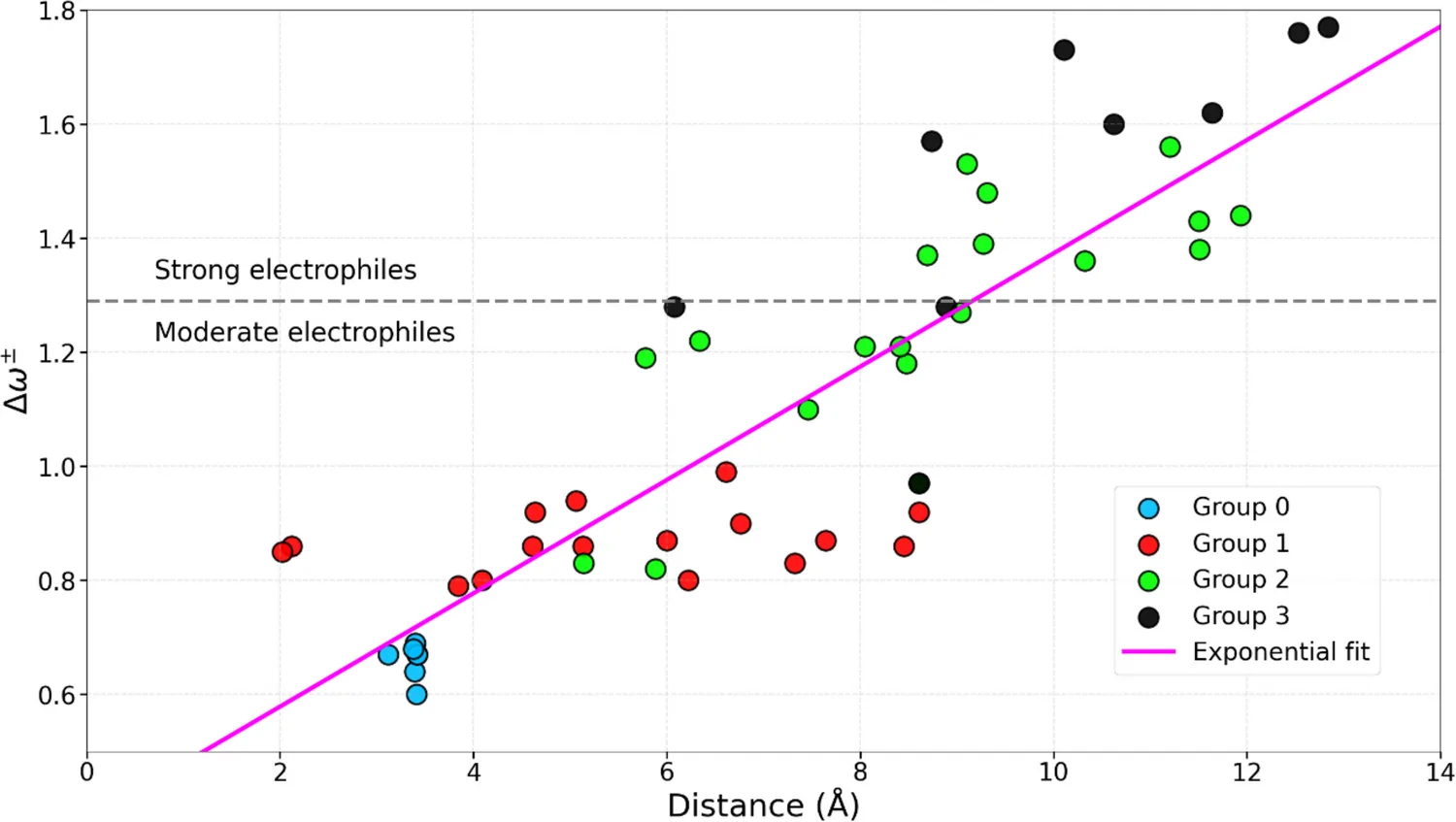
Variation of the net electrophilicity index (Δω±) as a function of the substituent separation distance. The horizontal dashed line indicates the approximate boundary between moderate and strong electrophilic character

The dependence between net electrophilicity index and substitution distance is shown in Figure 5. It is observed that the electrophilicity index increases progressively with substitution distance, indicating a trend opposite that observed for ionization energy and the ε^3^ index. The linear fit adequately describes this increasing behavior (R^2^ = 0.75), revealing a clear correlation between spatial separation and the electrophilic character of the system, comparable in quality to the fits obtained for the ionization energy (R^2^ = 0.83) and the ε^3^ index (R^2^ = 0.81).The Δω^±^ index is associated with the system’s ability to stabilize additional positive charge. Higher values indicate more electrophilic species that are more prone to accept electrons. Group 0 presents the lowest Δω^±^ values, indicating weak electrophilic character; Group 1 exhibits moderate electrophilicity; Group 2 shows a significant increase in electrophilic character; and Group 3 corresponds to strongly electrophilic species.

Upon photon absorption, which leads to CT, the lifetime of photogenerated charge carriers is typically shorter than one nanosecond. This rapid electron-hole recombination hinders photocatalytic reactions [57]. To extend this lifetime, effective spatial separation of charge carriers is essential [58]. In this context, we analyzed the CT behavior of structures with shorter substituent separation (Groups 0 and 1) in comparison with those exhibiting larger spatial separation between substituents (Groups 2 and 3). The results shown in Figure 7 highlight a clear trend in the CT character as a function of substituent separation.

Group 0 presents the lowest average value (0.382 ± 0.050), indicating a more localized excitation, with limited separation between hole and electron densities. As the distance between substituents increases, a significant enhancement in CT is observed. Group 1 already shows a substantial increase (0.817 ± 0.068), suggesting a more pronounced spatial separation of the excited-state density. One representative structure from each group was selected, for which the NTOs were also analyzed.

This trend becomes even more evident for Groups 2 and 3. Group 2 reaches an average value of 0.971 ± 0.070, indicating a nearly complete CT character, while Group 3 exhibits a value of 0.998 ± 0.001, consistent with an almost fully separated electron-hole distribution. In addition to the increase in magnitude, a progressive reduction in the standard deviation is observed, particularly for Group 3, reflecting a highly consistent behavior among the structures in this group.

**Fig. 6:**
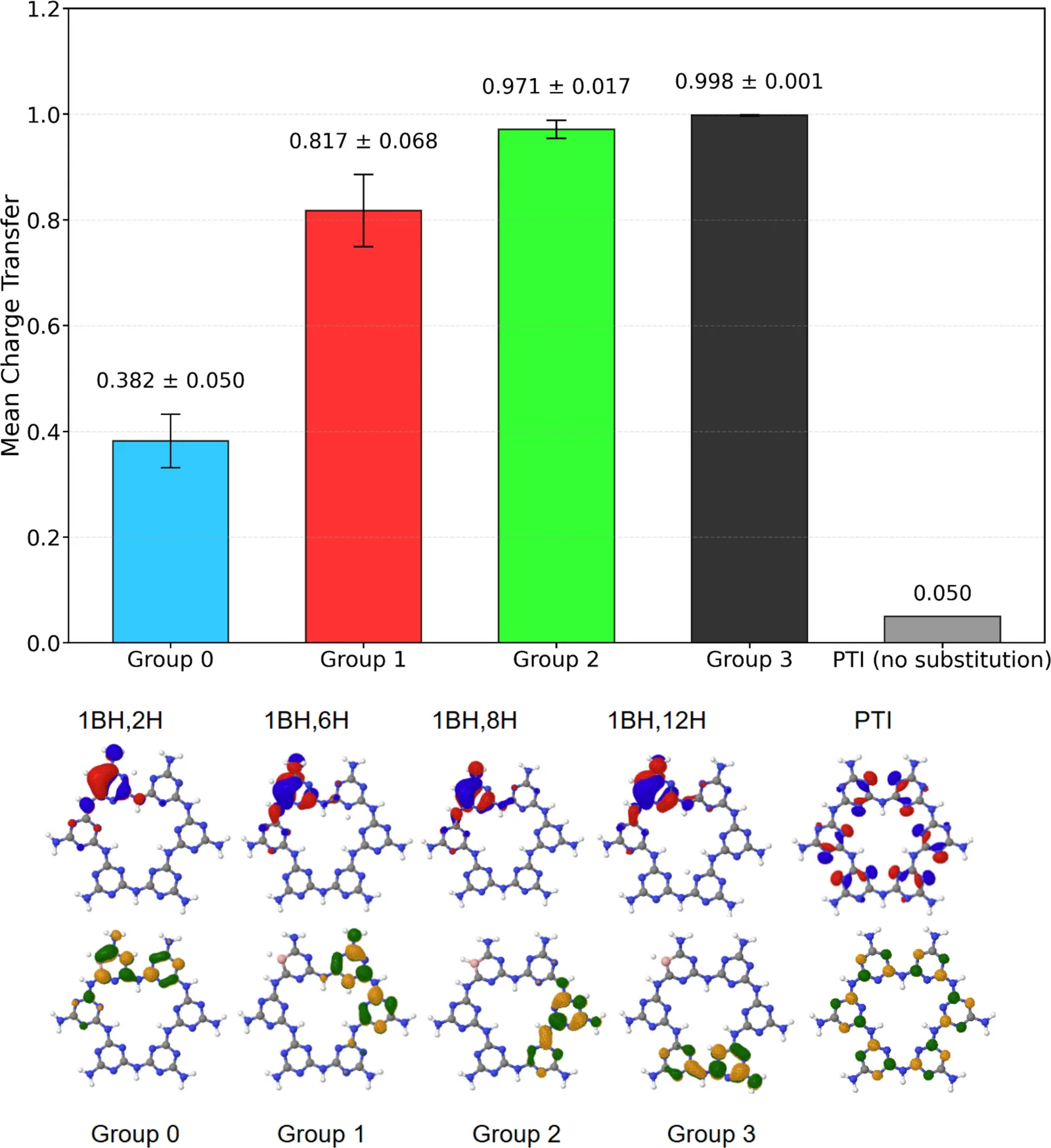
Analysis of the average charge transfer character for each group in the first excited state (S_1_). Error bars represent the standard deviation within each group. Representative natural transition orbitals (NTOs) for S_1_ are also shown, where blue and red surfaces indicate electrons and green and yellow surfaces indicate holes. The unsubstituted PTI structure is included for comparison and does not exhibit a well-localized charge transfer region

The representative NTOs shown in Figure 6 further support this interpretation, clearly illustrating the spatial separation between hole and electron densities as the substituent distance increases. For the other structures within each group, the corresponding NTOs are provided in the SI (Figure S4-S53, and Table S1-S50), confirming that this behavior is maintained across all systems.

### UV–Vis absorption spectra and thermodynamics and photocatalytic efficiency

**Fig. 7:**
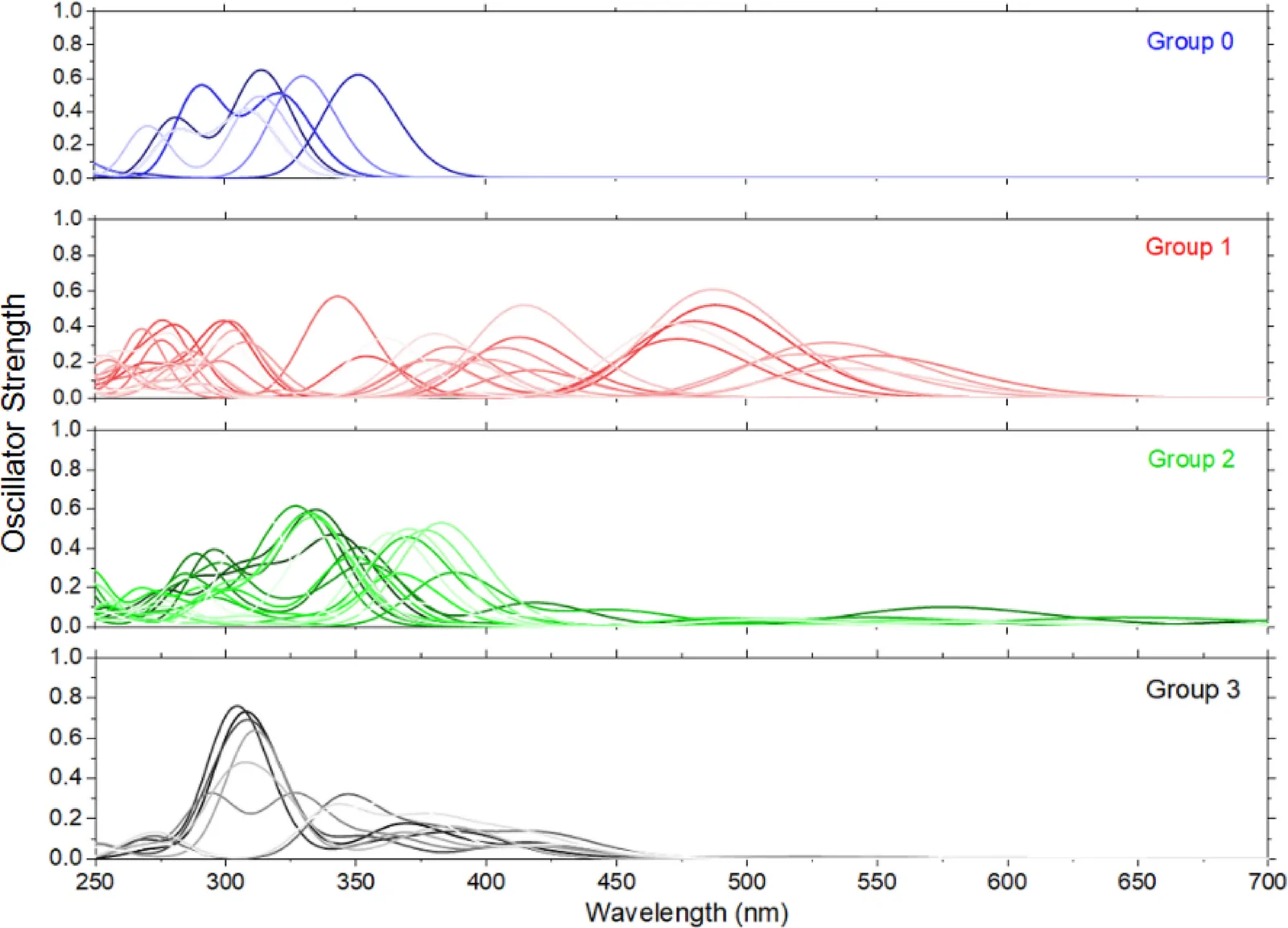
Simulated UV–Vis absorption spectra of the studied structures grouped according to the relative position of NH and BH substituents: Group 0 (same ring - blue), Group 1 (one intervening ring - red), Group 2 (two rings - green), and Group 3 (three rings - black). Each curve corresponds to an individual structure within the respective group

A similar influence is observed in the structural absorption spectra, in Figure 7. Analogously, the spectra of the structures belonging to Group 0 are shown in blue, those of Group 1 in red, those of Group 2 in green, and the absorption spectra of the structures comprising Group 3 in black.

For Group 0, the spectra exhibit narrow bands predominantly concentrated in the near-UV region ≈280–350 nm (≈ 4.43–3.54 eV), indicating more localized electronic transitions and larger energy gaps. For Group 1, a greater dispersion of bands is observed, along with the emergence of absorptions at longer wavelengths (up to ~500 nm (2.48 eV)), suggesting increased electronic delocalization. For Group 2, the spectra become broader and shift toward longer wavelengths, with absorption tails extending into the visible region. For Group 3, although the most intense peak is observed (indicating higher absorption intensity), this absorption remains centered around ~300 nm. It should be stressed that, for the larger-separation structures (Groups 2 and 3), the lowest excited state S_1_ is a dark charge-transfer state with a nearly vanishing oscillator strength (f ≈ 0.000), in agreement with the pronounced electron-hole separation revealed by the NTO analysis (Figure 6). Consequently, the intense absorption does not arise from S_1_ but from higher-lying bright states, whose indices, vertical excitation energies, and oscillator strengths are reported in Table S51 of the SI. In addition to this dominant bright transition, several other excited states carry small but non-negligible oscillator strengths; after the Gaussian broadening applied to simulate the spectra, these secondary transitions overlap with the main band and together define the overall shape of the UV–Vis profile in Figure 7.

**Fig. 8:**
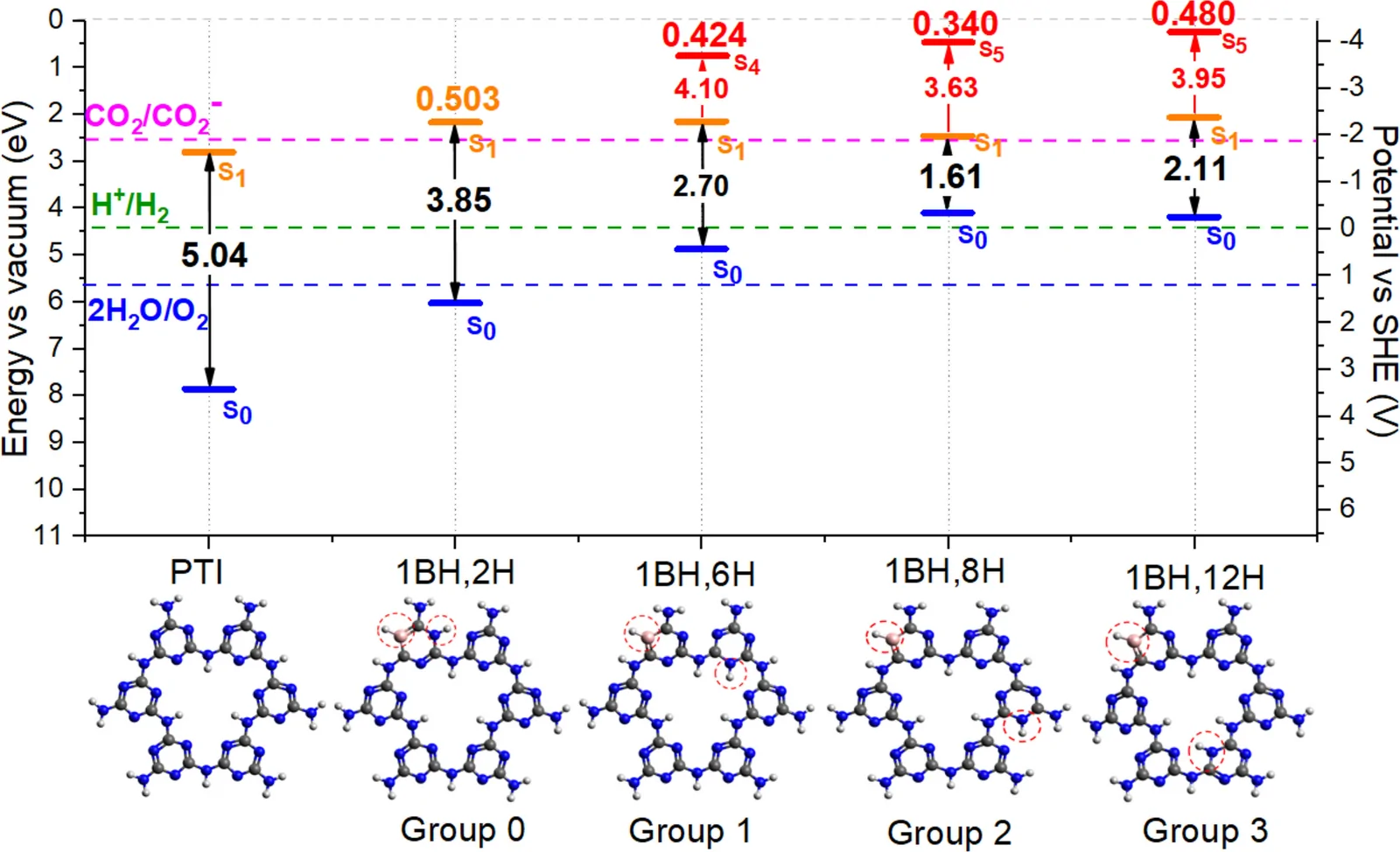
Thermodynamic criteria for PTI substituted structures. The blue level represents the ionization energy level, the orange level corresponds to the first excited-state energy level, and the red level represents the excited state with the highest oscillator strength (bright state), with the actual oscillator strength on top of the red level. To verify the complete list of energy and property values, as well as the associated orbitals, refer to Tables S1-S50 and Figure S4-S53 in the SI

Thermodynamic criteria were also evaluated for all substituted PTI systems and detailed by group in the SI (Figures S54, S55, S56, and S57). In Figure 8, the thermodynamic criteria are shown for PTI, PTI-1BH,2H from Group 0, PTI-1BH,6H from Group 1, PTI-1BH,8H from Group 2, and PTI-1BH,2H from Group 3.

Only bright states up to 5 eV were considered; therefore, PTI does not exhibit a bright S₁ state in this Figure (which occurs at 5.04 eV). All structures in Group 0 present bright states at S_1_. For Group 1, a lower S_0_ level is observed, and this trend continues progressively up to Group 3, in agreement with the previously established relationship between ionization energy (S_0_ level) and substitution distance. All substituted structures exhibit bright states.

To further elucidate the microscopic origin of the distance-dependent ionization energy, an analysis of intrinsic bond orbitals (IBOs) [59]was performed with a focus on the π framework near the substitution sites. The IBO results reveal that the substitution of nitrogen by boron induces a pronounced delocalization of π electron density toward adjacent carbon atoms, as evidenced by the spatial expansion of the orbital lobes. Representative IBOs illustrating this behavior are shown in Figure 58 in the SI. This effect is accompanied by a significant increase in the energies of the σ orbitals in the boron-substituted systems (from −0.24 to −0.37 eV) compared to the unsubstituted structure (≈ −0.50 eV), indicating a local destabilization of the bonding framework. This destabilization arises from the electron-accepting character of boron and the resulting symmetry breaking, which weakens the π-bonding interactions in its vicinity. It is important to note that this local electronic perturbation provides insight into the exponential dependence of the ionization energy on substitution distance. At short distances (Group 0), strong orbital overlap between the substituents and the π-conjugated framework leads to enhanced electronic coupling and a perturbation of the ground-state electron density, resulting in higher ionization energies. As separation increases, this interaction decays exponentially due to the short-range nature of orbital overlap, reducing the influence of the substituents on the electronic structure. Consequently, the system approaches a more homogeneous electronic distribution, decreasing the ionization energy and increasing the electrophilic character, in agreement with the trends observed for Δω^±^ and the CT behavior.

## Conclusions

The results of this study demonstrate that the relative position of NH and BH substituents in PTI structures plays a central role in determining their electronic and photocatalytic properties. An exponential dependence was observed between substitution distance and the photocatalytic descriptors explored here, including ionization energy, ε^3^ stability index, and electrophilicity, indicating that electronic interactions in these π-conjugated systems are strongly governed by spatial effects.

Structures with shorter substitution distances (Group 0) exhibit higher ionization energies and are the only ones that simultaneously satisfy the thermodynamic requirements for both water reduction and oxidation. In contrast, increasing the separation between substituents leads to a progressive decrease in ionization energy and stability, accompanied by an increase in electrophilic character. This behavior is consistent with the weakening of orbital coupling and inductive effects as spatial separation increases.

In addition, the CT analysis revealed a systematic transition from localized excitations to nearly complete charge separation as the distance between substituents increases. The NTO analysis supports this trend, showing a spatial separation between hole and electron densities for structures with larger substitution distances. This indicates that the positioning of substituents can be used as a strategy to control excited-state properties.

Overall, these findings establish a direct relationship between molecular structure and photocatalytic behavior, highlighting substitution distance as a design parameter to tune the electronic structure, stability, and reactivity of carbon nitride-based materials. This provides valuable insight into the rational design of efficient photocatalysts for water splitting applications.

As future perspectives, the results presented here open new possibilities for the rational design of carbon nitride-based photocatalysts through precise control of substituent positioning. Further investigations may explore the extension of this distance-dependent tuning strategy to periodic models and extended frameworks, enabling a more direct connection with experimentally accessible materials.

## Supplementary Information

Below is the link to the electronic supplementary material.

ESM 1 (DOCX 12.2 MB)

## Data Availability

Supporting Information is available for this article and includes: natural transition orbital (NTO) plots corresponding to the relevant electronic transitions; figures of each investigated molecular structure along with their optimized geometries; thermodynamic criteria calculated for each analyzed group; and comprehensive details regarding the methodological calibration procedures.
