# Supplementary material for: Electronic properties of poly (triazine imide) distance-controlled of the NH and BH substitutions and their impacts on the photocatalytic descriptors
Source: J Mol Model. 2026 Oct 20;32(11):432. doi: 10.1007/s00894-026-07005-x (PMC13683508; doi:10.1007/s00894-026-07005-x)
Supplement: Supplementary file 1 [file 894_2026_7005_MOESM1_ESM.docx]

**Supplementary Information**

**Electronic Properties of Poly (Triazine Imide) Distance-Controlled of the NH and BH Substitutions and Their Impacts on the Photocatalytic Descriptors**

Leticia C. S. Faria^1,2^*; Bárbara M. Brum ^1,2^; Leticia M. S. V. Queiroz^1,2^; Josiane R. C. Silva^1,2^; Francisco B. C. Machado^1,2^; Leonardo T. Ueno^1,2^; Luiz F. A. Ferrão^1,2^*.

^1^ Department of Chemistry, Aeronautics Institute of Technology, São José dos Campos 12228-900, Brazil.

^2^ Advanced Scientific Computing and Modeling Laboratory, Aeronautics Institute of Technology, São José dos Campos 12228-900, Brazil.

*Corresponding authors: Leticia Faria, Luiz Ferrão.

Figure Index

[Figure S1: Fragment definition for the CT analysis based on PTI rings. Each ring corresponds to one fragment, where the BH-substituted ring is defined as Fragment 1 (red), followed clockwise by Fragment 2 (green), Fragment 3 (blue), Fragment 4 (pink), Fragment 5 (black), and Fragment 6 (gray).	12](#_Toc230255678)

[Figure S2: Excitation energies (eV) of the bright state (top) and S_1_ state (bottom) for PTI and BH-substituted derivatives (1BH,2H; 1BH,6H; 1BH,8H; and 1BH,12H), computed at different levels of theory: ωB97X-D/Def2-SV(P) (black), CAM-B3LYP-D3/Def2-TZVP (blue), and SOS-ADC(2)/Def2-TZVP (magenta). The numbers indicate the oscillator strengths associated with each transition.	12](#_Toc230255679)

[Figure S3: Ionization energy (eV) as a function of the BH–NH distance (Å). The data are grouped into four categories (Group 0–3), shown as colored markers. The pink curve represents the global exponential fit, and the shaded area corresponds to the 95% confidence interval.	13](#_Toc230255680)

[Figure S4: Molecular structure of the 1BH,2H structure. The substituted sites are highlighted by red dashed outlines, and the numbers connecting the BH and NH groups indicate the corresponding distances in angstroms (Å).	13](#_Toc230255681)

[Figure S5: Molecular structure of the 1BH, 3H system. The substituted sites are highlighted by red dashed outlines, and the numbers connecting the BH and NH groups indicate the corresponding distances in angstroms (Å).	14](#_Toc230255682)

[Figure S6: Molecular structure of the 3BH, 2H system. The substituted sites are highlighted by red dashed outlines, and the numbers connecting the BH and NH groups indicate the corresponding distances in angstroms (Å).	15](#_Toc230255683)

[Figure S7: Molecular structure of the 2BH, 3H system. The substituted sites are highlighted by red dashed outlines, and the numbers connecting the BH and NH groups indicate the corresponding distances in angstroms (Å).	16](#_Toc230255684)

[Figure S8: Molecular structure of the 3BH, 1H system. The substituted sites are highlighted by red dashed outlines, and the numbers connecting the BH and NH groups indicate the corresponding distances in angstroms (Å).	17](#_Toc230255685)

[Figure S9: Molecular structure of the 2BH, 1H system. The substituted sites are highlighted by red dashed outlines, and the numbers connecting the BH and NH groups indicate the corresponding distances in angstroms (Å).	17](#_Toc230255686)

[Figure S10: Molecular structure of the 1BH, 5H system. The substituted sites are highlighted by red dashed outlines, and the numbers connecting the BH and NH groups indicate the corresponding distances in angstroms (Å).	18](#_Toc230255687)

[Figure S11: Molecular structure of the 1BH, 16H system. The substituted sites are highlighted by red dashed outlines, and the numbers connecting the BH and NH groups indicate the corresponding distances in angstroms (Å).	19](#_Toc230255688)

[Figure S12: Molecular structure of the 1BH, 17H system. The substituted sites are highlighted by red dashed outlines, and the numbers connecting the BH and NH groups indicate the corresponding distances in angstroms (Å).	20](#_Toc230255689)

[Figure S13: Molecular structure of the 1BH, 6H system. The substituted sites are highlighted by red dashed outlines, and the numbers connecting the BH and NH groups indicate the corresponding distances in angstroms (Å).	20](#_Toc230255690)

[Figure S14: Molecular structure of the 1BH, 18H system. The substituted sites are highlighted by red dashed outlines, and the numbers connecting the BH and NH groups indicate the corresponding distances in angstroms (Å).	21](#_Toc230255691)

[Figure S15: Molecular structure of the 3BH, 16H system. The substituted sites are highlighted by red dashed outlines, and the numbers connecting the BH and NH groups indicate the corresponding distances in angstroms (Å).	22](#_Toc230255692)

[Figure S16: Molecular structure of the 2BH, 4H system. The substituted sites are highlighted by red dashed outlines, and the numbers connecting the BH and NH groups indicate the corresponding distances in angstroms (Å).	23](#_Toc230255693)

[Figure S17: Molecular structure of the 2BH, 6H system. The substituted sites are highlighted by red dashed outlines, and the numbers connecting the BH and NH groups indicate the corresponding distances in angstroms (Å).	23](#_Toc230255694)

[Figure S18: Molecular structure of the 2BH, 16H system. The substituted sites are highlighted by red dashed outlines, and the numbers connecting the BH and NH groups indicate the corresponding distances in angstroms (Å).	24](#_Toc230255695)

[Figure S19: Molecular structure of the 2BH, 17H system. The substituted sites are highlighted by red dashed outlines, and the numbers connecting the BH and NH groups indicate the corresponding distances in angstroms (Å).	25](#_Toc230255696)

[Figure S20: Molecular structure of the 3BH, 5H system. The substituted sites are highlighted by red dashed outlines, and the numbers connecting the BH and NH groups indicate the corresponding distances in angstroms (Å).	26](#_Toc230255697)

[Figure S21: Molecular structure of the 3BH, 6H system. The substituted sites are highlighted by red dashed outlines, and the numbers connecting the BH and NH groups indicate the corresponding distances in angstroms (Å).	26](#_Toc230255698)

[Figure S22: Molecular structure of the 3BH, 17H system. The substituted sites are highlighted by red dashed outlines, and the numbers connecting the BH and NH groups indicate the corresponding distances in angstroms (Å).	27](#_Toc230255699)

[Figure S23: Molecular structure of the 3BH, 18H system. The substituted sites are highlighted by red dashed outlines, and the numbers connecting the BH and NH groups indicate the corresponding distances in angstroms (Å).	28](#_Toc230255700)

[Figure S24: Molecular structure of the 2BH, 5H system. The substituted sites are highlighted by red dashed outlines, and the numbers connecting the BH and NH groups indicate the corresponding distances in angstroms (Å).	29](#_Toc230255701)

[Figure S25: Molecular structure of the 2BH, 18H system. The substituted sites are highlighted by red dashed outlines, and the numbers connecting the BH and NH groups indicate the corresponding distances in angstroms (Å).	30](#_Toc230255702)

[Figure S26: Molecular structure of the 1BH, 7H system. The substituted sites are highlighted by red dashed outlines, and the numbers connecting the BH and NH groups indicate the corresponding distances in angstroms (Å).	30](#_Toc230255703)

[Figure S27: Molecular structure of the 1BH, 13H system. The substituted sites are highlighted by red dashed outlines, and the numbers connecting the BH and NH groups indicate the corresponding distances in angstroms (Å).	31](#_Toc230255704)

[Figure S28: Molecular structure of the 1BH, 8H system. The substituted sites are highlighted by red dashed outlines, and the numbers connecting the BH and NH groups indicate the corresponding distances in angstroms (Å).	32](#_Toc230255705)

[Figure S29: Molecular structure of the 1BH, 14H system. The substituted sites are highlighted by red dashed outlines, and the numbers connecting the BH and NH groups indicate the corresponding distances in angstroms (Å).	33](#_Toc230255706)

[Figure S30: Molecular structure of the 1BH, 9H system. The substituted sites are highlighted by red dashed outlines, and the numbers connecting the BH and NH groups indicate the corresponding distances in angstroms (Å).	33](#_Toc230255707)

[Figure S31: Molecular structure of the 1BH, 15H system. The substituted sites are highlighted by red dashed outlines, and the numbers connecting the BH and NH groups indicate the corresponding distances in angstroms (Å).	34](#_Toc230255708)

[Figure S32: Molecular structure of the 3BH, 14H system. The substituted sites are highlighted by red dashed outlines, and the numbers connecting the BH and NH groups indicate the corresponding distances in angstroms (Å).	35](#_Toc230255709)

[Figure S33: Molecular structure of the 3BH, 8H system. The substituted sites are highlighted by red dashed outlines, and the numbers connecting the BH and NH groups indicate the corresponding distances in angstroms (Å).	36](#_Toc230255710)

[Figure S34: Molecular structure of the 2BH, 7H system. The substituted sites are highlighted by red dashed outlines, and the numbers connecting the BH and NH groups indicate the corresponding distances in angstroms (Å).	36](#_Toc230255711)

[Figure S35: Molecular structure of the 2BH, 9H system. The substituted sites are highlighted by red dashed outlines, and the numbers connecting the BH and NH groups indicate the corresponding distances in angstroms (Å).	37](#_Toc230255712)

[Figure S36: Molecular structure of the 2BH, 13H system. The substituted sites are highlighted by red dashed outlines, and the numbers connecting the BH and NH groups indicate the corresponding distances in angstroms (Å).	38](#_Toc230255713)

[Figure S37: Molecular structure of the 2BH, 14H system. The substituted sites are highlighted by red dashed outlines, and the numbers connecting the BH and NH groups indicate the corresponding distances in angstroms (Å).	38](#_Toc230255714)

[Figure S38: Molecular structure of the 2BH, 15H system. The substituted sites are highlighted by red dashed outlines, and the numbers connecting the BH and NH groups indicate the corresponding distances in angstroms (Å).	39](#_Toc230255715)

[Figure S39: Molecular structure of the 3BH, 7H system. The substituted sites are highlighted by red dashed outlines, and the numbers connecting the BH and NH groups indicate the corresponding distances in angstroms (Å).	40](#_Toc230255716)

[Figure S40: Molecular structure of the 3BH, 9H system. The substituted sites are highlighted by red dashed outlines, and the numbers connecting the BH and NH groups indicate the corresponding distances in angstroms (Å).	41](#_Toc230255717)

[Figure S41: Molecular structure of the 3BH, 13H system. The substituted sites are highlighted by red dashed outlines, and the numbers connecting the BH and NH groups indicate the corresponding distances in angstroms (Å).	42](#_Toc230255718)

[Figure S42: Molecular structure of the 3BH, 15H system. The substituted sites are highlighted by red dashed outlines, and the numbers connecting the BH and NH groups indicate the corresponding distances in angstroms (Å).	42](#_Toc230255719)

[Figure S43: Molecular structure of the 2BH, 8H system. The substituted sites are highlighted by red dashed outlines, and the numbers connecting the BH and NH groups indicate the corresponding distances in angstroms (Å).	43](#_Toc230255720)

[Figure S44: Molecular structure of the 2BH, 12H system. The substituted sites are highlighted by red dashed outlines, and the numbers connecting the BH and NH groups indicate the corresponding distances in angstroms (Å).	44](#_Toc230255721)

[Figure S45: Molecular structure of the 1BH, 10H system. The substituted sites are highlighted by red dashed outlines, and the numbers connecting the BH and NH groups indicate the corresponding distances in angstroms (Å).	45](#_Toc230255722)

[Figure S46: Molecular structure of the 1BH, 11H system. The substituted sites are highlighted by red dashed outlines, and the numbers connecting the BH and NH groups indicate the corresponding distances in angstroms (Å).	45](#_Toc230255723)

[Figure S47: Molecular structure of the 1BH, 12H system. The substituted sites are highlighted by red dashed outlines, and the numbers connecting the BH and NH groups indicate the corresponding distances in angstroms (Å).	46](#_Toc230255724)

[Figure S48: Molecular structure of the 3BH, 10H system. The substituted sites are highlighted by red dashed outlines, and the numbers connecting the BH and NH groups indicate the corresponding distances in angstroms (Å).	47](#_Toc230255725)

[Figure S49: Molecular structure of the 2BH, 10H system. The substituted sites are highlighted by red dashed outlines, and the numbers connecting the BH and NH groups indicate the corresponding distances in angstroms (Å).	47](#_Toc230255726)

[Figure S50: Molecular structure of the 2BH, 12H system. The substituted sites are highlighted by red dashed outlines, and the numbers connecting the BH and NH groups indicate the corresponding distances in angstroms (Å).	48](#_Toc230255727)

[Figure S51: Molecular structure of the 2BH, 11H system. The substituted sites are highlighted by red dashed outlines, and the numbers connecting the BH and NH groups indicate the corresponding distances in angstroms (Å).	49](#_Toc230255728)

[Figure S52: Molecular structure of the 3BH, 11H system. The substituted sites are highlighted by red dashed outlines, and the numbers connecting the BH and NH groups indicate the corresponding distances in angstroms (Å).	50](#_Toc230255729)

[Figure S53: Molecular structure of the 3BH, 12H system. The substituted sites are highlighted by red dashed outlines, and the numbers connecting the BH and NH groups indicate the corresponding distances in angstroms (Å).	50](#_Toc230255730)

[Figure S54: Thermodynamic criteria for PTI Group 0 structures. The blue level represents the ionization energy, the orange level corresponds to the first excited-state energy (S_1_), and the red level indicates the excited state with the highest oscillator strength (bright state), with the corresponding oscillator strength values shown above the red level.	51](#_Toc230255731)

[Figure S55: Thermodynamic criteria for PTI Group 1 structures. The blue level represents the ionization energy, the orange level corresponds to the first excited-state energy (S_1_), and the red level indicates the excited state with the highest oscillator strength (bright state), with the corresponding oscillator strength values shown above the red level.	52](#_Toc230255732)

[Figure S56: Thermodynamic criteria for PTI Group 2 structures. The blue level represents the ionization energy, the orange level corresponds to the first excited-state energy (S_1_), and the red level indicates the excited state with the highest oscillator strength (bright state), with the corresponding oscillator strength values shown above the red level.	52](#_Toc230255733)

[Figure S57: Thermodynamic criteria for PTI Group 3 structures. The blue level represents the ionization energy, the orange level corresponds to the first excited-state energy (S_1_), and the red level indicates the excited state with the highest oscillator strength (bright state), with the corresponding oscillator strength values shown above the red level.	52](#_Toc230255734)

[Figure S58: Representative intrinsic bond orbitals (IBOs) highlighting the π-framework in PTI and boron-substituted structures. The orbitals illustrate the delocalization of π electron density toward adjacent carbon atoms upon nitrogen-to-boron substitution, as evidenced by the expansion of the orbital lobes near the substitution sites.	53](#_Toc230255735)

Table Index

[Table S1: Summary of the S_1_ excited state for the 1BH,2H system structure, including the structural name, electron and hole distributions, dominant orbital contributions, charge transfer (C.T.) character, excitation energy (E, in eV), and oscillator strength.	13](#_Toc236878800)

[Table S2: Summary of the S_1_ excited state for the 1BH, 3H system structure, including the structural name, electron and hole distributions, dominant orbital contributions, charge transfer (C.T.) character, excitation energy (E, in eV), and oscillator strength.	13](#_Toc236878801)

[Table S3: Summary of the S_1_ excited state for the 3BH, 2H system structure, including the structural name, electron and hole distributions, dominant orbital contributions, charge transfer (C.T.) character, excitation energy (E, in eV), and oscillator strength.	14](#_Toc236878802)

[Table S4: Summary of the S_1_ excited state for the 2BH, 3H system structure, including the structural name, electron and hole distributions, dominant orbital contributions, charge transfer (C.T.) character, excitation energy (E, in eV), and oscillator strength.	15](#_Toc236878803)

[Table S5: Summary of the S_1_ excited state for the 3BH, 1H system structure, including the structural name, electron and hole distributions, dominant orbital contributions, charge transfer (C.T.) character, excitation energy (E, in eV), and oscillator strength.	16](#_Toc236878804)

[Table S6: Summary of the S_1_ excited state for the 2BH, 1H system structure, including the structural name, electron and hole distributions, dominant orbital contributions, charge transfer (C.T.) character, excitation energy (E, in eV), and oscillator strength.	16](#_Toc236878805)

[Table S7: Summary of the S_1_ excited state for the 1BH, 5H system structure, including the structural name, electron and hole distributions, dominant orbital contributions, charge transfer (C.T.) character, excitation energy (E, in eV), and oscillator strength.	17](#_Toc236878806)

[Table S8: Summary of the S_1_ excited state for the 1BH, 16H system structure, including the structural name, electron and hole distributions, dominant orbital contributions, charge transfer (C.T.) character, excitation energy (E, in eV), and oscillator strength.	18](#_Toc236878807)

[Table S9: Summary of the S_1_ excited state for the 1BH, 17H system structure, including the structural name, electron and hole distributions, dominant orbital contributions, charge transfer (C.T.) character, excitation energy (E, in eV), and oscillator strength.	19](#_Toc236878808)

[Table S10: Summary of the S_1_ excited state for the 1BH, 6H system structure, including the structural name, electron and hole distributions, dominant orbital contributions, charge transfer (C.T.) character, excitation energy (E, in eV), and oscillator strength.	19](#_Toc236878809)

[Table S11: Summary of the S_1_ excited state for the 1BH, 18H system structure, including the structural name, electron and hole distributions, dominant orbital contributions, charge transfer (C.T.) character, excitation energy (E, in eV), and oscillator strength.	20](#_Toc236878810)

[Table S12: Summary of the S_1_ excited state for the 3BH, 16H system structure, including the structural name, electron and hole distributions, dominant orbital contributions, charge transfer (C.T.) character, excitation energy (E, in eV), and oscillator strength.	21](#_Toc236878811)

[Table S13: Summary of the S_1_ excited state for the 2BH, 4H system structure, including the structural name, electron and hole distributions, dominant orbital contributions, charge transfer (C.T.) character, excitation energy (E, in eV), and oscillator strength.	22](#_Toc236878812)

[Table S14: Summary of the S_1_ excited state for the 2BH, 6H system structure, including the structural name, electron and hole distributions, dominant orbital contributions, charge transfer (C.T.) character, excitation energy (E, in eV), and oscillator strength.	22](#_Toc236878813)

[Table S15: Summary of the S_1_ excited state for the 2BH, 16H system structure, including the structural name, electron and hole distributions, dominant orbital contributions, charge transfer (C.T.) character, excitation energy (E, in eV), and oscillator strength.	23](#_Toc236878814)

[Table S16: Summary of the S_1_ excited state for the 2BH, 17H system structure, including the structural name, electron and hole distributions, dominant orbital contributions, charge transfer (C.T.) character, excitation energy (E, in eV), and oscillator strength.	24](#_Toc236878815)

[Table S17: Summary of the S_1_ excited state for the 3BH, 5H system structure, including the structural name, electron and hole distributions, dominant orbital contributions, charge transfer (C.T.) character, excitation energy (E, in eV), and oscillator strength.	25](#_Toc236878816)

[Table S18: Summary of the S_1_ excited state for the 3BH, 6H system structure, including the structural name, electron and hole distributions, dominant orbital contributions, charge transfer (C.T.) character, excitation energy (E, in eV), and oscillator strength.	25](#_Toc236878817)

[Table S19: Summary of the S_1_ excited state for the 3BH, 17H system structure, including the structural name, electron and hole distributions, dominant orbital contributions, charge transfer (C.T.) character, excitation energy (E, in eV), and oscillator strength.	26](#_Toc236878818)

[Table S20: Summary of the S_1_ excited state for the 3BH, 18H system structure, including the structural name, electron and hole distributions, dominant orbital contributions, charge transfer (C.T.) character, excitation energy (E, in eV), and oscillator strength.	27](#_Toc236878819)

[Table S21: Summary of the S_1_ excited state for the 2BH, 5H system structure, including the structural name, electron and hole distributions, dominant orbital contributions, charge transfer (C.T.) character, excitation energy (E, in eV), and oscillator strength.	28](#_Toc236878820)

[Table S22: Summary of the S_1_ excited state for the 2BH, 18H system structure, including the structural name, electron and hole distributions, dominant orbital contributions, charge transfer (C.T.) character, excitation energy (E, in eV), and oscillator strength.	29](#_Toc236878821)

[Table S23: Summary of the S_1_ excited state for the 1BH, 7H system structure, including the structural name, electron and hole distributions, dominant orbital contributions, charge transfer (C.T.) character, excitation energy (E, in eV), and oscillator strength.	29](#_Toc236878822)

[Table S24: Summary of the S_1_ excited state for the 1BH, 13H system structure, including the structural name, electron and hole distributions, dominant orbital contributions, charge transfer (C.T.) character, excitation energy (E, in eV), and oscillator strength.	30](#_Toc236878823)

[Table S25: Summary of the S_1_ excited state for the 1BH, 8H system structure, including the structural name, electron and hole distributions, dominant orbital contributions, charge transfer (C.T.) character, excitation energy (E, in eV), and oscillator strength.	31](#_Toc236878824)

[Table S26: Summary of the S_1_ excited state for the 1BH, 14H system structure, including the structural name, electron and hole distributions, dominant orbital contributions, charge transfer (C.T.) character, excitation energy (E, in eV), and oscillator strength.	32](#_Toc236878825)

[Table S27: Summary of the S_1_ excited state for the 1BH, 9H system structure, including the structural name, electron and hole distributions, dominant orbital contributions, charge transfer (C.T.) character, excitation energy (E, in eV), and oscillator strength.	32](#_Toc236878826)

[Table S28: Summary of the S_1_ excited state for the 1BH, 15H system structure, including the structural name, electron and hole distributions, dominant orbital contributions, charge transfer (C.T.) character, excitation energy (E, in eV), and oscillator strength.	33](#_Toc236878827)

[Table S29: Summary of the S_1_ excited state for the 3BH, 14H system structure, including the structural name, electron and hole distributions, dominant orbital contributions, charge transfer (C.T.) character, excitation energy (E, in eV), and oscillator strength.	34](#_Toc236878828)

[Table S30: Summary of the S_1_ excited state for the 3BH, 8H system structure, including the structural name, electron and hole distributions, dominant orbital contributions, charge transfer (C.T.) character, excitation energy (E, in eV), and oscillator strength.	35](#_Toc236878829)

[Table S31: Summary of the S_1_ excited state for the 2BH, 7H system structure, including the structural name, electron and hole distributions, dominant orbital contributions, charge transfer (C.T.) character, excitation energy (E, in eV), and oscillator strength.	35](#_Toc236878830)

[Table S32: Summary of the S_1_ excited state for the 2BH, 9H system structure, including the structural name, electron and hole distributions, dominant orbital contributions, charge transfer (C.T.) character, excitation energy (E, in eV), and oscillator strength.	36](#_Toc236878831)

[Table S33: Summary of the S_1_ excited state for the 2BH, 13H system structure, including the structural name, electron and hole distributions, dominant orbital contributions, charge transfer (C.T.) character, excitation energy (E, in eV), and oscillator strength.	37](#_Toc236878832)

[Table S34: Summary of the S_1_ excited state for the 2BH, 14H system structure, including the structural name, electron and hole distributions, dominant orbital contributions, charge transfer (C.T.) character, excitation energy (E, in eV), and oscillator strength.	38](#_Toc236878833)

[Table S35: Summary of the S_1_ excited state for the 2BH, 15H system structure, including the structural name, electron and hole distributions, dominant orbital contributions, charge transfer (C.T.) character, excitation energy (E, in eV), and oscillator strength.	38](#_Toc236878834)

[Table S36: Summary of the S_1_ excited state for the 3BH, 7H system structure, including the structural name, electron and hole distributions, dominant orbital contributions, charge transfer (C.T.) character, excitation energy (E, in eV), and oscillator strength.	39](#_Toc236878835)

[Table S37: Summary of the S_1_ excited state for the 3BH, 9H system structure, including the structural name, electron and hole distributions, dominant orbital contributions, charge transfer (C.T.) character, excitation energy (E, in eV), and oscillator strength.	40](#_Toc236878836)

[Table S38: Summary of the S_1_ excited state for the 3BH, 13H system structure, including the structural name, electron and hole distributions, dominant orbital contributions, charge transfer (C.T.) character, excitation energy (E, in eV), and oscillator strength.	41](#_Toc236878837)

[Table S39: Summary of the S_1_ excited state for the 3BH, 15H system structure, including the structural name, electron and hole distributions, dominant orbital contributions, charge transfer (C.T.) character, excitation energy (E, in eV), and oscillator strength.	41](#_Toc236878838)

[Table S40: Summary of the S_1_ excited state for the 2BH, 8H system structure, including the structural name, electron and hole distributions, dominant orbital contributions, charge transfer (C.T.) character, excitation energy (E, in eV), and oscillator strength.	42](#_Toc236878839)

[Table S41: Summary of the S_1_ excited state for the 2BH, 12H system structure, including the structural name, electron and hole distributions, dominant orbital contributions, charge transfer (C.T.) character, excitation energy (E, in eV), and oscillator strength.	43](#_Toc236878840)

[Table S42: Summary of the S_1_ excited state for the 1BH, 10H system structure, including the structural name, electron and hole distributions, dominant orbital contributions, charge transfer (C.T.) character, excitation energy (E, in eV), and oscillator strength.	44](#_Toc236878841)

[Table S43: Summary of the S_1_ excited state for the 1BH, 11H system structure, including the structural name, electron and hole distributions, dominant orbital contributions, charge transfer (C.T.) character, excitation energy (E, in eV), and oscillator strength.	44](#_Toc236878842)

[Table S44: Summary of the S_1_ excited state for the 1BH, 12H system structure, including the structural name, electron and hole distributions, dominant orbital contributions, charge transfer (C.T.) character, excitation energy (E, in eV), and oscillator strength.	45](#_Toc236878843)

[Table S45: Summary of the S_1_ excited state for the 3BH, 10H system structure, including the structural name, electron and hole distributions, dominant orbital contributions, charge transfer (C.T.) character, excitation energy (E, in eV), and oscillator strength.	46](#_Toc236878844)

[Table S46: Summary of the S_1_ excited state for the 2BH, 10H system structure, including the structural name, electron and hole distributions, dominant orbital contributions, charge transfer (C.T.) character, excitation energy (E, in eV), and oscillator strength.	47](#_Toc236878845)

[Table S47: Summary of the S_1_ excited state for the 2BH, 12H system structure, including the structural name, electron and hole distributions, dominant orbital contributions, charge transfer (C.T.) character, excitation energy (E, in eV), and oscillator strength.	47](#_Toc236878846)

[Table S48: Summary of the S_1_ excited state for the 2BH, 11H system structure, including the structural name, electron and hole distributions, dominant orbital contributions, charge transfer (C.T.) character, excitation energy (E, in eV), and oscillator strength.	48](#_Toc236878847)

[Table S49: Summary of the S_1_ excited state for the 3BH, 11H system structure, including the structural name, electron and hole distributions, dominant orbital contributions, charge transfer (C.T.) character, excitation energy (E, in eV), and oscillator strength.	49](#_Toc236878848)

[Table S50: Summary of the S_1_ excited state for the 3BH, 12H system structure, including the structural name, electron and hole distributions, dominant orbital contributions, charge transfer (C.T.) character, excitation energy (E, in eV), and oscillator strength.	49](#_Toc236878849)

[Table S 51: Vertical excitation energy and oscillator strength (f) of the lowest singlet state (S_1_) and of the bright state (S_0_→S_n_, lowest state with significant f).	50](#_Toc236878850)

Figure S1**:** Fragment definition for the CT analysis based on PTI rings. Each ring corresponds to one fragment. The BH-substituted ring is defined as Fragment I, and the remaining fragments are numbered sequentially in the clockwise direction (Fragments II–VI). This numbering illustrates the general convention; for each substituted structure, Fragment I always corresponds to the ring bearing the BH group.

Figure S2: Excitation energies (eV) of the bright state (top) and S_1_ state (bottom) for PTI and BH-substituted derivatives (1BH,2H; 1BH,6H; 1BH,8H; and 1BH,12H), computed at different levels of theory: ωB97X-D/Def2-SV(P) (black), CAM-B3LYP-D3/Def2-TZVP (blue), and SOS-ADC(2)/Def2-TZVP (magenta). The numbers indicate the oscillator strengths associated with each transition.

Figure S3: Ionization energy (eV) as a function of the BH–NH distance (Å). The data are grouped into four categories (Group 0–3), shown as colored markers. The pink curve represents the global exponential fit, and the shaded area corresponds to the 95% confidence interval.

Figure S4: Molecular structure of the 1BH,2H structure. The substituted sites are highlighted by red dashed outlines, and the numbers connecting the BH and NH groups indicate the corresponding distances in angstroms (Å).

Table S1: Summary of the S_1_ excited state for the 1BH,2H system structure, including the structural name, electron and hole distributions, dominant orbital contributions, charge transfer (C.T.) character, excitation energy (E, in eV), and oscillator strength.

| Structural Name | Electron | Hole | Orbital | C.T. | E (eV) | Oscillator strength |
| --- | --- | --- | --- | --- | --- | --- |
| 1BH, 2H - Group 0 |  |  | 99%  (π→π*) | 0.380 | 3.85 | 0.503 |

Figure S5: Molecular structure of the 1BH, 3H system. The substituted sites are highlighted by red dashed outlines, and the numbers connecting the BH and NH groups indicate the corresponding distances in angstroms (Å).

Table S2: Summary of the S_1_ excited state for the 1BH, 3H system structure, including the structural name, electron and hole distributions, dominant orbital contributions, charge transfer (C.T.) character, excitation energy (E, in eV), and oscillator strength.

| Structural Name | Electron | Hole | Orbital | C.T | E (eV) | Oscillator strength |
| --- | --- | --- | --- | --- | --- | --- |
| 1BH, 3H - Group 0 |  |  | 99%  (π→π*) | 0.399 | 3.96 | 0.649 |

Figure S6: Molecular structure of the 3BH, 2H system. The substituted sites are highlighted by red dashed outlines, and the numbers connecting the BH and NH groups indicate the corresponding distances in angstroms (Å).

Table S3: Summary of the S_1_ excited state for the 3BH, 2H system structure, including the structural name, electron and hole distributions, dominant orbital contributions, charge transfer (C.T.) character, excitation energy (E, in eV), and oscillator strength.

| Structural Name | Electron | Hole | Orbital | C.T | E (eV) | Oscillator strength |
| --- | --- | --- | --- | --- | --- | --- |
| 3BH, 2H - Group 0 |  |  | 99%  (π→π*) | 0.453 | 3.53 | 0.622 |

Figure S7: Molecular structure of the 2BH, 3H system. The substituted sites are highlighted by red dashed outlines, and the numbers connecting the BH and NH groups indicate the corresponding distances in angstroms (Å).

Table S4: Summary of the S_1_ excited state for the 2BH, 3H system structure, including the structural name, electron and hole distributions, dominant orbital contributions, charge transfer (C.T.) character, excitation energy (E, in eV), and oscillator strength.

| Structural Name | Electron | Hole | Orbital | C.T | E (eV) | Oscillator strength |
| --- | --- | --- | --- | --- | --- | --- |
| 2BH, 3H - Group 0 |  |  | 99%  (π→π*) | 0.393 | 4.01 | 0.405 |

Figure S8: Molecular structure of the 3BH, 1H system. The substituted sites are highlighted by red dashed outlines, and the numbers connecting the BH and NH groups indicate the corresponding distances in angstroms (Å).

Table S5: Summary of the S_1_ excited state for the 3BH, 1H system structure, including the structural name, electron and hole distributions, dominant orbital contributions, charge transfer (C.T.) character, excitation energy (E, in eV), and oscillator strength.

| Structural Name | Electron | Hole | Orbital | C.T | E (eV) | Oscillator strength |
| --- | --- | --- | --- | --- | --- | --- |
| 3BH, 1H - Group 0 |  |  | 99%  (π→π*) | 0.384 | 3.75 | 0.613 |

Figure S9: Molecular structure of the 2BH, 1H system. The substituted sites are highlighted by red dashed outlines, and the numbers connecting the BH and NH groups indicate the corresponding distances in angstroms (Å).

Table S6: Summary of the S_1_ excited state for the 2BH, 1H system structure, including the structural name, electron and hole distributions, dominant orbital contributions, charge transfer (C.T.) character, excitation energy (E, in eV), and oscillator strength.

| Structural Name | Electron | Hole | Orbital | C.T | E (eV) | Oscillator strength |
| --- | --- | --- | --- | --- | --- | --- |
| 2BH, 1H - Group 0 |  |  | 99%  (π→π*) | 0.283 | 3.95 | 0.492 |

Figure S10: Molecular structure of the 1BH, 5H system. The substituted sites are highlighted by red dashed outlines, and the numbers connecting the BH and NH groups indicate the corresponding distances in angstroms (Å).

Table S7: Summary of the S_1_ excited state for the 1BH, 5H system structure, including the structural name, electron and hole distributions, dominant orbital contributions, charge transfer (C.T.) character, excitation energy (E, in eV), and oscillator strength.

| Structural Name | Electron | Hole | Orbital | C.T | E (eV) | Oscillator strength |
| --- | --- | --- | --- | --- | --- | --- |
| 1BH, 5H - Group 1 |  |  | 99%  (π→π*) | 0.696 | 2.54 | 0.521 |

Figure S11: Molecular structure of the 1BH, 16H system. The substituted sites are highlighted by red dashed outlines, and the numbers connecting the BH and NH groups indicate the corresponding distances in angstroms (Å).

Table S8: Summary of the S_1_ excited state for the 1BH, 16H system structure, including the structural name, electron and hole distributions, dominant orbital contributions, charge transfer (C.T.) character, excitation energy (E, in eV), and oscillator strength.

| Structural Name | Electron | Hole | Orbital | C.T | E (eV) | Oscillator strength |
| --- | --- | --- | --- | --- | --- | --- |
| 1BH, 16H - Group 1 |  |  | 99%  (π→π*) | 0.815 | 2.58 | 0.431 |

Figure S12: Molecular structure of the 1BH, 17H system. The substituted sites are highlighted by red dashed outlines, and the numbers connecting the BH and NH groups indicate the corresponding distances in angstroms (Å).

Table S9: Summary of the S_1_ excited state for the 1BH, 17H system structure, including the structural name, electron and hole distributions, dominant orbital contributions, charge transfer (C.T.) character, excitation energy (E, in eV), and oscillator strength.

| Structural Name | Electron | Hole | Orbital | C.T | E (eV) | Oscillator strength |
| --- | --- | --- | --- | --- | --- | --- |
| 1BH, 17H - Group 1 |  |  | 99%  (π→π*) | 0.828 | 2.61 | 0.332 |

Figure S13: Molecular structure of the 1BH, 6H system. The substituted sites are highlighted by red dashed outlines, and the numbers connecting the BH and NH groups indicate the corresponding distances in angstroms (Å).

Table S10: Summary of the S_1_ excited state for the 1BH, 6H system structure, including the structural name, electron and hole distributions, dominant orbital contributions, charge transfer (C.T.) character, excitation energy (E, in eV), and oscillator strength.

| Structural Name | Electron | Hole | Orbital | C.T | E (eV) | Oscillator strength |
| --- | --- | --- | --- | --- | --- | --- |
| 1BH, 6H - Group 1 |  |  | 99%  (π→π*) | 0.930 | 2.70 | 0.019 |

Figure S14: Molecular structure of the 1BH, 18H system. The substituted sites are highlighted by red dashed outlines, and the numbers connecting the BH and NH groups indicate the corresponding distances in angstroms (Å).

Table S11: Summary of the S_1_ excited state for the 1BH, 18H system structure, including the structural name, electron and hole distributions, dominant orbital contributions, charge transfer (C.T.) character, excitation energy (E, in eV), and oscillator strength.

| Structural Name | Electron | Hole | Orbital | C.T | E (eV) | Oscillator strength |
| --- | --- | --- | --- | --- | --- | --- |
| 1BH, 18H - Group 1 |  |  | 99%  (π→π*) | 0.828 | 3.46 | 0.022 |

Figure S15: Molecular structure of the 3BH, 16H system. The substituted sites are highlighted by red dashed outlines, and the numbers connecting the BH and NH groups indicate the corresponding distances in angstroms (Å).

Table S12: Summary of the S_1_ excited state for the 3BH, 16H system structure, including the structural name, electron and hole distributions, dominant orbital contributions, charge transfer (C.T.) character, excitation energy (E, in eV), and oscillator strength.

| Structural Name | Electron | Hole | Orbital | C.T | E (eV) | Oscillator strength |
| --- | --- | --- | --- | --- | --- | --- |
| 3BH, 16H - Group 1 |  |  | 99%  (π→π*) | 0.767 | 2.26 | 0.240 |

Figure S16: Molecular structure of the 2BH, 4H system. The substituted sites are highlighted by red dashed outlines, and the numbers connecting the BH and NH groups indicate the corresponding distances in angstroms (Å).

Table S13: Summary of the S_1_ excited state for the 2BH, 4H system structure, including the structural name, electron and hole distributions, dominant orbital contributions, charge transfer (C.T.) character, excitation energy (E, in eV), and oscillator strength.

| Structural Name | Electron | Hole | Orbital | C.T | E (eV) | Oscillator strength |
| --- | --- | --- | --- | --- | --- | --- |
| 2BH, 4H - Group 1 |  |  | 99%  (π→π*) | 0.732 | 3.20 | 0.284 |

Figure S17: Molecular structure of the 2BH, 6H system. The substituted sites are highlighted by red dashed outlines, and the numbers connecting the BH and NH groups indicate the corresponding distances in angstroms (Å).

Table S14: Summary of the S_1_ excited state for the 2BH, 6H system structure, including the structural name, electron and hole distributions, dominant orbital contributions, charge transfer (C.T.) character, excitation energy (E, in eV), and oscillator strength.

| Structural Name | Electron | Hole | Orbital | C.T | E (eV) | Oscillator strength |
| --- | --- | --- | --- | --- | --- | --- |
| 2BH, 6H - Group 1 |  |  | 99%  (π→π*) | 0.892 | 2.73 | 0.017 |

Figure S18: Molecular structure of the 2BH, 16H system. The substituted sites are highlighted by red dashed outlines, and the numbers connecting the BH and NH groups indicate the corresponding distances in angstroms (Å).

Table S15: Summary of the S_1_ excited state for the 2BH, 16H system structure, including the structural name, electron and hole distributions, dominant orbital contributions, charge transfer (C.T.) character, excitation energy (E, in eV), and oscillator strength.

| Structural Name | Electron | Hole | Orbital | C.T | E (eV) | Oscillator strength |
| --- | --- | --- | --- | --- | --- | --- |
| 2BH, 16H - Group 1 |  |  | 99%  (π→π*) | 0.847 | 2.33 | 0.312 |

Figure S19: Molecular structure of the 2BH, 17H system. The substituted sites are highlighted by red dashed outlines, and the numbers connecting the BH and NH groups indicate the corresponding distances in angstroms (Å).

Table S16: Summary of the S_1_ excited state for the 2BH, 17H system structure, including the structural name, electron and hole distributions, dominant orbital contributions, charge transfer (C.T.) character, excitation energy (E, in eV), and oscillator strength.

| Structural Name | Electron | Hole | Orbital | C.T | E (eV) | Oscillator strength |
| --- | --- | --- | --- | --- | --- | --- |
| 2BH, 17H - Group 1 |  |  | 99%  (π→π*) | 0.859 | 2.37 | 0.347 |

Figure S20: Molecular structure of the 3BH, 5H system. The substituted sites are highlighted by red dashed outlines, and the numbers connecting the BH and NH groups indicate the corresponding distances in angstroms (Å).

Table S17: Summary of the S_1_ excited state for the 3BH, 5H system structure, including the structural name, electron and hole distributions, dominant orbital contributions, charge transfer (C.T.) character, excitation energy (E, in eV), and oscillator strength.

| Structural Name | Electron | Hole | Orbital | C.T | E (eV) | Oscillator strength |
| --- | --- | --- | --- | --- | --- | --- |
| 3BH, 5H - Group 1 |  |  | 99%  (π→π*) | 0.773 | 2.54 | 0.609 |

Figure S21: Molecular structure of the 3BH, 6H system. The substituted sites are highlighted by red dashed outlines, and the numbers connecting the BH and NH groups indicate the corresponding distances in angstroms (Å).

Table S18: Summary of the S_1_ excited state for the 3BH, 6H system structure, including the structural name, electron and hole distributions, dominant orbital contributions, charge transfer (C.T.) character, excitation energy (E, in eV), and oscillator strength.

| Structural Name | Electron | Hole | Orbital | C.T | E (eV) | Oscillator strength |
| --- | --- | --- | --- | --- | --- | --- |
| 3BH, 6H - Group 1 |  |  | 99%  (π→π*) | 0.882 | 2.79 | 0.062 |

Figure S22: Molecular structure of the 3BH, 17H system. The substituted sites are highlighted by red dashed outlines, and the numbers connecting the BH and NH groups indicate the corresponding distances in angstroms (Å).

Table S19: Summary of the S_1_ excited state for the 3BH, 17H system structure, including the structural name, electron and hole distributions, dominant orbital contributions, charge transfer (C.T.) character, excitation energy (E, in eV), and oscillator strength.

| Structural Name | Electron | Hole | Orbital | C.T | E (eV) | Oscillator strength |
| --- | --- | --- | --- | --- | --- | --- |
| 3BH, 17H - Group 1 |  |  | 99%  (π→π*) | 0.846 | 2.30 | 0.167 |

Figure S23: Molecular structure of the 3BH, 18H system. The substituted sites are highlighted by red dashed outlines, and the numbers connecting the BH and NH groups indicate the corresponding distances in angstroms (Å).

Table S20: Summary of the S_1_ excited state for the 3BH, 18H system structure, including the structural name, electron and hole distributions, dominant orbital contributions, charge transfer (C.T.) character, excitation energy (E, in eV), and oscillator strength.

| Structural Name | Electron | Hole | Orbital | C.T | E (eV) | Oscillator strength |
| --- | --- | --- | --- | --- | --- | --- |
| 3BH, 18H - Group 1 |  |  | 99%  (π→π*) | 0.726 | 3.24 | 0.286 |

Figure S24: Molecular structure of the 2BH, 5H system. The substituted sites are highlighted by red dashed outlines, and the numbers connecting the BH and NH groups indicate the corresponding distances in angstroms (Å).

Table S21: Summary of the S_1_ excited state for the 2BH, 5H system structure, including the structural name, electron and hole distributions, dominant orbital contributions, charge transfer (C.T.) character, excitation energy (E, in eV), and oscillator strength.

| Structural Name | Electron | Hole | Orbital | C.T | E (eV) | Oscillator strength |
| --- | --- | --- | --- | --- | --- | --- |
| 2BH, 5H - Group 1 |  |  | 99%  (π→π*) | 0.748 | 2.62 | 0.416 |

Figure S25: Molecular structure of the 2BH, 18H system. The substituted sites are highlighted by red dashed outlines, and the numbers connecting the BH and NH groups indicate the corresponding distances in angstroms (Å).

Table S22: Summary of the S_1_ excited state for the 2BH, 18H system structure, including the structural name, electron and hole distributions, dominant orbital contributions, charge transfer (C.T.) character, excitation energy (E, in eV), and oscillator strength.

| Structural Name | Electron | Hole | Orbital | C.T | E (eV) | Oscillator strength |
| --- | --- | --- | --- | --- | --- | --- |
| 2BH, 18H- Group 1 |  |  | 99%  (π→π*) | 0.911 | 3.33 | 0.005 |

Figure S26: Molecular structure of the 1BH, 7H system. The substituted sites are highlighted by red dashed outlines, and the numbers connecting the BH and NH groups indicate the corresponding distances in angstroms (Å).

Table S23: Summary of the S_1_ excited state for the 1BH, 7H system structure, including the structural name, electron and hole distributions, dominant orbital contributions, charge transfer (C.T.) character, excitation energy (E, in eV), and oscillator strength.

| Structural Name | Electron | Hole | Orbital | C.T | E (eV) | Oscillator strength |
| --- | --- | --- | --- | --- | --- | --- |
| 1BH, 7H – Group 2 |  |  | 99%  (π→π*) | 0.987 | 1.61 | 0.009 |

Figure S27: Molecular structure of the 1BH, 13H system. The substituted sites are highlighted by red dashed outlines, and the numbers connecting the BH and NH groups indicate the corresponding distances in angstroms (Å).

Table S24: Summary of the S_1_ excited state for the 1BH, 13H system structure, including the structural name, electron and hole distributions, dominant orbital contributions, charge transfer (C.T.) character, excitation energy (E, in eV), and oscillator strength.

| Structural Name | Electron | Hole | Orbital | C.T | E (eV) | Oscillator strength |
| --- | --- | --- | --- | --- | --- | --- |
| 1BH, 13H – Group 2 |  |  | 99%  (π→π*) | 0.966 | 1.64 | 0.048 |

Figure S28: Molecular structure of the 1BH, 8H system. The substituted sites are highlighted by red dashed outlines, and the numbers connecting the BH and NH groups indicate the corresponding distances in angstroms (Å).

Table S25: Summary of the S_1_ excited state for the 1BH, 8H system structure, including the structural name, electron and hole distributions, dominant orbital contributions, charge transfer (C.T.) character, excitation energy (E, in eV), and oscillator strength.

| Structural Name | Electron | Hole | Orbital | C.T | E (eV) | Oscillator strength |
| --- | --- | --- | --- | --- | --- | --- |
| 1BH, 8H – Group 2 |  |  | 99%  (π→π*) | 0.984 | 1.61 | 0.012 |

Figure S29: Molecular structure of the 1BH, 14H system. The substituted sites are highlighted by red dashed outlines, and the numbers connecting the BH and NH groups indicate the corresponding distances in angstroms (Å).

Table S26: Summary of the S_1_ excited state for the 1BH, 14H system structure, including the structural name, electron and hole distributions, dominant orbital contributions, charge transfer (C.T.) character, excitation energy (E, in eV), and oscillator strength.

| Structural Name | Electron | Hole | Orbital | C.T | E (eV) | Oscillator strength |
| --- | --- | --- | --- | --- | --- | --- |
| 1BH, 14H – Group 2 |  |  | 99%  (π→π*) | 0.939 | 2.15 | 0.100 |

Figure S30: Molecular structure of the 1BH, 9H system. The substituted sites are highlighted by red dashed outlines, and the numbers connecting the BH and NH groups indicate the corresponding distances in angstroms (Å).

Table S27: Summary of the S_1_ excited state for the 1BH, 9H system structure, including the structural name, electron and hole distributions, dominant orbital contributions, charge transfer (C.T.) character, excitation energy (E, in eV), and oscillator strength.

| Structural Name | Electron | Hole | Orbital | C.T | E (eV) | Oscillator strength |
| --- | --- | --- | --- | --- | --- | --- |
| 1BH, 9H – Group 2 |  |  | 99%  (π→π*) | 0.963 | 2.45 | 0.026 |

Figure S31: Molecular structure of the 1BH, 15H system. The substituted sites are highlighted by red dashed outlines, and the numbers connecting the BH and NH groups indicate the corresponding distances in angstroms (Å).

Table S28: Summary of the S_1_ excited state for the 1BH, 15H system structure, including the structural name, electron and hole distributions, dominant orbital contributions, charge transfer (C.T.) character, excitation energy (E, in eV), and oscillator strength.

| Structural Name | Electron | Hole | Orbital | C.T | E (eV) | Oscillator strength |
| --- | --- | --- | --- | --- | --- | --- |
| 1BH, 15H – Group 2 |  |  | 99%  (π→π*) | 0.996 | 2.29 | 0.511 |

Figure S32: Molecular structure of the 3BH, 14H system. The substituted sites are highlighted by red dashed outlines, and the numbers connecting the BH and NH groups indicate the corresponding distances in angstroms (Å).

Table S29: Summary of the S_1_ excited state for the 3BH, 14H system structure, including the structural name, electron and hole distributions, dominant orbital contributions, charge transfer (C.T.) character, excitation energy (E, in eV), and oscillator strength.

| Structural Name | Electron | Hole | Orbital | C.T | E (eV) | Oscillator strength |
| --- | --- | --- | --- | --- | --- | --- |
| 3BH, 14H – Group 2 |  |  | 99%  (π→π*) | 0.949 | 1.92 | 0.048 |

Figure S33: Molecular structure of the 3BH, 8H system. The substituted sites are highlighted by red dashed outlines, and the numbers connecting the BH and NH groups indicate the corresponding distances in angstroms (Å).

Table S30: Summary of the S_1_ excited state for the 3BH, 8H system structure, including the structural name, electron and hole distributions, dominant orbital contributions, charge transfer (C.T.) character, excitation energy (E, in eV), and oscillator strength.

| Structural Name | Electron | Hole | Orbital | C.T | E (eV) | Oscillator strength |
| --- | --- | --- | --- | --- | --- | --- |
| 3BH, 8H – Group 2 |  |  | 99%  (π→π*) | 0.982 | 1.58 | 0.012 |

Figure S34: Molecular structure of the 2BH, 7H system. The substituted sites are highlighted by red dashed outlines, and the numbers connecting the BH and NH groups indicate the corresponding distances in angstroms (Å).

Table S31: Summary of the S_1_ excited state for the 2BH, 7H system structure, including the structural name, electron and hole distributions, dominant orbital contributions, charge transfer (C.T.) character, excitation energy (E, in eV), and oscillator strength.

| Structural Name | Electron | Hole | Orbital | C.T | E (eV) | Oscillator strength |
| --- | --- | --- | --- | --- | --- | --- |
| 2BH, 7H – Group 2 |  |  | 99%  (π→π*) | 0.980 | 1.63 | 0.017 |

Figure S35: Molecular structure of the 2BH, 9H system. The substituted sites are highlighted by red dashed outlines, and the numbers connecting the BH and NH groups indicate the corresponding distances in angstroms (Å).

Table S32: Summary of the S_1_ excited state for the 2BH, 9H system structure, including the structural name, electron and hole distributions, dominant orbital contributions, charge transfer (C.T.) character, excitation energy (E, in eV), and oscillator strength.

| Structural Name | Electron | Hole | Orbital | C.T | E (eV) | Oscillator strength |
| --- | --- | --- | --- | --- | --- | --- |
| 2BH, 9H – Group 2 |  |  | 99%  (π→π*) | 0.943 | 2.41 | 0.035 |

Figure S36: Molecular structure of the 2BH, 13H system. The substituted sites are highlighted by red dashed outlines, and the numbers connecting the BH and NH groups indicate the corresponding distances in angstroms (Å).

Table S33: Summary of the S_1_ excited state for the 2BH, 13H system structure, including the structural name, electron and hole distributions, dominant orbital contributions, charge transfer (C.T.) character, excitation energy (E, in eV), and oscillator strength.

| Structural Name | Electron | Hole | Orbital | C.T | E (eV) | Oscillator strength |
| --- | --- | --- | --- | --- | --- | --- |
| 2BH, 13H – Group 2 |  |  | 99%  (π→π*) | 0.976 | 1.53 | 0.030 |

Figure S37: Molecular structure of the 2BH, 14H system. The substituted sites are highlighted by red dashed outlines, and the numbers connecting the BH and NH groups indicate the corresponding distances in angstroms (Å).

Table S34: Summary of the S_1_ excited state for the 2BH, 14H system structure, including the structural name, electron and hole distributions, dominant orbital contributions, charge transfer (C.T.) character, excitation energy (E, in eV), and oscillator strength.

| Structural Name | Electron | Hole | Orbital | C.T | E (eV) | Oscillator strength |
| --- | --- | --- | --- | --- | --- | --- |
| 2BH, 14H – Group 2 |  |  | 99%  (π→π*) | 0.957 | 1.96 | 0.058 |

Figure S38: Molecular structure of the 2BH, 15H system. The substituted sites are highlighted by red dashed outlines, and the numbers connecting the BH and NH groups indicate the corresponding distances in angstroms (Å).

Table S35: Summary of the S_1_ excited state for the 2BH, 15H system structure, including the structural name, electron and hole distributions, dominant orbital contributions, charge transfer (C.T.) character, excitation energy (E, in eV), and oscillator strength.

| Structural Name | Electron | Hole | Orbital | C.T | E (eV) | Oscillator strength |
| --- | --- | --- | --- | --- | --- | --- |
| 2BH, 15H – Group 2 |  |  | 99%  (π→π*) | 0.990 | 2.13 | 0.000 |

Figure S39: Molecular structure of the 3BH, 7H system. The substituted sites are highlighted by red dashed outlines, and the numbers connecting the BH and NH groups indicate the corresponding distances in angstroms (Å).

Table S36: Summary of the S_1_ excited state for the 3BH, 7H system structure, including the structural name, electron and hole distributions, dominant orbital contributions, charge transfer (C.T.) character, excitation energy (E, in eV), and oscillator strength.

| Structural Name | Electron | Hole | Orbital | C.T | E (eV) | Oscillator strength |
| --- | --- | --- | --- | --- | --- | --- |
| 3BH, 7H – Group 2 |  |  | 99%  (π→π*) | 0.984 | 1.52 | 0.010 |

Figure S40: Molecular structure of the 3BH, 9H system. The substituted sites are highlighted by red dashed outlines, and the numbers connecting the BH and NH groups indicate the corresponding distances in angstroms (Å).

Table S37: Summary of the S_1_ excited state for the 3BH, 9H system structure, including the structural name, electron and hole distributions, dominant orbital contributions, charge transfer (C.T.) character, excitation energy (E, in eV), and oscillator strength.

| Structural Name | Electron | Hole | Orbital | C.T | E (eV) | Oscillator strength |
| --- | --- | --- | --- | --- | --- | --- |
| 3BH, 9H – Group 2 |  |  | 99%  (π→π*) | 0.947 | 2.42 | 0.036 |

Figure S41: Molecular structure of the 3BH, 13H system. The substituted sites are highlighted by red dashed outlines, and the numbers connecting the BH and NH groups indicate the corresponding distances in angstroms (Å).

Table S38: Summary of the S_1_ excited state for the 3BH, 13H system structure, including the structural name, electron and hole distributions, dominant orbital contributions, charge transfer (C.T.) character, excitation energy (E, in eV), and oscillator strength.

| Structural Name | Electron | Hole | Orbital | C.T | E (eV) | Oscillator strength |
| --- | --- | --- | --- | --- | --- | --- |
| 3BH, 13H – Group 2 |  |  | 99%  (π→π*) | 0.973 | 1.46 | 0.019 |

Figure S42: Molecular structure of the 3BH, 15H system. The substituted sites are highlighted by red dashed outlines, and the numbers connecting the BH and NH groups indicate the corresponding distances in angstroms (Å).

Table S39: Summary of the S_1_ excited state for the 3BH, 15H system structure, including the structural name, electron and hole distributions, dominant orbital contributions, charge transfer (C.T.) character, excitation energy (E, in eV), and oscillator strength.

| Structural Name | Electron | Hole | Orbital | C.T | E (eV) | Oscillator strength |
| --- | --- | --- | --- | --- | --- | --- |
| 3BH, 15H – Group 2 |  |  | 99%  (π→π*) | 0.966 | 2.11 | 0.020 |

Figure S43: Molecular structure of the 2BH, 8H system. The substituted sites are highlighted by red dashed outlines, and the numbers connecting the BH and NH groups indicate the corresponding distances in angstroms (Å).

Table S40: Summary of the S_1_ excited state for the 2BH, 8H system structure, including the structural name, electron and hole distributions, dominant orbital contributions, charge transfer (C.T.) character, excitation energy (E, in eV), and oscillator strength.

| Structural Name | Electron | Hole | Orbital | C.T | E (eV) | Oscillator strength |
| --- | --- | --- | --- | --- | --- | --- |
| 2BH, 8H – Group 2 |  |  | 99%  (π→π*) | 0.975 | 1.62 | 0.023 |

Figure S44: Molecular structure of the 2BH, 12H system. The substituted sites are highlighted by red dashed outlines, and the numbers connecting the BH and NH groups indicate the corresponding distances in angstroms (Å).

Table S41: Summary of the S_1_ excited state for the 2BH, 12H system structure, including the structural name, electron and hole distributions, dominant orbital contributions, charge transfer (C.T.) character, excitation energy (E, in eV), and oscillator strength.

| Structural Name | Electron | Hole | Orbital | C.T | E (eV) | Oscillator strength |
| --- | --- | --- | --- | --- | --- | --- |
| 2BH, 12H – Group 3 |  |  | 99%  (π→π*) | 0.998 | 2.06 | 0.001 |

Figure S45: Molecular structure of the 1BH, 10H system. The substituted sites are highlighted by red dashed outlines, and the numbers connecting the BH and NH groups indicate the corresponding distances in angstroms (Å).

Table S42: Summary of the S_1_ excited state for the 1BH, 10H system structure, including the structural name, electron and hole distributions, dominant orbital contributions, charge transfer (C.T.) character, excitation energy (E, in eV), and oscillator strength.

| Structural Name | Electron | Hole | Orbital | C.T | E (eV) | Oscillator strength |
| --- | --- | --- | --- | --- | --- | --- |
| 1BH, 10H – Group 3 |  |  | 99%  (π→π*) | 0.998 | 1.58 | 0.001 |

Figure S46: Molecular structure of the 1BH, 11H system. The substituted sites are highlighted by red dashed outlines, and the numbers connecting the BH and NH groups indicate the corresponding distances in angstroms (Å).

Table S43: Summary of the S_1_ excited state for the 1BH, 11H system structure, including the structural name, electron and hole distributions, dominant orbital contributions, charge transfer (C.T.) character, excitation energy (E, in eV), and oscillator strength.

| Structural Name | Electron | Hole | Orbital | C.T | E (eV) | Oscillator strength |
| --- | --- | --- | --- | --- | --- | --- |
| 1BH, 11H – Group 3 |  |  | 99%  (π→π*) | 0.999 | 1.45 | 0.000 |

Figure S47: Molecular structure of the 1BH, 12H system. The substituted sites are highlighted by red dashed outlines, and the numbers connecting the BH and NH groups indicate the corresponding distances in angstroms (Å).

Table S44: Summary of the S_1_ excited state for the 1BH, 12H system structure, including the structural name, electron and hole distributions, dominant orbital contributions, charge transfer (C.T.) character, excitation energy (E, in eV), and oscillator strength.

| Structural Name | Electron | Hole | Orbital | C.T | E (eV) | Oscillator strength |
| --- | --- | --- | --- | --- | --- | --- |
| 1BH, 12H – Group 3 |  |  | 99%  (π→π*) | 0.996 | 2.11 | 0.002 |

Figure S48: Molecular structure of the 3BH, 10H system. The substituted sites are highlighted by red dashed outlines, and the numbers connecting the BH and NH groups indicate the corresponding distances in angstroms (Å).

Table S45: Summary of the S_1_ excited state for the 3BH, 10H system structure, including the structural name, electron and hole distributions, dominant orbital contributions, charge transfer (C.T.) character, excitation energy (E, in eV), and oscillator strength.

| Structural Name | Electron | Hole | Orbital | C.T | E (eV) | Oscillator strength |
| --- | --- | --- | --- | --- | --- | --- |
| 3BH, 10H – Group 3 |  |  | 99%  (π→π*) | 0.998 | 1.53 | 0.002 |

Figure S49: Molecular structure of the 2BH, 10H system. The substituted sites are highlighted by red dashed outlines, and the numbers connecting the BH and NH groups indicate the corresponding distances in angstroms (Å).

Table S46: Summary of the S_1_ excited state for the 2BH, 10H system structure, including the structural name, electron and hole distributions, dominant orbital contributions, charge transfer (C.T.) character, excitation energy (E, in eV), and oscillator strength.

| Structural Name | Electron | Hole | Orbital | C.T | E (eV) | Oscillator strength |
| --- | --- | --- | --- | --- | --- | --- |
| 2BH, 10H – Group 3 |  |  | 99%  (π→π*) | 0.997 | 1.58 | 0.001 |

Figure S50: Molecular structure of the 2BH, 12H system. The substituted sites are highlighted by red dashed outlines, and the numbers connecting the BH and NH groups indicate the corresponding distances in angstroms (Å).

Table S47: Summary of the S_1_ excited state for the 2BH, 12H system structure, including the structural name, electron and hole distributions, dominant orbital contributions, charge transfer (C.T.) character, excitation energy (E, in eV), and oscillator strength.

| Structural Name | Electron | Hole | Orbital | C.T | E (eV) | Oscillator strength |
| --- | --- | --- | --- | --- | --- | --- |
| 2BH, 12H – Group 3 |  |  | 99%  (π→π*) | 0.997 | 2.06 | 0.001 |

Figure S51: Molecular structure of the 2BH, 11H system. The substituted sites are highlighted by red dashed outlines, and the numbers connecting the BH and NH groups indicate the corresponding distances in angstroms (Å).

Table S48: Summary of the S_1_ excited state for the 2BH, 11H system structure, including the structural name, electron and hole distributions, dominant orbital contributions, charge transfer (C.T.) character, excitation energy (E, in eV), and oscillator strength.

| Structural Name | Electron | Hole | Orbital | C.T | E (eV) | Oscillator strength |
| --- | --- | --- | --- | --- | --- | --- |
| 2BH, 11H – Group 3 |  |  | 99%  (π→π*) | 0.999 | 1.39 | 0.000 |

Figure S52: Molecular structure of the 3BH, 11H system. The substituted sites are highlighted by red dashed outlines, and the numbers connecting the BH and NH groups indicate the corresponding distances in angstroms (Å).

Table S49: Summary of the S_1_ excited state for the 3BH, 11H system structure, including the structural name, electron and hole distributions, dominant orbital contributions, charge transfer (C.T.) character, excitation energy (E, in eV), and oscillator strength.

| Structural Name | Electron | Hole | Orbital | C.T | E (eV) | Oscillator strength |
| --- | --- | --- | --- | --- | --- | --- |
| 3BH, 11H – Group 3 |  |  | 99%  (π→π*) | 0.999 | 1.33 | 0.003 |

Figure S53: Molecular structure of the 3BH, 12H system. The substituted sites are highlighted by red dashed outlines, and the numbers connecting the BH and NH groups indicate the corresponding distances in angstroms (Å).

Table S50: Summary of the S_1_ excited state for the 3BH, 12H system structure, including the structural name, electron and hole distributions, dominant orbital contributions, charge transfer (C.T.) character, excitation energy (E, in eV), and oscillator strength.

| Structural Name | Electron | Hole | Orbital | C.T | E (eV) | Oscillator strength |
| --- | --- | --- | --- | --- | --- | --- |
| 3BH, 12H – Group 3 |  |  | 99%  (π→π*) | 0.998 | 2.03 | 0.000 |

Table S 51: Vertical excitation energy and oscillator strength (f) of the lowest singlet state (S_1_) and of the bright state (S_0_→S_n_, lowest state with significant f).

| Structure | E(S_1_) / eV | f(S_1_) | | Bright state | E(bright) | F (bright) |
| --- | --- | --- | --- | --- | --- | --- |
| Group 0 – same ring (S_1_ is the bright state) | | | | | | |
| PTI-1BH,2H | 3.85 | 0.503 | S_1_ | | - | - |
| PTI-1BH,3H | 3.96 | 0.649 | S_1_ | | - | - |
| PTI-3BH,2H | 3.53 | 0.622 | S_1_ | | - | - |
| PTI-2BH,3H | 4.01 | 0.405 | S_1_ | | - | - |
| PTI-3BH,1H | 3.75 | 0.613 | S_1_ | | - | - |
| PTI-2BH,1H | 3.95 | 0.492 | S_1_ | | - | - |
| Group 1 – one intervening ring | | | | | | |
| PTI-1BH,5H | 2.54 | 0.521 | S_1_ | | - | - |
| PTI-1BH,16H | 2.58 | 0.431 | S_1_ | | - | - |
| PTI-1BH,17H | 2.61 | 0.332 | S_1_ | | - | - |
| PTI-1BH,6H | 2.70 | 0.019 | S_4_ | | 4.10 | 0.424 |
| PTI-1BH,18H | 3.46 | 0.022 | S_2_ | | 3.61 | 0.558 |
| PTI-3BH,16H | 2.26 | 0.240 | S_1_ | | - | - |
| PTI-2BH,4H | 3.20 | 0.287 | S_1_ | | - | - |
| PTI-2BH,6H | 2.73 | 0.017 | S_2_ | | 3.08 | 0.281 |
| PTI-2BH,16H | 2.33 | 0.312 | S_1_ | | - | - |
| PTI-2BH,17H | 2.37 | 0.347 | S_1_ | | - | - |
| PTI-3BH,5H | 2.54 | 0.609 | S_1_ | | - | - |
| PTI-3BH,6H | 2.79 | 0.062 | S_2_ | | 2.99 | 0.499 |
| PTI-3BH,17H | 2.30 | 0.167 | S_1_ | | - | - |
| PTI-3BH,18H | 3.24 | 0.286 | S_1_ | | - | - |
| PTI-2BH,5H | 2.62 | 0.416 | S_1_ | | - | - |
| PTI-2BH,18H | 3.33 | 0.005 | S_2_ | | 3.43 | 0.332 |
| Group 2 – two intervening rings | | | | | | |
| PTI-1BH,7H | 1.61 | 0.009 | S_4_ | | 3.55 | 0.218 |
| PTI-1BH,13H | 1.64 | 0.048 | S_5_ | | 3.72 | 0.556 |
| PTI-1BH,8H | 1.61 | 0.012 | S_5_ | | 3.63 | 0.340 |
| PTI-1BH,14H | 2.15 | 0.100 | S_3_ | | 3.52 | 0.405 |
| PTI-1BH,9H | 2.45 | 0.026 | S_3_ | | 3.48 | 0.308 |
| PTI-1BH,15H | 2.29 | 0.511 | S_1_ | | - | - |
| PTI-3BH,14H | 1.92 | 0.048 | S_3_ | | 3.19 | 0.275 |
| PTI-3BH,8H | 1.58 | 0.012 | S_4_ | | 3.35 | 0.450 |
| PTI-2BH,7H | 1.63 | 0.017 | S_3_ | | 3.53 | 0.323 |
| PTI-2BH,9H | 2.41 | 0.035 | S_3_ | | 3.38 | 0.270 |
| PTI-2BH,13H | 1.53 | 0.030 | S_5_ | | 3.67 | 0.393 |
| PTI-2BH,14H | 1.96 | 0.058 | S_3_ | | 3.42 | 0.332 |
| PTI-2BH,15H | 2.13 | 0.000 | S_4_ | | 3.67 | 0.365 |
| PTI-3BH,7H | 1.52 | 0.010 | S_3_ | | 3.28 | 0.487 |
| PTI-3BH,9H | 2.42 | 0.036 | S_3_ | | 3.23 | 0.530 |
| PTI-3BH,13H | 1.46 | 0.019 | S_3_ | | 3.31 | 0.282 |
| PTI-3BH,15H | 2.11 | 0.500 | S_1_ | | - | - |
| PTI-2BH,8H | 1.62 | 0.023 | S_4_ | | 3.63 | 0.360 |
| PTI-2BH,12H | 2.06 | 0.001 | S_5_ | | 3.92 | 0.194 |
| Group 3 - three intervening rings (S_1_ is a dark CT state) | | | | | | |
| PTI-1BH,10H | 1.58 | 0.001 | S_4_ | | 3.36 | 0.172 |
| PTI-1BH,11H | 1.45 | 0.000 | S_3_ | | 3.16 | 0.122 |
| PTI-1BH,12H | 2.11 | 0.002 | S_5_ | | 3.95 | 0.480 |
| PTI-3BH,10H | 1.53 | 0.002 | S_5_ | | 3.58 | 0.311 |
| PTI-2BH,10H | 1.58 | 0.001 | S₇ | | 3.82 | 0.168 |
| PTI-2BH,12H | 2.06 | 0.001 | S_5_ | | 3.92 | 0.198 |
| PTI-2BH,11H | 1.39 | 0.000 | S_8_ | | 3.87 | 0.248 |
| PTI-3BH,11H | 1.33 | 0.003 | S_4_ | | 3.28 | 0.197 |
| PTI-3BH,12H | 2.03 | 0.000 | S_4_ | | 3.36 | 0.267 |

Figure S54: Thermodynamic criteria for PTI Group 0 structures. The blue level represents the ionization energy, the orange level corresponds to the first excited-state energy (S_1_), and the red level indicates the excited state with the highest oscillator strength (bright state), with the corresponding oscillator strength values shown above the red level.

Figure S55: Thermodynamic criteria for PTI Group 1 structures. The blue level represents the ionization energy, the orange level corresponds to the first excited-state energy (S_1_), and the red level indicates the excited state with the highest oscillator strength (bright state), with the corresponding oscillator strength values shown above the red level.

Figure S56: Thermodynamic criteria for PTI Group 2 structures. The blue level represents the ionization energy, the orange level corresponds to the first excited-state energy (S_1_), and the red level indicates the excited state with the highest oscillator strength (bright state), with the corresponding oscillator strength values shown above the red level.

Figure S57: Thermodynamic criteria for PTI Group 3 structures. The blue level represents the ionization energy, the orange level corresponds to the first excited-state energy (S_1_), and the red level indicates the excited state with the highest oscillator strength (bright state), with the corresponding oscillator strength values shown above the red level.

Figure S58: Representative intrinsic bond orbitals (IBOs) highlighting the π-framework in PTI and boron-substituted structures. The orbitals illustrate the delocalization of π electron density toward adjacent carbon atoms upon nitrogen-to-boron substitution, as evidenced by the expansion of the orbital lobes near the substitution sites.

PTI-

C -4.63673 1.55396 -0.00257

N -5.49684 2.57773 -0.00429

C -4.91737 3.77958 -0.00414

C -2.86012 2.91304 -0.00089

N -3.31833 1.65233 -0.00084

H -6.72520 4.73847 -0.00732

N -3.59650 4.00894 -0.00246

N -5.26150 0.32488 -0.00260

N -5.72290 4.86268 -0.00584

N -1.48367 2.97971 0.00092

H -5.30557 5.78254 -0.00591

H -1.03137 2.07136 0.00132

H -6.27352 0.38735 -0.00327

N -6.27717 -4.12195 0.00080

H -5.97606 -5.08614 0.00209

N -4.06194 -3.53586 0.00044

C -5.34463 -3.14604 0.00018

H -7.25662 -3.87565 0.00121

N -1.83873 -2.77399 -0.00042

C -3.19654 -2.53859 -0.00034

N -5.77191 -1.88203 -0.00068

C -4.79254 -0.97163 -0.00145

N -3.49626 -1.23115 -0.00140

H -1.27808 -1.92816 -0.00204

N -1.35037 -7.38708 0.00968

H -0.74197 -8.19321 0.01056

N 0.51546 -6.04873 0.00426

C -0.81513 -6.14794 0.00620

H -2.35568 -7.48550 0.01091

C 0.97213 -4.79193 0.00089

N -1.67372 -5.11846 0.00500

C -1.09278 -3.93268 0.00144

N 0.22823 -3.69905 -0.00075

N 0.68141 3.64303 0.00193

C -0.60068 4.03766 0.00161

N 2.91180 4.39381 0.00296

C 1.55443 4.63582 0.00250

N -1.03089 5.28600 0.00182

C -0.05186 6.20171 0.00242

N 1.25634 5.93928 0.00280

H 3.47193 5.23902 0.00329

N -0.43012 7.49741 0.00279

H -1.41563 7.71895 0.00236

H 0.27312 8.22229 0.00311

N 3.32326 -0.20542 -0.00014

N 5.27060 1.11078 -0.00002

C 3.95326 1.02022 0.00033

H 2.31046 -0.14283 0.00089

N 7.07261 2.52641 0.00038

C 5.73192 2.36954 0.00069

H 7.66077 1.70518 0.00080

N 3.09024 2.04708 0.00134

C 3.66380 3.23823 0.00202

N 4.98041 3.47201 0.00176

H 7.46587 3.45664 0.00212

N 2.34898 -4.71898 -0.00103

H 2.80075 -5.62671 -0.00082

N 4.51543 -4.05867 -0.00439

C 3.23758 -3.66481 -0.00201

N 6.70807 -3.37741 -0.00763

C 5.39683 -3.05692 -0.00528

H 6.98377 -4.34901 -0.00947

N 2.81487 -2.41217 -0.00057

C 3.79766 -1.49923 -0.00158

N 5.09383 -1.75105 -0.00393

H 7.39288 -2.63485 -0.00910

1BH,2H

C 0.44757 -4.86874 0.04026

N -0.12518 -6.07468 0.04986

C -1.46113 -6.04242 0.03985

C -1.52221 -3.80973 0.01436

N -0.18346 -3.71045 0.02294

H -1.58770 -8.08467 0.06382

N -2.21500 -4.93391 0.02176

N 1.83045 -4.92049 0.05022

N -2.11409 -7.22277 0.04855

N -2.14122 -2.58181 -0.00256

H -3.12439 -7.22281 0.04272

H -1.49360 -1.80046 -0.00163

H 2.20345 -5.86329 0.06447

N 6.27774 -3.90907 0.05290

H 7.02653 -3.23600 0.13262

N 4.77900 -2.16368 0.00704

C 4.99461 -3.49012 0.04027

H 6.46549 -4.89855 0.13925

N 3.15007 -0.49625 -0.01326

C 3.48770 -1.81413 0.00560

N 4.04275 -4.41918 0.05474

C 2.79111 -3.93894 0.04079

N 2.45521 -2.66037 0.02207

H 2.15499 -0.29873 0.00633

N 7.48392 1.21551 -0.17704

H 8.10493 2.00442 -0.31828

B 5.56727 2.94262 -0.06510

C 6.14397 1.56489 -0.12745

H 7.73643 0.41579 -0.75243

C 4.05995 2.92662 0.00357

N 5.28758 0.49818 -0.09376

C 3.94729 0.65224 -0.02701

N 3.33125 1.79422 0.01764

N -3.61201 -0.86398 -0.02598

C -3.46556 -2.19592 -0.01919

N -5.18689 0.88293 -0.04930

C -4.87033 -0.45624 -0.04209

N -4.43637 -3.08978 -0.02778

C -5.66693 -2.55528 -0.04472

N -5.94639 -1.25141 -0.05247

H -6.18515 1.06075 -0.05996

N -6.70456 -3.41846 -0.05466

H -6.51607 -4.41081 -0.04878

H -7.64942 -3.06178 -0.06724

N -1.14603 3.13328 0.00434

N -3.14206 4.36981 -0.01252

C -2.51913 3.20467 -0.00970

H -0.78403 2.18596 -0.00859

N -5.16641 5.44153 -0.03273

C -4.47795 4.28012 -0.02841

H -4.65452 6.31236 -0.02245

N -3.10939 1.99658 -0.02246

C -4.42961 2.03865 -0.03669

N -5.18000 3.14518 -0.04048

H -6.17606 5.42335 -0.04377

N 3.37068 4.13774 0.04173

H 3.97026 4.95469 0.05467

N 1.86153 5.82215 0.09058

C 2.04871 4.49221 0.05111

N 0.32752 7.52932 0.13893

C 0.58756 6.20314 0.09995

H 1.09394 8.18606 0.16936

N 1.07590 3.58684 0.02070

C -0.15865 4.10295 0.03397

N -0.47507 5.38382 0.07369

H -0.63364 7.83857 0.15523

H 6.24953 3.95139 -0.09763

H 5.61473 -0.47871 -0.07777

1BH,3H

C 3.78647 3.12633 -0.12281

N 5.09576 3.34993 0.01611

C 5.81604 2.24860 0.24889

C 4.02669 0.92056 0.17020

N 3.19811 1.94426 -0.06187

H 7.55496 3.32064 0.35977

N 5.33329 0.99891 0.34219

N 3.03669 4.24962 -0.39272

N 7.14819 2.39722 0.40494

N 3.35577 -0.28267 0.20330

H 7.71317 1.58144 0.59420

H 2.35935 -0.18107 0.04167

H 3.57607 5.07366 -0.63203

N -0.47418 6.74119 -1.94664

H -1.46768 6.84977 -2.09558

N -0.94449 4.76121 -0.89207

C -0.02521 5.64023 -1.30747

H 0.18879 7.40831 -2.31464

N -1.28564 2.77238 0.28382

C -0.44509 3.73180 -0.22691

N 1.29820 5.51354 -1.15222

C 1.66784 4.39711 -0.52369

N 0.85279 3.48945 -0.01111

H -0.79249 2.00604 0.73020

N -4.88201 5.42661 1.43910

H -5.84415 5.61425 1.68097

B -5.44724 2.99397 0.97225

C -4.49080 4.17372 1.09470

H -4.18296 6.15025 1.55825

C -4.73742 1.75692 0.58317

N -3.16076 4.05232 0.89036

C -2.66085 2.91059 0.51550

N -3.38189 1.78293 0.35081

N 2.73081 -2.44726 0.14393

C 3.75608 -1.59446 0.29130

N 2.12812 -4.71159 0.03202

C 3.07262 -3.72117 0.19943

N 5.01904 -1.92035 0.49369

C 5.23852 -3.24337 0.53676

N 4.30887 -4.19117 0.39208

H 2.51906 -5.64715 0.01760

N 6.51079 -3.64211 0.74291

H 7.23043 -2.94240 0.85786

H 6.72339 -4.62850 0.78790

N -1.94598 -2.51819 -0.19616

N -1.86531 -4.83699 -0.57631

C -1.24381 -3.70696 -0.29986

H -1.35044 -1.71598 -0.02564

N -1.64512 -7.09658 -0.88622

C -1.06250 -5.90967 -0.61730

H -2.64085 -7.12607 -1.05382

N 0.07329 -3.55836 -0.09608

C 0.76431 -4.68625 -0.16990

N 0.25563 -5.89760 -0.41642

H -1.07668 -7.92947 -0.94494

N -5.38179 0.52856 0.39101

H -6.39008 0.57207 0.48262

N -5.89160 -1.65304 0.05365

C -4.92952 -0.72688 0.15322

N -6.36668 -3.86953 -0.29570

C -5.45123 -2.88761 -0.16497

H -7.34941 -3.64899 -0.21784

N -3.61220 -0.96684 0.04109

C -3.30129 -2.26330 -0.14730

N -4.16291 -3.25251 -0.26299

H -6.04971 -4.81633 -0.44882

H -6.64725 3.04528 1.17532

H -2.93598 0.90382 0.06633

3BH,2H

C -5.01739 0.08342 -0.01523

N -6.14619 -0.62802 -0.00517

C -5.95561 -1.94955 0.05187

C -3.73027 -1.74817 0.06185

N -3.79083 -0.40933 0.01017

H -7.96902 -2.31549 0.05598

N -4.76671 -2.56671 0.09263

N -5.22323 1.44944 -0.10528

N -7.05128 -2.73702 0.07004

N -2.43674 -2.22629 0.06981

H -6.93253 -3.73881 0.12361

H -1.73144 -1.49759 0.03092

H -6.17418 1.71292 -0.33710

N -4.31463 5.84797 -0.99160

H -3.65425 6.60746 -1.07471

N -2.63118 4.55301 -0.10579

C -3.90354 4.64723 -0.53039

H -5.24397 5.92823 -1.38056

N -1.05344 3.11373 0.84629

C -2.30606 3.34819 0.37929

N -4.79047 3.65165 -0.53797

C -4.32580 2.49172 -0.05652

N -3.12494 2.29452 0.45111

H -0.92895 2.15957 1.16768

N 0.95761 7.12737 -0.47455

H 1.83731 7.62018 -0.59442

N 2.37578 5.34404 0.05078

C 1.16386 5.82025 -0.04870

H 0.28272 7.66416 0.06378

C 2.57876 4.05822 0.42098

N 0.05454 5.08274 0.18242

C 0.14099 3.80825 0.66698

B 1.46035 3.16672 0.91478

N -0.57649 -3.53159 0.00138

C -1.91738 -3.50515 0.06537

N 1.29838 -4.96711 -0.08994

C -0.06045 -4.75077 -0.01397

N -2.71704 -4.55379 0.11955

C -2.07772 -5.73152 0.09902

N -0.75672 -5.89312 0.03097

H 1.53642 -5.95252 -0.12105

N -2.84405 -6.84165 0.15140

H -3.84740 -6.74109 0.21024

H -2.40510 -7.75124 0.14648

N 3.34561 -0.82169 -0.03638

N 4.67431 -2.74492 -0.22500

C 3.48254 -2.18591 -0.11702

H 2.38191 -0.51474 0.04931

N 5.83803 -4.71125 -0.39411

C 4.64819 -4.08313 -0.28343

H 6.68201 -4.15689 -0.42422

N 2.30473 -2.83416 -0.07008

C 2.40965 -4.14901 -0.13550

N 3.55032 -4.84010 -0.24140

H 5.86764 -5.71969 -0.43929

N 3.92138 3.70514 0.30333

H 4.57162 4.46679 0.13710

N 5.79125 2.39644 0.06997

C 4.45775 2.45374 0.16689

N 7.61408 1.02337 -0.17719

C 6.27434 1.15970 -0.06588

H 8.20245 1.84299 -0.13416

N 3.64543 1.40671 0.11302

C 4.25014 0.22412 -0.01013

N 5.55274 0.02615 -0.10128

H 8.00582 0.09639 -0.26262

H 1.52401 2.06707 1.40990

H -0.88058 5.43017 -0.06365

2BH,3H

C -3.47607 3.51005 0.17371

N -4.76246 3.86415 0.08844

C -5.59823 2.84439 -0.12245

C -3.95097 1.34324 -0.12282

N -3.01428 2.27380 0.08154

H -7.22362 4.08673 -0.15629

N -5.24884 1.55295 -0.24253

N -2.61023 4.54895 0.41695

N -6.91426 3.12773 -0.22487

N -3.40773 0.07669 -0.18762

H -7.56550 2.37468 -0.39545

H -2.40128 0.07528 -0.05902

H -3.05639 5.42357 0.66801

N 1.16327 6.74022 1.78809

H 2.17024 6.78777 1.85421

N 1.41917 4.72615 0.72534

C 0.59662 5.67585 1.17833

H 0.57597 7.48054 2.14377

N 1.54358 2.70995 -0.43800

C 0.80949 3.72217 0.10668

N -0.73913 5.65944 1.08805

C -1.22612 4.56973 0.49095

N -0.51968 3.58734 -0.03643

H 0.96610 2.02457 -0.91695

N 6.24930 4.28307 -1.51796

H 7.20833 3.96319 -1.58463

N 5.69968 2.17922 -0.86292

C 5.27057 3.42172 -1.15246

H 6.01926 5.23243 -1.77441

C 4.82438 1.26751 -0.50709

B 3.79129 3.83261 -1.08914

C 2.93697 2.71613 -0.65125

N 3.51242 1.47741 -0.37904

N -3.00946 -2.14227 -0.17586

C -3.94358 -1.18352 -0.28220

N -2.64347 -4.45921 -0.12087

C -3.48412 -3.37122 -0.24316

N -5.23843 -1.37475 -0.45594

C -5.59531 -2.66617 -0.51626

N -4.76600 -3.70832 -0.41238

H -3.12865 -5.34958 -0.13569

N -6.90675 -2.92703 -0.69628

H -7.55213 -2.15422 -0.77990

H -7.22199 -3.88494 -0.75225

N 1.62494 -2.69151 0.20882

N 1.32002 -5.01278 0.43877

C 0.80924 -3.81279 0.24507

H 1.11433 -1.83139 0.04221

N 0.87965 -7.25539 0.60881

C 0.41509 -6.00258 0.42228

H 1.86941 -7.39436 0.75492

N -0.48502 -3.52311 0.05831

C -1.28473 -4.58042 0.06726

N -0.89582 -5.84835 0.23795

H 0.23339 -8.03176 0.61124

N 5.35572 0.00872 -0.23489

H 6.36792 -0.01188 -0.28906

N 5.63967 -2.19670 0.19547

C 4.77254 -1.19959 0.01229

N 5.88576 -4.43239 0.64726

C 5.07352 -3.38076 0.42482

H 6.88743 -4.30068 0.63858

N 3.44050 -1.30657 0.04375

C 2.99477 -2.56451 0.24333

N 3.75486 -3.62081 0.44868

H 5.47572 -5.33977 0.81858

H 3.40401 4.94276 -1.38826

H 2.94769 0.67269 -0.09065

3BH,1H

C 4.59243 2.08628 -0.14948

N 5.28449 3.16393 0.23151

C 4.55468 4.28074 0.30590

C 2.68282 3.24173 -0.33148

N 3.31286 2.06440 -0.47018

H 6.18570 5.39593 0.83982

N 3.24208 4.37769 0.05265

N 5.34562 0.93073 -0.21339

N 5.18954 5.41508 0.67460

N 1.35157 3.16882 -0.67067

H 4.66884 6.28027 0.69968

H 1.12612 2.26802 -1.07915

H 6.34298 1.07867 -0.10855

N 6.69633 -3.42022 -0.50260

H 6.46818 -4.40354 -0.53878

N 4.45047 -3.01083 -0.30692

C 5.69526 -2.52116 -0.38946

H 7.64899 -3.09676 -0.59099

N 2.18187 -2.43351 -0.07803

C 3.51342 -2.08689 -0.19321

N 6.02542 -1.22851 -0.36799

C 4.98260 -0.39874 -0.26446

N 3.71274 -0.76160 -0.17476

H 1.55330 -1.63768 -0.03886

N 2.17265 -7.05629 0.24176

H 1.65548 -7.91764 0.34456

N 0.18158 -5.91497 0.31400

C 1.50997 -5.88042 0.20835

H 3.17967 -7.05210 0.16313

C -0.40587 -4.71328 0.27488

N 2.25048 -4.77272 0.07126

C 1.55109 -3.65291 0.04786

N 0.21520 -3.55215 0.14685

B -1.11449 3.46430 -0.87265

C 0.24925 3.94834 -0.37460

N -3.59792 4.07096 -0.39368

C -2.20751 4.27219 -0.30528

N 0.42978 5.03447 0.38131

C -0.61748 5.72203 0.77358

N -1.88476 5.36632 0.49161

H -4.19771 4.81516 -0.73812

N -0.41530 6.89276 1.48168

H 0.55312 6.99447 1.76836

H -1.08354 7.12629 2.20898

N -3.40365 -0.47103 0.17882

N -5.48099 0.49869 -0.36851

C -4.19783 0.62923 -0.07803

H -2.41597 -0.24870 0.25040

N -7.41933 1.59068 -0.91548

C -6.09694 1.65819 -0.64370

H -7.85925 0.68253 -0.96366

N -3.51764 1.77740 -0.03027

C -4.22035 2.85223 -0.36636

N -5.52117 2.86207 -0.67849

H -7.91000 2.42609 -1.20066

N -1.77847 -4.79653 0.37970

H -2.10926 -5.75288 0.44686

N -4.01258 -4.44978 0.39282

C -2.80479 -3.87588 0.34936

N -6.28138 -4.09519 0.40994

C -5.03074 -3.58863 0.36134

H -6.41258 -5.09452 0.47348

N -2.56750 -2.57732 0.28344

C -3.67510 -1.81833 0.24576

N -4.92077 -2.25553 0.28644

H -7.06690 -3.46040 0.38997

H -1.24747 2.50564 -1.60125

H -2.64528 5.91663 0.88013

2BH,1H

C -4.15301 -2.51175 -0.55538

N -5.47687 -2.47451 -0.74677

C -6.00557 -1.25152 -0.68659

C -4.03266 -0.29513 -0.27420

N -3.37985 -1.46313 -0.32307

H -7.88511 -1.95989 -1.07961

N -5.32942 -0.11759 -0.44975

N -3.62972 -3.78379 -0.58800

N -7.33749 -1.13562 -0.87803

N -3.19402 0.77170 -0.02397

H -7.75695 -0.21712 -0.85292

H -2.21228 0.51618 0.00671

H -4.33380 -4.51122 -0.64150

N -0.98039 -7.39905 0.28791

H -0.06505 -7.79052 0.45933

N 0.03495 -5.35482 0.08048

C -1.09370 -6.07144 0.06208

H -1.80851 -7.97674 0.28365

N 0.94548 -3.22821 -0.21378

C -0.13068 -4.06276 -0.17047

N -2.31882 -5.58619 -0.15784

C -2.34945 -4.26774 -0.38038

N -1.30737 -3.46095 -0.42449

H 0.73108 -2.29978 -0.56523

N 5.05516 -5.05607 2.20157

H 5.75397 -4.61190 2.79125

N 4.90131 -3.17270 0.86272

C 4.27742 -4.26871 1.37952

H 4.54365 -5.79296 2.67369

C 4.27929 -2.29852 0.01730

B 2.83370 -4.52424 1.02969

C 2.27407 -3.42649 0.16276

N 3.04231 -2.41513 -0.32538

N -2.29449 2.81677 0.33950

C -3.42638 2.10707 0.21334

N -1.41263 4.96887 0.68687

C -2.48231 4.10405 0.56873

N -4.65523 2.58176 0.31122

C -4.71525 3.89823 0.55532

N -3.66622 4.71275 0.69076

H -1.68830 5.94010 0.78098

N -5.94629 4.43995 0.67188

H -6.75446 3.84060 0.58169

H -6.04074 5.42627 0.86738

N 2.33154 2.29508 0.24498

N 2.59075 4.62615 0.23720

C 1.80256 3.56856 0.30719

H 1.67996 1.56297 0.50775

N 2.69779 6.91522 0.29205

C 1.94466 5.79587 0.34231

H 3.69855 6.82754 0.18588

N 0.47489 3.58141 0.47913

C -0.05257 4.79313 0.54631

N 0.62660 5.94317 0.49208

H 2.25463 7.81847 0.38037

N 5.12535 -1.28956 -0.47151

H 5.94803 -1.56626 -0.99802

N 5.71981 0.74553 -1.30542

C 4.82068 0.04297 -0.61217

N 6.31210 2.83219 -2.06319

C 5.43346 2.04674 -1.40351

H 7.10867 2.41086 -2.51952

N 3.72163 0.52263 -0.04831

C 3.52847 1.83168 -0.25449

N 4.34219 2.64384 -0.90799

H 6.07884 3.80424 -2.21073

H 2.25211 -5.46436 1.51941

H 5.86179 -2.95864 1.11884

Group 1:
1BH,5H

C -1.88333 4.44067 -0.31019

N -1.70685 5.74707 -0.49350

C -0.41995 6.12198 -0.45756

C 0.29831 4.03027 -0.09725

N -0.95751 3.53496 -0.10686

H -0.89640 8.08281 -0.80041

N 0.61883 5.30154 -0.26429

N -3.23363 4.07913 -0.33398

N -0.14537 7.42890 -0.63091

N 1.24955 3.06850 0.09126

H 0.81981 7.72934 -0.62412

H 0.88404 2.12179 0.12872

H -3.82659 4.88733 -0.48609

N -7.26436 1.81537 0.01887

H -7.75297 0.92568 -0.00535

N -5.32556 0.58860 -0.07840

C -5.91681 1.73333 -0.05964

H -7.76490 2.64151 -0.28262

N -3.28440 -0.53387 -0.01648

C -3.96722 0.58486 -0.10243

N -5.21469 2.92452 -0.09258

C -3.82967 2.85310 -0.22748

N -3.21431 1.72917 -0.25743

H -2.27917 -0.44486 -0.15078

N -6.55167 -3.58960 1.26153

H -6.74108 -4.47957 1.70615

B -4.28662 -4.53643 0.50813

C -5.25310 -3.40073 0.80816

H -6.97074 -2.78464 1.71730

C -2.94748 -4.02386 0.05566

N -4.89702 -2.12320 0.64326

C -3.67640 -1.89116 0.18912

N -2.69197 -2.72022 -0.10000

N 3.18377 1.90974 0.23935

C 2.63196 3.12358 0.19746

N 5.21719 0.74404 0.38179

C 4.50576 1.91473 0.34424

N 3.26158 4.28025 0.26073

C 4.59539 4.16040 0.37423

N 5.26935 3.01201 0.41838

H 6.21988 0.87591 0.45505

N 5.30378 5.30678 0.44878

H 4.81345 6.18949 0.42753

H 6.30836 5.26521 0.54644

N 2.05707 -2.60192 -0.05765

N 4.32783 -3.18613 0.04732

C 3.37845 -2.26447 0.04747

H 1.41936 -1.82008 0.04252

N 6.57420 -3.60719 0.17974

C 5.56903 -2.70527 0.17018

H 6.34658 -4.58938 0.11397

N 3.58114 -0.93629 0.16300

C 4.84753 -0.58878 0.27004

N 5.89730 -1.41417 0.28290

H 7.52710 -3.29255 0.29171

N -1.91818 -4.94730 -0.25071

H -2.24084 -5.90484 -0.32487

N 0.02806 -6.04813 -0.61130

C -0.57260 -4.86879 -0.33979

N 2.01048 -7.15449 -0.95136

C 1.34958 -6.00010 -0.68718

H 1.47952 -7.98575 -1.16760

N 0.08526 -3.71773 -0.15294

C 1.40880 -3.81874 -0.25187

N 2.11048 -4.90460 -0.52002

H 3.00950 -7.11882 -1.09306

H -4.55419 -5.72114 0.65309

H -5.66950 3.79831 0.14325

1BH,16H

C -0.01687 -4.83659 -0.05331

N -0.70888 -5.98590 -0.03844

C -2.03026 -5.83929 0.01263

C -1.89040 -3.61713 0.02313

N -0.55583 -3.62355 -0.02404

H -2.33652 -7.86264 0.01378

N -2.68724 -4.66781 0.04603

N 1.33657 -5.01797 -0.11264

N -2.78647 -6.95862 0.03236

N -2.41064 -2.33230 0.04359

H -3.79155 -6.87074 0.07527

H -1.69372 -1.61450 0.01700

H 1.61064 -5.99273 -0.16548

N 5.86695 -4.62315 -0.40565

H 6.68732 -4.03801 -0.47861

N 4.65092 -2.69915 -0.17136

C 4.65861 -4.02686 -0.27351

H 5.91612 -5.62093 -0.55050

N 3.28039 -0.81764 0.08900

C 3.44578 -2.15534 -0.03406

N 3.58281 -4.82224 -0.25459

C 2.43058 -4.15604 -0.12640

N 2.28134 -2.85569 -0.01293

H 2.31081 -0.52616 0.11089

N 7.65432 0.69083 0.45638

H 8.31935 1.39900 0.73473

B 5.84900 2.49340 0.23528

C 6.33251 1.03887 0.33163

H 7.85594 -0.27315 0.69956

C 4.37083 2.52556 0.10333

N 5.48300 0.01739 0.28980

C 4.18317 0.27423 0.13675

N 3.57667 1.43621 0.04782

N -3.72732 -0.49487 0.04797

C -3.68813 -1.84108 0.06859

N -5.12692 1.39332 0.04533

C -4.94043 0.01265 0.06788

N -4.74313 -2.63623 0.10832

C -5.91953 -1.99697 0.12585

N -6.08484 -0.66778 0.10640

H -6.10167 1.67381 0.05389

N -7.02918 -2.76120 0.16633

H -6.92904 -3.76667 0.18330

H -7.93901 -2.32283 0.18308

N -0.81931 3.01185 -0.03703

N -2.59833 4.53975 -0.06944

C -2.17228 3.27595 -0.03572

H -0.57221 2.02638 0.00716

N -4.45521 5.90559 -0.08136

C -3.94287 4.65948 -0.05412

H -3.84547 6.70999 -0.07386

N -2.92900 2.19216 0.00378

C -4.23727 2.42633 0.00897

N -4.79800 3.64289 -0.01675

H -5.45926 6.02118 -0.04807

N 3.73304 3.79915 0.02175

H 4.40218 4.56446 0.01477

N 2.32212 5.58364 -0.11966

C 2.48933 4.21634 -0.03689

N 0.86885 7.36896 -0.21609

C 1.11369 6.03804 -0.16615

H 1.67473 7.97354 -0.32200

N 1.43808 3.34784 -0.01527

C 0.25943 3.85874 -0.06441

N 0.01553 5.20626 -0.14446

H -0.04030 7.72721 -0.47418

H 6.57016 3.48205 0.27861

H -0.97069 5.50000 -0.13687

1BH,17H

C 2.40087 -4.24780 -0.00191

N 2.35164 -5.58796 -0.00276

C 1.12128 -6.09586 -0.00250

C 0.17008 -4.08050 -0.00085

N 1.34553 -3.44457 -0.00093

H 1.82756 -8.01712 -0.00415

N -0.01837 -5.38679 -0.00156

N 3.67656 -3.75160 -0.00217

N 0.99773 -7.44160 -0.00328

N -0.90455 -3.20807 0.00017

H 0.07343 -7.84830 -0.00323

H -0.62850 -2.23145 0.00026

H 4.39072 -4.47112 -0.00287

N 7.44038 -1.18219 -0.00148

H 7.87478 -0.27050 -0.00051

N 5.42209 -0.10348 -0.00015

C 6.08862 -1.25756 -0.00106

H 7.98769 -2.03022 -0.00163

N 3.28075 0.85716 0.00107

C 4.09863 -0.22421 0.00017

N 5.54517 -2.47924 -0.00162

C 4.20759 -2.46694 -0.00136

N 3.43216 -1.40594 -0.00055

H 2.29552 0.62131 0.00031

N 6.32425 4.34918 0.00966

H 6.60120 5.31891 0.01054

B 3.85335 5.00888 0.00449

C 5.00443 3.99636 0.00652

H 7.02599 3.61921 0.01068

C 2.55887 4.28914 0.00161

N 4.77231 2.68609 0.00540

C 3.51320 2.25173 0.00239

N 2.39832 2.95392 0.00052

N -2.96062 -2.25670 0.00092

C -2.26171 -3.40656 0.00083

N -5.12318 -1.32949 0.00164

C -4.26872 -2.41994 0.00135

N -2.77891 -4.62268 0.00115

C -4.11719 -4.65424 0.00164

N -4.91969 -3.58566 0.00177

H -6.10367 -1.58855 0.00195

N -4.69863 -5.87194 0.00211

H -4.11047 -6.69342 0.00195

H -5.70590 -5.94503 0.00246

N -2.33895 2.35190 -0.00034

N -4.67374 2.68591 -0.00014

C -3.64242 1.86455 -0.00006

H -1.63455 1.61851 0.00064

N -6.96275 2.84182 0.00027

C -5.86276 2.06037 0.00032

H -6.85403 3.84569 0.00071

N -3.68439 0.53141 0.00057

C -4.91105 0.02516 0.00113

N -6.04169 0.74068 0.00099

H -7.87747 2.41284 0.00148

N 1.36921 5.09694 -0.00047

H 1.63742 6.07630 -0.00086

N -0.75145 5.99748 -0.00362

C 0.07699 4.85745 -0.00146

N -2.87229 6.94937 -0.00689

C -2.10496 5.84331 -0.00475

H -2.49820 7.88802 -0.00870

N -0.50165 3.66228 -0.00046

C -1.82441 3.61321 -0.00146

N -2.66935 4.66844 -0.00378

H -3.87529 6.80872 -0.00806

H 3.96953 6.23089 0.00517

H -0.33781 6.92238 -0.00412

1BH,6H

C -4.76676 1.08083 -0.47692

N -5.66803 2.04087 -0.59765

C -5.16513 3.27729 -0.45915

C -3.07594 2.52485 -0.11854

N -3.46837 1.23135 -0.24121

H -6.99349 4.13691 -0.76155

N -3.88115 3.56400 -0.22042

N -5.28646 -0.19752 -0.61705

N -6.01580 4.30955 -0.57116

N -1.74167 2.68926 0.10493

H -5.65875 5.25105 -0.47726

H -1.17447 1.85055 0.04302

H -6.28575 -0.23379 -0.79282

N -5.56027 -4.76364 -0.64416

H -5.11879 -5.67030 -0.54675

N -3.52211 -3.83260 -0.24139

C -4.80922 -3.66625 -0.49454

H -6.54657 -4.67838 -0.84882

N -1.48409 -2.77897 0.16404

C -2.77409 -2.74883 -0.10272

N -5.45531 -2.47329 -0.62434

C -4.69452 -1.42231 -0.49832

N -3.36480 -1.49314 -0.25235

H -0.93885 -1.92779 0.06117

N -1.04633 -7.06856 1.91224

H -0.44762 -7.72279 2.40104

B 1.06543 -5.99721 0.91495

C -0.40781 -6.00080 1.29754

H -1.91358 -6.83939 2.38843

C 1.47071 -4.69809 0.28759

N -1.19366 -4.94332 1.05784

C -0.63772 -3.91755 0.44009

N 0.59658 -3.70528 0.05191

N 0.33073 3.59083 0.25180

C -0.97578 3.83746 0.31637

N 2.46103 4.57643 0.39064

C 1.09690 4.66003 0.44845

N -1.55533 4.99254 0.56652

C -0.68406 6.00136 0.74800

N 0.64204 5.89432 0.69534

H 2.93217 5.46925 0.48732

N -1.20744 7.21754 1.00398

H -2.20992 7.31622 1.07853

H -0.58944 7.99745 1.17947

N 3.34251 0.07815 -0.11759

N 5.12883 1.59866 -0.22465

C 3.83743 1.34888 -0.08170

H 2.34922 0.01889 0.07437

N 6.76384 3.20037 -0.27334

C 5.45645 2.89238 -0.14155

H 7.42785 2.45237 -0.41711

N 2.88104 2.27876 0.12521

C 3.33103 3.51414 0.17826

N 4.60179 3.90258 0.05956

H 7.06008 4.16355 -0.20846

N 2.81581 -4.49289 -0.11915

H 3.36424 -5.34270 -0.17707

N 4.85442 -3.62837 -0.63149

C 3.56806 -3.38870 -0.29430

N 6.90385 -2.72548 -1.13166

C 5.60451 -2.54782 -0.79021

H 7.25808 -3.66192 -1.26113

N 3.05880 -2.16122 -0.12106

C 3.92650 -1.17290 -0.30732

N 5.19840 -1.27224 -0.64491

H 7.49082 -1.91473 -1.26177

H 1.83169 -6.92659 1.13098

H -2.86957 -0.59652 -0.14577

1BH,18H

C 3.62893 3.30426 -0.14219

N 4.93371 3.59831 -0.10161

C 5.73346 2.53561 0.00859

C 4.02510 1.10817 0.01794

N 3.12138 2.08300 -0.09182

H 7.41097 3.70659 0.02516

N 5.33517 1.25308 0.07785

N 2.79658 4.38437 -0.27877

N 7.06433 2.75839 0.05518

N 3.42145 -0.13333 0.06178

H 7.68989 1.97156 0.15411

H 2.40980 -0.07139 0.01775

H 3.26598 5.26152 -0.47213

N -0.89728 6.83574 -1.39360

H -1.90187 6.91492 -1.46718

N -1.22368 4.73176 -0.55163

C -0.36873 5.69447 -0.89751

H -0.28504 7.58146 -1.69057

N -1.42863 2.61670 0.40841

C -0.65742 3.65108 -0.02672

N 0.96612 5.62393 -0.80824

C 1.40944 4.45969 -0.33137

N 0.66949 3.45484 0.09614

H -0.90789 1.78737 0.66303

N -5.13405 5.03147 1.66225

H -6.09277 5.12996 1.96400

B -5.61560 2.65889 0.84622

C -4.69205 3.82638 1.19489

H -4.44546 5.69821 1.99248

C -4.81436 1.49781 0.40826

N -3.36801 3.72962 1.05940

C -2.83249 2.61352 0.58787

N -3.45404 1.48303 0.28348

N 2.87905 -2.31609 0.11224

C 3.88223 -1.42105 0.11353

N 2.34505 -4.59639 0.16792

C 3.27443 -3.57119 0.15660

N 5.17183 -1.70202 0.15725

C 5.44407 -3.01426 0.20257

N 4.53787 -3.99819 0.20419

H 2.75794 -5.51888 0.25290

N 6.74520 -3.36498 0.25010

H 7.44777 -2.63872 0.24869

H 6.99848 -4.34220 0.28348

N -1.77978 -2.49992 -0.19654

N -1.68570 -4.83128 0.08613

C -1.05171 -3.68702 -0.05266

H -1.20056 -1.66908 -0.12889

N -1.45809 -7.09709 0.35588

C -0.87180 -5.89078 0.22467

H -2.46661 -7.15412 0.37572

N 0.26666 -3.49457 -0.03733

C 0.97340 -4.61087 0.12323

N 0.45875 -5.83851 0.24555

H -0.88435 -7.91631 0.50014

N -5.46810 0.28395 0.03190

H -6.48109 0.33483 0.00040

N -5.79999 -1.90647 -0.58069

C -4.97691 -0.90315 -0.26193

N -5.98745 -4.10138 -1.20114

C -5.21611 -3.07019 -0.82012

H -6.98287 -3.96484 -1.31470

N -3.61324 -1.07951 -0.20763

C -3.12435 -2.34003 -0.38256

N -3.89268 -3.33965 -0.71103

H -5.55536 -4.99623 -1.38628

H -6.83475 2.66515 0.92937

H -3.10837 -0.15055 -0.00645

3BH,16H

C 0.22215 -4.78800 -0.06775

N -0.39741 -5.96764 -0.08787

C -1.73238 -5.87668 -0.07069

C -1.69654 -3.63834 -0.01350

N -0.34743 -3.60135 -0.03082

H -1.95023 -7.91102 -0.12266

N -2.43114 -4.73692 -0.03370

N 1.61009 -4.92291 -0.07124

N -2.43634 -7.02617 -0.09207

N -2.27550 -2.39715 0.02968

H -3.44579 -6.98034 -0.08071

H -1.61513 -1.62632 0.03424

H 1.91292 -5.89009 -0.02378

N 6.10677 -4.19980 0.14735

H 6.90494 -3.58246 0.17233

N 4.73252 -2.36085 -0.06600

C 4.86013 -3.70126 0.03298

H 6.22636 -5.19959 0.24042

N 3.20277 -0.60745 -0.30737

C 3.46876 -1.95426 -0.19108

N 3.84986 -4.56416 0.02787

C 2.63049 -4.01957 -0.08666

N 2.38846 -2.71865 -0.21387

H 2.21583 -0.36594 -0.36823

N 7.50119 1.22276 0.06908

H 8.08502 2.04988 0.03648

N 5.66799 2.61534 0.00288

C 6.16466 1.41823 0.00732

H 7.91703 0.32438 -0.13378

C 4.29893 2.71805 -0.01620

N 5.37422 0.28659 -0.02228

C 4.02585 0.48010 -0.18221

N 3.48948 1.64511 -0.21621

N -3.72410 -0.65254 0.05450

C -3.60136 -1.98200 0.05080

N -5.27353 1.12045 0.03683

C -4.97960 -0.21697 0.06084

N -4.58282 -2.86360 0.06137

C -5.80560 -2.30842 0.06571

N -6.06542 -1.00307 0.06264

H -6.26037 1.31962 -0.08196

N -6.85666 -3.15733 0.07198

H -6.68328 -4.15199 0.08937

H -7.79546 -2.78544 0.09288

N -1.21759 3.21700 0.61900

N -3.07682 4.52520 0.00879

C -2.53191 3.35334 0.31283

H -1.00043 2.25396 0.84769

N -4.99176 5.64710 -0.55096

C -4.38511 4.47490 -0.25456

H -4.44224 6.49470 -0.53406

N -3.20386 2.17652 0.36101

C -4.47552 2.26058 0.05070

N -5.14781 3.37361 -0.25742

H -5.98375 5.66284 -0.73690

N 3.75074 3.89809 0.15992

H 4.38487 4.69531 0.17923

N 2.17545 5.52740 -0.09500

C 2.36463 4.23180 0.25055

N 0.75010 7.33306 -0.37931

C 0.93785 5.97731 -0.11486

H 1.51297 7.76657 -0.88653

B 1.26993 3.31808 0.67208

C -0.07115 4.01955 0.46553

N -0.19513 5.27774 0.10012

H -0.17645 7.54859 -0.72962

H 5.70150 -0.68628 0.02954

H 1.34214 2.17838 1.09496

2BH,4H

C -2.58727 4.17017 0.09958

N -3.76143 4.79933 0.13271

C -4.81588 3.97511 0.11031

C -3.53093 2.14619 0.03339

N -2.39657 2.86950 0.05145

H -6.13605 5.53671 0.18083

N -4.75668 2.63760 0.05989

N -1.50295 5.04093 0.12486

N -6.04299 4.53165 0.14069

N -3.29468 0.79643 -0.01249

H -6.85374 3.92860 0.12662

H -2.30919 0.55276 -0.01219

H -1.76891 6.01807 0.18159

N 2.64899 6.93884 0.15203

H 3.65804 6.86866 0.13326

N 2.45424 4.63923 -0.00494

C 1.85579 5.85635 0.09693

H 2.20746 7.84597 0.23288

N 2.26511 2.34375 -0.15114

C 1.67146 3.51108 -0.05890

N 0.55456 5.98380 0.13834

C -0.15673 4.82404 0.07942

N 0.33934 3.61020 -0.01309

H 1.65240 1.52914 -0.14567

N 7.38560 2.67392 -0.28711

H 8.17753 2.05801 -0.43692

N 6.13984 0.76982 -0.12445

C 6.14286 2.09148 -0.23841

H 7.46534 3.62258 -0.62599

C 4.95427 0.13810 -0.04658

B 4.84200 2.90053 -0.29101

C 3.66005 2.00892 -0.17213

N 3.76040 0.66007 -0.05927

N -3.44911 -1.45721 -0.06068

C -4.12887 -0.30535 -0.05031

N -3.66133 -3.79678 -0.10690

C -4.21209 -2.53862 -0.09692

N -5.44039 -0.16655 -0.07514

C -6.10654 -1.33187 -0.11336

N -5.55024 -2.54318 -0.12537

H -4.34949 -4.54115 -0.13018

N -7.45404 -1.26052 -0.14147

H -7.89716 -0.35287 -0.13593

H -7.99724 -2.11148 -0.17390

N 0.91825 -3.15107 0.02144

N 0.07309 -5.34106 -0.00397

C -0.13117 -4.03488 -0.00651

H 0.63950 -2.17680 -0.01631

N -0.89143 -7.41773 -0.03878

C -1.04428 -6.07606 -0.03844

H 0.04273 -7.80167 -0.01677

N -1.33357 -3.42998 -0.04194

C -2.35783 -4.26158 -0.07179

N -2.29005 -5.59624 -0.07247

H -1.70626 -8.01343 -0.06839

N 5.13356 -1.25320 0.06315

H 6.11257 -1.51207 0.09565

N 4.93573 -3.50016 0.20021

C 4.29438 -2.32028 0.10182

N 4.72469 -5.78137 0.33731

C 4.14244 -4.56367 0.23674

H 5.73075 -5.84787 0.39010

N 2.96968 -2.18057 0.04151

C 2.29295 -3.33130 0.08468

N 2.80084 -4.54553 0.18283

H 4.13786 -6.60207 0.37398

H 4.90070 4.12183 -0.43775

H 3.47839 4.51002 -0.04748

2BH,6H

C 3.95032 2.93068 -0.40429

N 5.26759 2.99566 -0.51612

C 5.88152 1.80955 -0.39927

C 3.95170 0.70298 -0.08973

N 3.22049 1.84090 -0.19500

H 7.72379 2.64812 -0.67724

N 5.26539 0.64229 -0.18650

N 3.32161 4.15881 -0.52369

N 7.21998 1.78865 -0.50614

N 3.21381 -0.42639 0.11064

H 7.70390 0.90425 -0.42781

H 2.20744 -0.31650 0.04616

H 3.94939 4.94157 -0.67811

N 0.10309 7.40050 -0.52226

H -0.86619 7.67905 -0.42883

N -0.56693 5.25388 -0.15955

C 0.41400 6.10417 -0.39000

H 0.83058 8.07916 -0.70011

N -1.16464 3.04229 0.20593

C -0.26515 3.96508 -0.04535

N 1.73577 5.79068 -0.50963

C 2.00870 4.51978 -0.40860

N 1.06907 3.56838 -0.19698

H -0.89811 2.06118 0.13497

N -5.71863 5.03238 1.40278

H -6.65438 4.70665 1.62082

N -5.31114 2.90740 0.67432

C -4.80099 4.08509 1.01146

H -5.37986 5.84010 1.90764

C -4.46900 1.94226 0.26200

B -3.29164 4.38298 0.94533

C -2.58074 3.18786 0.42926

N -3.17629 2.00588 0.11566

N 2.56990 -2.59532 0.21541

C 3.59852 -1.75402 0.30014

N 1.95041 -4.86052 0.31440

C 2.89457 -3.87213 0.39124

N 4.85424 -2.05993 0.55169

C 5.06506 -3.37872 0.70896

N 4.13012 -4.32431 0.63448

H 2.32657 -5.79804 0.40316

N 6.33168 -3.76558 0.96355

H 7.05177 -3.06307 1.05420

H 6.53040 -4.74360 1.12144

N -2.03831 -2.59891 -0.15905

N -2.05158 -4.94627 -0.26295

C -1.39711 -3.80490 -0.12750

H -1.42912 -1.80600 0.00693

N -1.90217 -7.23038 -0.31406

C -1.28248 -6.03820 -0.18755

H -2.90348 -7.24681 -0.44862

N -0.06795 -3.68483 0.06355

C 0.57746 -4.83110 0.11333

N 0.04217 -6.04808 0.00093

H -1.36379 -8.08274 -0.25588

N -5.18301 0.76116 -0.05545

H -6.18488 0.91262 -0.06060

N -5.86236 -1.35776 -0.48605

C -4.82920 -0.52973 -0.22718

N -6.52642 -3.51257 -0.91161

C -5.53309 -2.63251 -0.64180

H -7.47560 -3.17842 -0.99361

N -3.55640 -0.92600 -0.12540

C -3.37388 -2.23394 -0.29105

N -4.29164 -3.14397 -0.55580

H -6.29594 -4.48727 -1.03734

H -2.82066 5.44604 1.30486

H 1.40040 2.59945 -0.11281

2BH,16H

C 2.13638 4.37913 -0.00530

N 3.25810 5.11650 -0.01285

C 4.38727 4.41399 -0.02056

C 3.30237 2.47065 -0.01203

N 2.10076 3.05112 -0.00475

H 5.53657 6.10714 -0.02955

N 4.47545 3.07295 -0.02055

N 0.99438 5.12639 0.00375

N 5.55293 5.09730 -0.02911

N 3.21726 1.08597 -0.00949

H 6.42249 4.58399 -0.03496

H 2.25978 0.74966 -0.00324

H 1.16683 6.12555 0.00863

N -3.25249 6.77298 0.05689

H -4.24969 6.61627 0.10210

N -2.99962 4.49880 0.02732

C -2.42764 5.69968 0.03974

H -2.86031 7.70149 0.10877

N -2.61976 2.20630 -0.00780

C -2.15509 3.46930 0.00626

N -1.11101 5.93822 0.03312

C -0.36767 4.82549 0.01171

N -0.79896 3.58576 -0.00234

H -1.89406 1.49728 -0.01818

N -7.74683 1.83338 0.03035

H -8.44683 1.14922 -0.23680

N -6.25439 0.09431 -0.01439

C -6.42918 1.41622 -0.00378

H -7.92833 2.78901 -0.24789

C -4.99154 -0.33966 -0.00916

B -5.25648 2.40207 -0.00327

C -3.93668 1.67753 -0.00417

N -3.86872 0.32551 -0.00428

N 3.60892 -1.13951 -0.00075

C 4.15632 0.09194 -0.00851

N 4.05106 -3.44718 0.01080

C 4.48309 -2.12092 0.00155

N 5.45265 0.35082 -0.01417

C 6.23640 -0.73448 -0.01103

N 5.80941 -2.00478 -0.00299

H 4.80747 -4.12307 0.01780

N 7.56803 -0.52644 -0.01651

H 7.91356 0.42328 -0.02166

H 8.19856 -1.31553 -0.01356

N -0.53237 -3.02450 -0.00737

N 0.40282 -5.17636 0.02180

C 0.57280 -3.85336 0.00574

H -0.32472 -2.02864 -0.01176

N 1.47840 -7.21582 0.05292

C 1.56115 -5.87127 0.03538

H 0.57965 -7.67463 0.07477

N 1.72464 -3.20790 0.00140

C 2.80151 -3.98914 0.01449

N 2.77363 -5.32865 0.03160

H 2.33243 -7.75703 0.07511

N -4.97338 -1.76848 -0.01369

H -5.92407 -2.13121 -0.01379

N -4.47393 -3.98784 -0.02055

C -4.03308 -2.68582 -0.01520

N -3.93502 -6.22443 -0.00466

C -3.57852 -4.92254 -0.01544

H -4.92403 -6.43633 -0.05096

N -2.71443 -2.34956 -0.01441

C -1.86835 -3.32154 -0.01022

N -2.23096 -4.64276 -0.00944

H -3.26142 -6.97070 -0.09977

H -5.45142 3.59835 -0.01320

H -1.46302 -5.33028 0.00862

2BH,17H

C -2.67248 4.12687 0.08255

N -3.88163 4.70765 0.10878

C -4.90489 3.85719 0.09141

C -3.56708 2.07701 0.02957

N -2.45630 2.81822 0.04355

H -6.27386 5.37800 0.14606

N -4.81056 2.51841 0.05127

N -1.63827 5.02077 0.10162

N -6.15278 4.37586 0.11635

N -3.29548 0.71912 -0.00825

H -6.94412 3.74876 0.10317

H -2.30223 0.51064 -0.00800

H -1.93862 5.98829 0.13855

N 2.38995 7.15465 0.12315

H 3.39941 7.11406 0.13302

N 2.40041 4.86804 0.02340

C 1.69332 5.99441 0.08572

H 1.89463 8.03038 0.20285

N 2.27909 2.54636 -0.07896

C 1.67762 3.75078 -0.01440

N 0.35871 6.07960 0.11213

C -0.25274 4.88942 0.07271

N 0.32055 3.70965 0.01163

H 1.63458 1.76267 -0.06372

N 7.40557 2.78433 -0.30878

H 8.16939 2.17516 -0.58216

N 6.13447 0.88067 -0.15361

C 6.14816 2.21344 -0.24226

H 7.45200 3.73485 -0.65266

C 4.93332 0.30809 -0.05703

B 4.86425 3.04613 -0.24290

C 3.64396 2.17376 -0.11547

N 3.73965 0.82308 -0.02946

N -3.40685 -1.54277 -0.05279

C -4.10086 -0.38899 -0.04174

N -3.58826 -3.88815 -0.09591

C -4.15870 -2.62419 -0.08434

N -5.41824 -0.28353 -0.06219

C -6.06703 -1.45369 -0.09522

N -5.49265 -2.66084 -0.10722

H -4.27243 -4.63644 -0.11926

N -7.41512 -1.40365 -0.11795

H -7.86988 -0.50141 -0.11241

H -7.94709 -2.26162 -0.14688

N 0.96183 -3.11965 0.00606

N 0.17988 -5.34514 -0.03125

C -0.07500 -4.05241 -0.01944

H 0.63810 -2.15642 -0.04017

N -0.73926 -7.44744 -0.07613

C -0.92445 -6.11133 -0.06378

H 0.20114 -7.81522 -0.07668

N -1.27392 -3.47274 -0.04030

C -2.29098 -4.32655 -0.07241

N -2.17673 -5.65968 -0.08475

H -1.54187 -8.06000 -0.11533

N 5.10966 -1.11611 0.03643

H 6.11312 -1.28620 0.05202

N 4.92121 -3.40246 0.19283

C 4.29707 -2.14900 0.08149

N 4.77313 -5.71621 0.36593

C 4.15442 -4.52798 0.24902

H 5.77591 -5.81838 0.43947

N 2.97606 -2.10032 0.02635

C 2.31191 -3.25000 0.07558

N 2.85244 -4.48127 0.19167

H 4.18073 -6.53655 0.41501

H 4.90848 4.25190 -0.35624

H 5.93192 -3.45925 0.24082

3BH,5H

C 4.64514 1.84280 0.04288

N 5.32081 2.94181 0.57077

C 4.63090 4.14594 0.61984

C 2.78339 3.13316 -0.20354

N 3.44495 1.93210 -0.38217

H 6.31663 5.24137 1.09522

N 3.40746 4.26266 0.23469

N 5.39078 0.69160 0.01146

N 5.30563 5.20407 1.13261

N 1.50707 3.08915 -0.49974

H 4.80264 6.08535 1.10674

H 1.22159 2.16349 -0.81267

H 6.40043 0.77332 -0.04083

N 6.49395 -3.67792 -0.74824

H 6.20952 -4.64022 -0.86963

N 4.29020 -3.17818 -0.37677

C 5.55111 -2.74936 -0.49484

H 7.45334 -3.39396 -0.88741

N 2.07165 -2.52621 0.03112

C 3.40314 -2.23344 -0.11477

N 5.95007 -1.47441 -0.38434

C 4.95568 -0.62741 -0.13387

N 3.68325 -0.92207 0.02236

H 1.47062 -1.71462 0.13664

N 1.89312 -7.15921 0.11194

H 1.34311 -8.00494 0.16265

N -0.05718 -5.95123 0.18491

C 1.27231 -5.96060 0.11859

H 2.90164 -7.18928 0.06758

C -0.60791 -4.73036 0.19028

N 2.05561 -4.87269 0.05899

C 1.39001 -3.73421 0.06956

N 0.06223 -3.58610 0.13674

B -0.93322 3.35265 -0.66649

C 0.39774 3.96804 -0.39454

N -3.41475 4.16420 -0.37838

C -2.02496 4.37694 -0.35373

N 0.62925 5.23672 -0.02045

C -0.44012 5.99841 0.09373

N -1.72933 5.62880 -0.03040

H -3.97764 5.00637 -0.30808

N -0.23695 7.34987 0.36645

H 0.66347 7.55135 0.78524

H -1.02832 7.80505 0.80669

N -3.45448 -0.39436 0.00200

N -5.53253 0.73188 -0.10322

C -4.21244 0.76578 -0.09166

H -2.45641 -0.21331 -0.03395

N -7.44745 1.98827 -0.19517

C -6.09431 1.95092 -0.19929

H -7.95950 1.11847 -0.16844

N -3.44930 1.85147 -0.16777

C -4.10928 3.00245 -0.28361

N -5.44817 3.11033 -0.29599

H -7.91769 2.87399 -0.31346

N -1.97842 -4.76745 0.26339

H -2.34624 -5.71101 0.31857

N -4.19808 -4.34033 0.32919

C -2.97579 -3.80633 0.24166

N -6.45234 -3.90323 0.39150

C -5.18642 -3.44198 0.30335

H -6.61795 -4.89596 0.47334

N -2.69144 -2.52384 0.13745

C -3.77366 -1.71937 0.11632

N -5.03207 -2.11731 0.19700

H -7.21468 -3.24050 0.37277

H -1.04651 2.21452 -1.07195

H 6.13612 2.80019 1.15591

3BH,6H

C 4.89370 1.72504 0.07612

N 5.62602 2.80203 0.13083

C 4.92425 3.97240 0.05929

C 2.89287 2.98476 -0.10462

N 3.55187 1.74991 -0.05324

H 6.66486 5.03378 0.18123

N 3.61308 4.09994 -0.04532

N 5.52280 0.51393 0.15729

N 5.65882 5.09259 0.10435

N 1.58579 2.92007 -0.20746

H 5.18599 5.98614 0.04949

H 1.19363 1.98408 -0.25741

H 6.53195 0.57052 0.25107

N 6.32857 -3.96816 0.43810

H 5.98263 -4.91794 0.46466

N 4.15828 -3.28286 0.20530

C 5.45141 -2.96018 0.30438

H 7.31490 -3.76712 0.53063

N 1.97837 -2.48576 -0.03922

C 3.32407 -2.27038 0.07291

N 5.93941 -1.71155 0.28424

C 5.01172 -0.77603 0.16476

N 3.69933 -0.96593 0.05489

H 1.38637 -1.66255 -0.00402

N 1.68683 -7.07442 -0.65592

H 1.11614 -7.90216 -0.75641

N -0.23027 -5.85798 -0.32158

C 1.09383 -5.88182 -0.44232

H 2.68788 -7.10946 -0.78562

C -0.75798 -4.64250 -0.12739

N 1.89746 -4.80828 -0.37000

C 1.25825 -3.67885 -0.14820

N -0.06058 -3.51256 -0.02814

B -0.84719 3.26642 -0.28539

C 0.51021 3.87363 -0.21666

N -3.29123 4.24831 -0.15627

C -1.88763 4.37405 -0.19335

N 0.81277 5.17037 -0.13369

C -0.22896 5.98795 -0.12062

N -1.52678 5.65588 -0.12928

H -3.79572 5.12925 -0.14546

N 0.04308 7.35616 -0.11596

H 0.96300 7.58689 0.24131

H -0.71278 7.92211 0.25246

N -3.54782 -0.28723 0.10434

N -5.58711 0.92958 0.11969

C -4.26845 0.90187 0.07617

H -2.54799 -0.12532 0.03870

N -7.43558 2.28586 0.12355

C -6.08701 2.18135 0.07163

H -7.99046 1.44351 0.16638

N -3.45714 1.94746 -0.00404

C -4.05440 3.13552 -0.05908

N -5.38713 3.30964 -0.02047

H -7.86359 3.19841 0.06333

N -2.12360 -4.67306 -0.01864

H -2.50157 -5.61347 -0.06183

N -4.33629 -4.23292 0.16827

C -3.11263 -3.70507 0.08042

N -6.58136 -3.77838 0.34390

C -5.31446 -3.32421 0.24545

H -6.75664 -4.77281 0.35657

N -2.81349 -2.42400 0.06530

C -3.88659 -1.60728 0.13857

N -5.14677 -1.99678 0.23258

H -7.33601 -3.10880 0.40068

H -0.97646 2.06119 -0.38536

H 3.07556 0.83704 -0.07197

3BH,17H

C 4.71726 1.88602 0.02697

N 5.47249 2.93992 0.35232

C 4.80344 4.09998 0.33776

C 2.87613 3.13614 -0.26579

N 3.44951 1.91100 -0.30898

H 6.48558 5.15104 0.85220

N 3.50754 4.25957 0.05165

N 5.40807 0.68109 0.05147

N 5.49944 5.21809 0.64744

N 1.55823 3.09942 -0.59128

H 5.02259 6.10831 0.62324

H 1.27477 2.15220 -0.81466

H 6.40693 0.78407 0.18916

N 6.57135 -3.72900 -0.05266

H 6.30204 -4.70131 -0.09504

N 4.33756 -3.21578 -0.06919

C 5.60693 -2.78224 -0.04322

H 7.54192 -3.44994 -0.05210

N 2.08432 -2.53325 -0.05296

C 3.44206 -2.24584 -0.05132

N 5.99280 -1.50830 -0.00768

C 4.98663 -0.62347 -0.00377

N 3.69576 -0.93408 -0.02208

H 1.49568 -1.70674 -0.02644

N 1.87180 -7.15748 -0.11221

H 1.31790 -8.00191 -0.10455

N -0.07461 -5.94219 -0.01204

C 1.25908 -5.95588 -0.07006

H 2.88047 -7.18911 -0.16015

C -0.60596 -4.71701 0.02317

N 2.04462 -4.87392 -0.08994

C 1.39752 -3.72263 -0.04588

N 0.05795 -3.57633 0.00879

B -0.91039 3.35992 -0.65471

C 0.47308 3.97948 -0.45276

N -3.35671 4.16436 -0.21192

C -1.93646 4.36400 -0.28526

N 0.68272 5.23406 -0.11261

C -0.39917 6.01418 0.08086

N -1.66482 5.65069 0.04540

H -3.85173 5.05203 -0.13349

N -0.12025 7.35626 0.33363

H 0.81851 7.50941 0.68446

H -0.85097 7.84794 0.83483

N -3.44998 -0.38074 0.05380

N -5.52838 0.73557 -0.00006

C -4.17752 0.76783 0.00637

H -2.44559 -0.21908 0.01805

N -7.46060 1.96464 -0.00439

C -6.11670 1.90230 -0.02226

H -7.95842 1.08268 0.01815

N -3.42964 1.85541 -0.03715

C -4.02089 3.03614 -0.10318

N -5.42091 3.07888 -0.06117

H -7.97936 2.83055 -0.05233

N -1.99135 -4.75876 0.08592

H -2.34798 -5.70819 0.10844

N -4.21145 -4.33643 0.19038

C -2.98146 -3.81210 0.11800

N -6.46290 -3.90749 0.29676

C -5.19864 -3.44348 0.22043

H -6.62529 -4.90403 0.33367

N -2.70562 -2.51518 0.07441

C -3.78331 -1.72831 0.10631

N -5.04272 -2.10859 0.18079

H -7.22871 -3.24981 0.32500

H -1.05443 2.21209 -1.02476

H -5.90364 3.96941 -0.09330

3BH,18H

C 4.51430 2.28423 0.03894

N 5.17399 3.42247 0.27274

C 4.38893 4.50713 0.26823

C 2.53812 3.32811 -0.16149

N 3.22819 2.16712 -0.19878

H 5.98162 5.74401 0.62708

N 3.06570 4.52177 0.06877

N 5.32617 1.15890 0.05069

N 4.98222 5.70111 0.49067

N 1.21028 3.13864 -0.39497

H 4.41479 6.53675 0.47013

H 1.02143 2.15846 -0.56774

H 6.31386 1.36506 0.14780

N 6.91181 -3.12005 -0.05256

H 6.73794 -4.11426 -0.08784

N 4.63857 -2.82536 -0.05826

C 5.86135 -2.27127 -0.03839

H 7.85120 -2.74904 -0.05090

N 2.32978 -2.35317 -0.03900

C 3.65566 -1.94526 -0.03700

N 6.12278 -0.96525 -0.00548

C 5.03658 -0.18226 0.00487

N 3.78242 -0.61549 -0.00695

H 1.67308 -1.58022 -0.01527

N 2.49463 -6.97927 -0.08663

H 2.01041 -7.86566 -0.08100

N 0.45593 -5.92397 -0.01389

C 1.78752 -5.83138 -0.05539

H 3.50332 -6.93065 -0.11993

C -0.17226 -4.74616 0.01151

N 2.48354 -4.68891 -0.06839

C 1.74454 -3.59470 -0.03680

N 0.39671 -3.55546 0.00107

B -1.27422 3.23916 -0.52105

C 0.06241 3.93658 -0.31814

N -3.80645 3.90678 -0.25645

C -2.39710 4.18173 -0.28089

N 0.17633 5.22202 -0.05297

C -0.95922 5.93626 0.05092

N -2.20054 5.49199 -0.01908

H -4.39357 4.73633 -0.19907

N -0.78305 7.29747 0.24075

H 0.12930 7.55639 0.59607

H -1.57612 7.78728 0.63591

N -3.38063 -0.65280 -0.01153

N -5.54890 0.26959 0.06680

C -4.25744 0.38963 -0.01886

H -2.39011 -0.43202 -0.07404

N -7.58184 1.30471 0.13773

C -6.25024 1.43601 0.04499

H -7.98144 0.37940 0.21672

N -3.66044 1.61098 -0.12796

C -4.42448 2.74807 -0.14958

N -5.75251 2.66252 -0.05540

H -8.16615 2.13019 0.12635

N -1.55060 -4.89764 0.05954

H -1.83054 -5.87256 0.08608

N -3.79551 -4.65974 0.15702

C -2.61410 -4.03555 0.08253

N -6.07655 -4.42231 0.25359

C -4.85695 -3.85650 0.17749

H -6.15735 -5.42893 0.29124

N -2.44940 -2.71744 0.02900

C -3.59249 -2.03158 0.05476

N -4.81269 -2.51217 0.12765

H -6.89490 -3.82997 0.26817

H -1.29068 2.05054 -0.83304

H -2.63561 1.73534 -0.21180

2BH,5H

C 1.77197 4.52082 -0.28125

N 2.80713 5.32858 -0.50399

C 3.98925 4.69756 -0.49672

C 3.05141 2.70449 -0.08930

N 1.80794 3.22725 -0.06478

H 5.00871 6.42818 -0.89083

N 4.16437 3.38645 -0.29666

N 0.55639 5.20536 -0.27296

N 5.09425 5.43825 -0.70911

N 3.06680 1.35228 0.10866

H 5.99355 4.97720 -0.72952

H 2.14560 0.92894 0.16779

H 0.69213 6.19596 -0.44073

N -3.87400 6.45203 0.26189

H -4.85072 6.17488 0.25927

N -3.38500 4.21605 0.04829

C -2.98943 5.43933 0.10583

H -3.64936 7.39555 -0.02710

N -2.77248 1.98220 0.01132

C -2.43276 3.24632 -0.03677

N -1.65107 5.78117 0.03963

C -0.72949 4.74844 -0.14762

N -1.09088 3.52223 -0.20909

H -2.00957 1.31859 -0.13031

N -7.73324 1.10758 0.85455

H -8.35202 0.32418 1.03427

N -6.15004 -0.44312 0.31240

C -6.41777 0.82503 0.58018

H -7.95336 1.99041 1.29438

C -4.87658 -0.77521 0.00899

B -5.33400 1.92491 0.56317

C -4.02634 1.33029 0.17283

N -3.83900 0.00076 -0.08323

N 3.65162 -0.82638 0.23746

C 4.09873 0.43006 0.19234

N 4.30053 -3.07898 0.35977

C 4.60575 -1.74259 0.32164

N 5.35744 0.82108 0.23607

C 6.23319 -0.19354 0.33080

N 5.91876 -1.48780 0.37473

H 5.11161 -3.68375 0.42656

N 7.54089 0.13618 0.38521

H 7.80258 1.11145 0.36435

H 8.23477 -0.59312 0.46911

N -0.29795 -3.25860 -0.06918

N 0.91391 -5.26611 0.05532

C 0.88259 -3.94367 0.04198

H -0.20320 -2.25213 0.01101

N 2.22338 -7.13842 0.20280

C 2.13748 -5.79085 0.18051

H 1.37335 -7.68106 0.14178

N 1.95641 -3.13666 0.14613

C 3.10484 -3.77363 0.25833

N 3.27578 -5.09800 0.28399

H 3.12457 -7.58001 0.31439

N -4.78206 -2.16166 -0.24862

H -5.69478 -2.59961 -0.29190

N -4.16366 -4.31520 -0.54767

C -3.76242 -3.04720 -0.32268

N -3.52443 -6.50112 -0.83275

C -3.18594 -5.20822 -0.60901

H -4.49041 -6.73394 -1.01221

N -2.48856 -2.67554 -0.16679

C -1.61231 -3.67750 -0.24086

N -1.87527 -4.95176 -0.46386

H -2.78914 -7.18410 -0.94319

H -5.59143 3.07501 0.86080

H -1.34160 6.69651 0.34403

2BH,18H

C 3.33416 3.61812 -0.15245

N 4.60602 4.03718 -0.15775

C 5.50875 3.05858 -0.07492

C 3.94950 1.47253 -0.00450

N 2.95119 2.35299 -0.08119

H 7.06367 4.38755 -0.11858

N 5.24069 1.74299 0.00781

N 2.39854 4.61104 -0.25893

N 6.81263 3.41027 -0.07408

N 3.46989 0.17871 0.06086

H 7.51511 2.68850 0.00136

H 2.45589 0.14584 0.05104

H 2.77694 5.53194 -0.44836

N -1.51153 6.78418 -1.17868

H -2.52145 6.80930 -1.15607

N -1.64282 4.64072 -0.38377

C -0.87964 5.67466 -0.73064

H -0.96995 7.60571 -1.40447

N -1.65946 2.49103 0.50349

C -0.97858 3.58075 0.07160

N 0.45896 5.70733 -0.68746

C 1.00730 4.56389 -0.26990

N 0.36750 3.48440 0.13214

H -1.06269 1.71276 0.76086

N -6.41788 3.94791 1.52460

H -7.35362 3.57443 1.63342

N -5.81663 1.90106 0.70436

C -5.41184 3.09754 1.15017

H -6.17666 4.78483 2.03682

C -4.88434 1.04134 0.33400

B -3.93174 3.47649 1.20128

C -3.06389 2.38608 0.67508

N -3.57060 1.17289 0.29154

N 3.12886 -2.04049 0.13608

C 4.04789 -1.06003 0.10223

N 2.79101 -4.35340 0.22135

C 3.63316 -3.25519 0.17297

N 5.35813 -1.22724 0.10732

C 5.74502 -2.51053 0.15139

N 4.92912 -3.57099 0.18502

H 3.27495 -5.23828 0.32762

N 7.07266 -2.74593 0.16194

H 7.70852 -1.96099 0.13599

H 7.41145 -3.69697 0.19242

N -1.47439 -2.56817 -0.19363

N -1.21515 -4.86358 0.22758

C -0.65943 -3.69148 0.00561

H -0.97292 -1.68769 -0.13020

N -0.83210 -7.09175 0.61498

C -0.33039 -5.85840 0.40611

H -1.83379 -7.21276 0.66799

N 0.64049 -3.41156 -0.01220

C 1.42409 -4.46730 0.20176

N 0.99425 -5.71746 0.39715

H -0.20218 -7.85922 0.80323

N -5.43632 -0.20105 -0.09938

H -6.44968 -0.21088 -0.14976

N -5.50742 -2.39815 -0.77159

C -4.81020 -1.32821 -0.40050

N -5.42255 -4.57864 -1.46393

C -4.78530 -3.48624 -1.01633

H -6.42040 -4.54409 -1.62460

N -3.44450 -1.34180 -0.28710

C -2.81324 -2.53884 -0.45422

N -3.45063 -3.60754 -0.84799

H -4.88921 -5.41655 -1.65311

H -3.54516 4.54168 1.64115

H -3.07402 -0.33590 -0.02789

Group 2:

1BH,7H

C 0.72849 4.73579 0.30195

N 1.70104 5.72862 0.41092

C 3.02168 5.37076 0.35424

C 2.40587 3.22181 0.09214

N 1.08912 3.49386 0.14736

H 3.72729 7.30986 0.61035

N 3.39166 4.12934 0.19387

N -0.53254 5.16606 0.36639

N 3.94782 6.33328 0.46791

N 2.68458 1.91847 -0.06649

H 4.91827 6.04175 0.42791

H 1.86839 1.30876 -0.07748

H -0.71492 6.15887 0.48086

N -5.09154 5.44368 0.31393

H -5.99343 4.98756 0.27059

N -4.15177 3.37364 0.09107

C -3.98358 4.67568 0.24152

H -5.01080 6.43851 0.46274

N -3.06677 1.31422 -0.12960

C -3.03880 2.63390 0.02416

N -2.79046 5.31200 0.33024

C -1.77492 4.46143 0.26243

N -1.77540 3.17358 0.12220

H -2.16721 0.85174 -0.05568

N -7.51913 0.50724 -1.22430

H -8.16650 -0.12876 -1.67423

B -6.09956 -1.52816 -0.54948

C -6.30692 -0.04894 -0.82674

H -7.43961 1.41867 -1.66622

C -4.68724 -1.80837 -0.14184

N -5.31150 0.83570 -0.69482

C -4.14163 0.37884 -0.28170

N -3.75713 -0.85013 -0.00675

N 3.69802 -0.09673 -0.19145

C 3.89463 1.21231 -0.19307

N 4.78297 -2.17475 -0.31837

C 4.81513 -0.81500 -0.31586

N 5.03368 1.85919 -0.30933

C 6.09550 1.03670 -0.43288

N 6.04232 -0.29179 -0.43993

H 5.69587 -2.61079 -0.39284

N 7.30484 1.61921 -0.55763

H 7.36906 2.62647 -0.58790

H 8.12339 1.03939 -0.68178

N 0.31077 -3.30144 0.07074

N 1.91864 -5.00680 0.03948

C 1.60293 -3.72074 0.00126

H 0.18317 -2.30185 -0.04280

N 3.59071 -6.56792 -0.01679

C 3.22371 -5.26951 -0.04866

H 2.87245 -7.27308 0.07330

N 2.49506 -2.70892 -0.12127

C 3.74618 -3.10236 -0.19413

N 4.19696 -4.35609 -0.16645

H 4.56745 -6.81496 -0.08330

N -4.30387 -3.14778 0.15463

H -5.09336 -3.77887 0.22853

N -3.26335 -5.12530 0.53279

C -3.13723 -3.80134 0.26825

N -2.19485 -7.11768 0.93053

C -2.12767 -5.79156 0.64172

H -3.09886 -7.56814 0.93083

N -1.95087 -3.18783 0.11249

C -0.89994 -3.98523 0.23841

N -0.89028 -5.27813 0.50506

H -1.34423 -7.66037 0.89442

H -6.96759 -2.38565 -0.66952

H 1.43057 6.69896 0.52737

1BH,13H

C -4.56207 1.74338 0.31117

N -5.36950 2.81315 0.39065

C -4.75124 3.98837 0.32793

C -2.75065 3.04105 0.13230

N -3.23819 1.80708 0.18526

H -6.51195 5.01478 0.50364

N -3.42371 4.17201 0.19473

N -5.20714 0.54768 0.37298

N -5.51066 5.10303 0.40359

N -1.36050 3.04130 0.00666

H -5.06382 6.00728 0.35802

H -0.97146 2.10287 0.01086

H -6.21247 0.62250 0.48102

N -6.34901 -3.87199 0.39275

H -6.07148 -4.84331 0.36424

N -4.14089 -3.34845 0.11342

C -5.38763 -2.92410 0.28980

H -7.30295 -3.59860 0.57606

N -1.92246 -2.66869 -0.17651

C -3.22457 -2.38660 0.01127

N -5.78102 -1.64364 0.37482

C -4.76909 -0.77852 0.27627

N -3.50040 -1.05568 0.10190

H -1.30851 -1.86800 -0.09492

N -2.03886 -7.13053 -1.45658

H -1.55698 -7.89917 -1.90530

B 0.23247 -6.22781 -0.66610

C -1.25156 -6.09025 -0.98978

H -2.91882 -6.85045 -1.87936

C 0.78104 -4.92933 -0.18844

N -1.89852 -4.93386 -0.83423

C -1.22433 -3.89992 -0.34534

N 0.04864 -3.81215 -0.02207

N 0.83157 3.57633 -0.18439

C -0.44437 4.03356 -0.12652

N 3.09512 4.19761 -0.37979

C 1.72257 4.52121 -0.31372

N -0.79109 5.30866 -0.19665

C 0.22156 6.16835 -0.33409

N 1.52207 5.83079 -0.39818

H 3.65395 5.03658 -0.49520

N -0.08641 7.47543 -0.41398

H -1.05895 7.74947 -0.37234

H 0.64638 8.16189 -0.52445

N 3.23660 -0.37370 0.17362

N 5.25141 0.75850 -0.09266

C 3.90776 0.75371 -0.00568

H 2.22198 -0.29916 0.12123

N 7.16677 1.97956 -0.36259

C 5.83067 1.90878 -0.26442

H 7.67265 1.10154 -0.30859

N 3.13030 1.86921 -0.11322

C 3.72376 3.00365 -0.27428

N 5.11042 3.07756 -0.34845

H 7.67244 2.83470 -0.54970

N 2.16797 -4.85472 0.16425

H 2.60087 -5.76726 0.25945

N 4.29697 -4.24193 0.69055

C 3.03666 -3.86729 0.33852

N 6.44071 -3.60080 1.20256

C 5.16702 -3.26905 0.86184

H 6.62390 -4.55876 1.46960

N 2.69144 -2.56648 0.15906

C 3.67012 -1.72300 0.35921

N 4.91761 -1.94415 0.71921

H 7.05777 -2.86423 1.51368

H 0.88838 -7.25547 -0.80051

H 5.57803 3.96968 -0.46012

1BH,8H

C -2.50575 4.13887 -0.13361

N -2.37996 5.49278 -0.14286

C -1.16898 5.97804 -0.09016

C -0.28090 3.81550 -0.03646

N -1.46670 3.28905 -0.08866

H -1.78433 7.90403 -0.13834

N -0.06758 5.16495 -0.02888

N -3.75320 3.67905 -0.17616

N -0.96843 7.30478 -0.09323

N 0.78925 2.98908 0.01040

H -0.04648 7.71750 -0.06031

H 0.56567 1.99527 -0.00153

H -4.48403 4.38354 -0.22299

N -7.45600 0.99766 -0.31088

H -7.86308 0.07207 -0.32607

N -5.41729 -0.01701 -0.12363

C -6.11280 1.10547 -0.22078

H -8.02244 1.82568 -0.42128

N -3.26208 -0.91956 0.07105

C -4.09053 0.12006 -0.03624

N -5.59938 2.35688 -0.23791

C -4.27556 2.35113 -0.15610

N -3.46281 1.34129 -0.05825

H -2.27681 -0.68236 0.05081

N -6.19250 -4.47711 0.69730

H -6.32289 -5.39428 1.10656

B -3.71911 -5.06536 0.31450

C -4.87408 -4.09394 0.47691

H -6.78680 -3.74706 1.07927

C -2.43225 -4.33932 0.07970

N -4.68989 -2.77102 0.40214

C -3.46698 -2.33362 0.15647

N -2.34041 -3.00181 -0.00205

N 2.91057 2.21613 0.08841

C 2.14843 3.28275 0.06808

N 5.11221 1.44860 0.15895

C 4.22215 2.46634 0.14197

N 2.53585 4.55797 0.09659

C 3.88265 4.70109 0.15634

N 4.75118 3.69889 0.18003

H 6.08723 1.72784 0.19195

N 4.37164 5.95514 0.19287

H 3.75202 6.75170 0.18894

H 5.37359 6.08529 0.24414

N 2.43107 -2.31100 -0.01861

N 4.76477 -2.58315 0.00787

C 3.69097 -1.80676 0.01882

H 1.69584 -1.61481 0.03816

N 7.04961 -2.69053 0.05042

C 5.93140 -1.93731 0.05480

H 6.95645 -3.69682 0.02573

N 3.72481 -0.45239 0.07464

C 4.93219 0.05554 0.11017

N 6.08644 -0.60379 0.10495

H 7.95523 -2.24632 0.09833

N -1.24605 -5.11278 -0.08937

H -1.44284 -6.10630 -0.13514

N 0.82334 -6.00303 -0.29830

C 0.07257 -4.88193 -0.14431

N 2.92592 -6.90576 -0.48148

C 2.12839 -5.81373 -0.34651

H 2.49349 -7.79855 -0.67239

N 0.60302 -3.64797 -0.04752

C 1.92569 -3.61606 -0.11414

N 2.76079 -4.62619 -0.26377

H 3.91065 -6.76142 -0.65190

H -3.81897 -6.28554 0.38631

H 0.92695 5.46053 0.01910

1BH,14H

C 0.43853 4.80092 -0.00133

N 1.22333 5.89053 -0.00191

C 2.52911 5.64124 -0.00161

C 2.21224 3.43988 -0.00036

N 0.88353 3.54780 -0.00053

H 2.99358 7.63370 -0.00279

N 3.09312 4.42005 -0.00085

N -0.89361 5.08910 -0.00166

N 3.37164 6.69706 -0.00211

N 2.62955 2.11421 0.00037

H 4.36767 6.53099 -0.00200

H 1.85328 1.46008 0.00047

H -1.08951 6.08410 -0.00218

N -5.44660 5.12987 -0.00143

H -6.32520 4.63148 -0.00063

N -4.41185 3.09021 -0.00042

C -4.29689 4.41484 -0.00103

H -5.40855 6.13832 -0.00134

N -3.22351 1.07412 0.00056

C -3.26033 2.42291 -0.00013

N -3.14919 5.10466 -0.00135

C -2.06334 4.32493 -0.00116

N -2.03077 3.01309 -0.00060

H -2.28760 0.68771 0.00005

N -7.72478 -0.06773 0.00740

H -8.50004 -0.71243 0.00791

B -6.06449 -2.01195 0.00360

C -6.43396 -0.52626 0.00496

H -7.89138 0.93056 0.00785

C -4.58846 -2.17036 0.00125

N -5.50243 0.42825 0.00396

C -4.22501 0.06182 0.00160

N -3.70863 -1.14822 0.00022

N 3.77781 0.17151 0.00068

C 3.85688 1.51901 0.00056

N 4.98710 -1.84229 0.00077

C 4.93736 -0.43840 0.00077

N 4.98378 2.21066 0.00065

C 6.09719 1.46773 0.00080

N 6.14011 0.12494 0.00084

H 5.92936 -2.21958 0.00072

N 7.27212 2.12496 0.00095

H 7.26588 3.13586 0.00086

H 8.13849 1.60534 0.00102

N 0.52231 -2.99683 0.00000

N 2.15228 -4.64450 -0.00065

C 1.81946 -3.32134 -0.00004

H 0.29908 -2.00396 0.00059

N 3.81010 -6.26276 -0.00098

C 3.47542 -4.96452 -0.00048

H 3.11605 -6.99787 -0.00068

N 2.72258 -2.36755 0.00053

C 3.99299 -2.75849 0.00043

N 4.41350 -4.04457 0.00004

H 4.79482 -6.49966 -0.00056

N -4.04939 -3.49842 -0.00013

H -4.77330 -4.21046 -0.00041

N -2.74237 -5.37352 -0.00197

C -2.83346 -4.00985 -0.00070

N -1.41339 -7.23679 -0.00356

C -1.53740 -5.88735 -0.00247

H -2.25474 -7.79712 -0.00499

N -1.72948 -3.21411 -0.00005

C -0.60287 -3.84805 -0.00068

N -0.37504 -5.17005 -0.00187

H -0.50544 -7.67444 -0.00553

H -6.86829 -2.93763 0.00440

H 1.29081 -5.28267 -0.00130

1BH,9H

C 4.84218 -0.91832 -0.67212

N 5.67655 -1.91721 -0.86282

C 5.15222 -3.14177 -0.69745

C 3.08617 -2.39345 -0.17508

N 3.53802 -1.10833 -0.33112

H 6.92454 -4.03412 -1.15727

N 3.88014 -3.41590 -0.35931

N 5.28549 0.34280 -0.81027

N 5.96056 -4.18703 -0.88939

N 1.78377 -2.52567 0.15269

H 5.59163 -5.12292 -0.77654

H 1.18033 -1.71101 0.07598

H 6.26611 0.44871 -1.05166

N 5.33725 4.92025 -0.70080

H 4.85213 5.79556 -0.54844

N 3.37302 3.85616 -0.23252

C 4.65130 3.77368 -0.55151

H 6.31448 4.89688 -0.95464

N 1.42466 2.69317 0.24317

C 2.70922 2.70823 -0.08821

N 5.34113 2.61966 -0.75042

C 4.59134 1.55286 -0.60388

N 3.30945 1.48089 -0.28757

H 0.93199 1.81449 0.13622

N 0.88970 6.83425 2.28399

H 0.26888 7.43912 2.80788

B -1.18034 5.84524 1.12304

C 0.27719 5.81538 1.56362

H 1.73681 6.56146 2.77393

C -1.54781 4.60028 0.38201

N 1.07376 4.77845 1.28164

C 0.55587 3.80036 0.55539

N -0.66080 3.62945 0.09815

N -0.25007 -3.52256 0.30523

C 1.05317 -3.69886 0.44292

N -2.33338 -4.59727 0.46409

C -0.97645 -4.61001 0.57943

N 1.68782 -4.78154 0.82951

C 0.86165 -5.82011 1.06883

N -0.46364 -5.78822 0.95273

H -2.77334 -5.50177 0.59506

N 1.43431 -6.97591 1.45684

H 2.43358 -7.00783 1.60227

H 0.84730 -7.76290 1.69767

N -3.34592 -0.14691 -0.13817

N -5.06915 -1.72887 -0.35510

C -3.79983 -1.42760 -0.12880

H -2.36865 -0.05486 0.11494

N -6.63743 -3.39006 -0.49633

C -5.35446 -3.03239 -0.28254

H -7.31825 -2.66773 -0.68661

N -2.82641 -2.32534 0.14679

C -3.23571 -3.57234 0.17772

N -4.47748 -4.01194 -0.01779

H -6.90247 -4.36289 -0.44071

N -2.87687 4.41587 -0.10228

H -3.41767 5.26924 -0.17651

N -4.88440 3.53678 -0.73116

C -3.61419 3.31527 -0.32105

N -6.89179 2.60982 -1.34396

C -5.61007 2.44756 -0.93227

H -7.25026 3.54252 -1.48768

N -3.10569 2.08968 -0.11785

C -3.94631 1.09547 -0.35861

N -5.19807 1.17394 -0.76897

H -7.46162 1.79267 -1.50535

H -1.95829 6.75628 1.37910

H 3.00992 -0.19109 -0.19633

1BH,15H

C -0.53444 4.73299 -0.51953

N 0.07077 5.89971 -0.78994

C 1.39771 5.84889 -0.84240

C 1.42965 3.67894 -0.37028

N 0.10974 3.58405 -0.30255

H 1.53833 7.83274 -1.31546

N 2.14791 4.74765 -0.64093

N -1.88946 4.80371 -0.46177

N 2.06175 6.99079 -1.12027

N 2.05481 2.44300 -0.13962

H 3.06809 6.96867 -1.20108

H 1.38384 1.68381 -0.08350

H -2.26385 5.73851 -0.58123

N -6.32056 3.95457 0.24785

H -7.06026 3.29826 0.45512

N -4.88608 2.17307 0.24548

C -5.05620 3.48658 0.12978

H -6.49250 4.94787 0.19954

N -3.29395 0.45445 0.19156

C -3.62850 1.75293 0.11255

N -4.09078 4.39223 -0.09771

C -2.87984 3.84476 -0.21223

N -2.56891 2.57624 -0.12898

H -2.32838 0.26826 -0.04805

N -7.15138 -1.63815 1.73557

H -7.82734 -2.38046 1.60919

B -5.35655 -3.15437 0.70152

C -5.92300 -1.79905 1.11774

H -7.51956 -0.69201 1.73480

C -3.99381 -2.99118 0.12802

N -5.23951 -0.66502 0.94337

C -4.04067 -0.74428 0.38156

N -3.37855 -1.80432 -0.02940

N 3.58882 0.79616 0.27259

C 3.34451 2.12967 0.13387

N 5.21603 -0.82924 0.71692

C 4.85065 0.50818 0.55930

N 4.26378 3.06742 0.26855

C 5.49474 2.64158 0.55891

N 5.85018 1.35305 0.71756

H 6.19650 -0.97928 0.93572

N 6.45173 3.56929 0.70638

H 6.20660 4.54536 0.60270

H 7.39419 3.28896 0.94147

N 1.15373 -2.88256 -0.23429

N 3.02308 -4.19764 0.21900

C 2.44110 -3.03009 0.07326

H 0.71187 -1.97456 -0.11219

N 4.90383 -5.39222 0.69282

C 4.31997 -4.20337 0.53380

H 4.34774 -6.23050 0.57190

N 3.16854 -1.86632 0.25677

C 4.48810 -1.96327 0.56320

N 5.09744 -3.11066 0.71064

H 5.88180 -5.44560 0.94717

N -3.28081 -4.13957 -0.34840

H -3.87234 -4.94550 -0.51935

N -1.70627 -5.61962 -1.07370

C -1.99633 -4.39325 -0.56511

N -0.09756 -7.08666 -1.80143

C -0.43245 -5.87165 -1.29809

H -0.84037 -7.69554 -2.11673

N -1.03086 -3.48382 -0.26921

C 0.18028 -3.89070 -0.53952

N 0.59791 -5.02142 -1.06475

H 0.85081 -7.23341 -2.11612

H -5.94262 -4.22443 0.82198

H 2.80384 -0.90251 0.14268

3BH,14H

C -2.61635 4.09523 0.28384

N -2.49445 5.39129 -0.06734

C -1.27830 5.90683 -0.10675

C -0.17298 4.00531 0.46664

B -1.46543 3.23983 0.69005

H -1.96450 7.64485 -0.89801

N -0.11364 5.27105 0.09385

N -3.99137 3.68728 0.21643

N -1.17509 7.27426 -0.38064

N 1.02468 3.26585 0.61158

H -0.26843 7.53231 -0.75477

H 0.85846 2.29471 0.84769

H -4.67398 4.43882 0.27572

N -7.52493 0.73849 0.06182

H -7.86842 -0.17488 -0.19660

N -5.37004 -0.08203 -0.12100

C -6.19527 1.00095 -0.00784

H -8.15607 1.52933 0.04176

N -3.11117 -0.72561 -0.40717

C -4.08758 0.28241 -0.25247

N -5.80716 2.25561 0.06239

C -4.46609 2.47225 0.01184

N -3.58692 1.47134 -0.24092

H -2.15936 -0.36835 -0.47559

N -5.87503 -4.38447 0.23966

H -5.97186 -5.38345 0.37640

N -3.60308 -4.69430 0.06918

C -4.63991 -3.88978 0.07713

H -6.69277 -3.79094 0.27884

C -2.38937 -4.11494 -0.08897

N -4.50338 -2.54334 -0.07526

C -3.24423 -2.04666 -0.25528

N -2.17754 -2.81488 -0.27495

N 3.06805 2.33423 0.36681

C 2.32447 3.47198 0.30794

N 5.19873 1.39785 0.05630

C 4.33103 2.49197 0.05883

N 2.80765 4.67050 -0.00668

C 4.11517 4.69289 -0.26598

N 4.94208 3.63689 -0.25746

H 6.17193 1.65806 -0.05745

N 4.65557 5.89521 -0.57290

H 4.05936 6.71074 -0.55744

H 5.64697 5.96707 -0.74774

N 2.40725 -2.28005 0.03830

N 4.73831 -2.62009 0.07023

C 3.71249 -1.79191 0.06057

H 1.70981 -1.54214 0.05041

N 7.02611 -2.78283 0.08435

C 5.92870 -1.99491 0.07808

H 6.91059 -3.78583 0.09845

N 3.75613 -0.46077 0.06733

C 4.98567 0.04928 0.07644

N 6.11316 -0.67769 0.07861

H 7.94207 -2.35769 0.10681

N -1.35499 -4.98129 -0.05133

H -1.61754 -5.95882 0.02834

N 0.70216 -5.93608 -0.09603

C 0.03763 -4.78627 -0.06318

N 2.79053 -6.89548 -0.12149

C 2.03540 -5.78142 -0.08693

H 2.34717 -7.80252 -0.15670

N 0.54219 -3.57624 -0.01882

C 1.89518 -3.54397 -0.01131

N 2.67720 -4.60988 -0.04625

H 3.79715 -6.80266 -0.11725

H -5.25342 -1.79047 -0.04839

H -1.47102 2.09359 1.10960

3BH,8H

C -3.27348 -3.60559 0.11776

N -4.51594 -4.07233 0.20836

C -5.44581 -3.10379 0.23225

C -3.92249 -1.46481 0.08009

N -2.89709 -2.35315 0.05063

H -6.96658 -4.46681 0.37926

N -5.20293 -1.79263 0.17113

N -2.32110 -4.63653 0.08707

N -6.73549 -3.48524 0.32545

N -3.51238 -0.17557 0.00467

H -7.45218 -2.77276 0.34981

H -2.50578 -0.05943 -0.06291

H -2.74092 -5.56053 0.10108

N 1.49465 -7.11203 -0.12917

H 2.49880 -7.19483 -0.18488

N 1.70573 -4.81342 -0.07156

C 0.90311 -5.90344 -0.07166

H 0.91136 -7.93854 -0.14105

N 1.76292 -2.47167 0.00752

C 1.03623 -3.66096 -0.00743

N -0.41944 -5.86409 -0.01874

C -0.96889 -4.64015 0.03560

N -0.26702 -3.50036 0.04659

H 1.20375 -1.62077 0.05084

N 6.07507 -4.23554 -0.14553

H 7.07554 -4.07539 -0.15571

N 5.78052 -1.95891 -0.06496

C 5.27205 -3.16096 -0.10252

H 5.71971 -5.18087 -0.18050

C 4.90637 -0.91939 -0.02580

N 3.91998 -3.37307 -0.10223

C 3.10682 -2.27711 -0.02753

N 3.56830 -1.06404 0.01455

N -3.31474 2.05778 -0.09706

C -4.17445 1.05892 -0.01175

N -3.06777 4.35961 -0.22856

C -3.85969 3.28109 -0.13029

N -5.49006 1.13467 0.05175

C -5.93491 2.40965 0.03003

N -5.19048 3.50279 -0.06063

H -3.54377 5.25366 -0.15524

N -7.27956 2.57004 0.11840

H -7.86772 1.75012 0.08502

H -7.66759 3.49575 0.00603

N 1.80228 2.99715 -0.60701

N 1.04678 5.20430 -0.19478

C 0.73855 3.93831 -0.45966

H 1.45340 2.08483 -0.87883

N 0.33433 7.38393 0.15621

C 0.01758 6.03182 -0.03397

H 1.26806 7.51677 0.52951

B -0.64958 3.37620 -0.58723

C -1.65106 4.46258 -0.29225

N -1.27709 5.72821 -0.05030

H -0.38740 7.89492 0.65298

N 5.46014 0.29148 -0.00432

H 6.47208 0.31462 0.08136

N 5.66310 2.52766 0.45959

C 4.84821 1.58082 0.01579

N 5.81715 4.78368 0.88614

C 5.05962 3.74034 0.48271

H 6.77438 4.63185 1.16763

N 3.62323 1.68328 -0.39688

C 3.08982 2.94331 -0.27278

N 3.79937 3.99348 0.15608

H 5.39073 5.69913 0.93245

H 3.42625 -4.28946 -0.12451

H -0.83675 2.20674 -0.87472

2BH,7H

C -2.52488 4.11430 0.20563

N -3.80296 4.65985 0.32588

C -4.88476 3.82165 0.31962

C -3.49490 2.06751 0.08209

N -2.38307 2.82359 0.08763

H -6.27650 5.34850 0.55532

N -4.75191 2.52907 0.19741

N -1.52926 4.99846 0.21881

N -6.10748 4.35781 0.44342

N -3.25752 0.75136 -0.04353

H -6.89159 3.71491 0.44121

H -2.27075 0.50004 -0.07655

H -1.74576 5.98922 0.28214

N 2.52269 7.08075 -0.04637

H 3.53099 7.02765 -0.10717

N 2.49994 4.79623 -0.07907

C 1.82202 5.92633 -0.01304

H 2.04383 7.96663 0.01622

N 2.36876 2.47579 -0.08984

C 1.78439 3.66215 -0.03851

N 0.47490 6.03026 0.08429

C -0.10869 4.83683 0.11173

N 0.40831 3.65130 0.06394

H 1.74396 1.67765 -0.00918

N 7.47206 2.60570 -0.57294

H 8.20643 1.94893 -0.81761

N 6.17516 0.75233 -0.23922

C 6.20655 2.06961 -0.41379

H 7.52740 3.51887 -1.00510

C 4.97696 0.17721 -0.04858

B 4.94198 2.93912 -0.41323

C 3.74277 2.08684 -0.17411

N 3.80199 0.73614 -0.00230

N -3.42592 -1.49926 -0.13419

C -4.10854 -0.36455 -0.12191

N -3.63396 -3.83494 -0.23232

C -4.18632 -2.59112 -0.21605

N -5.41274 -0.20661 -0.18628

C -6.08200 -1.37426 -0.26906

N -5.52393 -2.58122 -0.28612

H -4.31032 -4.58869 -0.29025

N -7.42628 -1.30139 -0.33928

H -7.87357 -0.39623 -0.35290

H -7.96498 -2.15233 -0.42438

N 0.93225 -3.14148 0.05995

N 0.11344 -5.33730 -0.02025

C -0.09784 -4.03012 -0.01275

H 0.65684 -2.16741 -0.00325

N -0.82049 -7.42418 -0.11622

C -0.98887 -6.08525 -0.10326

H 0.11893 -7.79537 -0.08388

N -1.31448 -3.44238 -0.08467

C -2.31669 -4.28927 -0.15875

N -2.24289 -5.61988 -0.17369

H -1.62418 -8.02975 -0.19972

N 5.13364 -1.22429 0.13404

H 6.11412 -1.47798 0.17636

N 4.94452 -3.46390 0.37746

C 4.30824 -2.28217 0.20227

N 4.74139 -5.73888 0.60558

C 4.15724 -4.52415 0.44151

H 5.73308 -5.77914 0.79320

N 2.97634 -2.16138 0.09555

C 2.31522 -3.31022 0.17336

N 2.81481 -4.51885 0.35002

H 4.14815 -6.53863 0.77291

H 4.99667 4.13864 -0.60564

H -3.92288 5.66214 0.42197

2BH,9H

C -4.92098 0.54070 0.65012

N -6.00427 -0.17857 0.85600

C -5.85025 -1.50250 0.70980

C -3.66109 -1.37748 0.16674

N -3.72403 -0.01578 0.31295

H -7.79757 -1.85134 1.19516

N -4.71103 -2.12981 0.36900

N -4.99073 1.87491 0.76473

N -6.91904 -2.27438 0.92405

N -2.45729 -1.88623 -0.17139

H -6.83070 -3.27777 0.82429

H -1.63776 -1.29008 -0.09006

H -5.90304 2.25711 0.99424

N -3.83533 6.29027 0.48249

H -3.14182 7.00267 0.29441

N -2.21525 4.72953 0.09008

C -3.46853 4.99819 0.39283

H -4.79033 6.53037 0.70581

N -0.62072 3.09595 -0.27460

C -1.86412 3.44013 0.01516

N -4.43685 4.07458 0.62914

C -3.99046 2.84315 0.52999

N -2.77309 2.41926 0.24207

H -0.37707 2.11634 -0.15993

N 2.23619 7.03355 -1.86038

H 3.16253 7.40534 -1.67809

N 2.99121 5.12085 -0.86259

C 1.93983 5.80778 -1.29869

H 1.48345 7.70370 -1.94127

C 2.77676 3.89854 -0.35102

B 0.50541 5.26765 -1.20657

C 0.51668 3.93046 -0.55884

N 1.64367 3.27985 -0.16831

N -0.83165 -3.46922 -0.28715

C -2.11927 -3.22610 -0.46151

N 0.81858 -5.13448 -0.42051

C -0.47383 -4.72576 -0.56453

N -3.04773 -4.05271 -0.88608

C -2.58213 -5.29610 -1.11888

N -1.31691 -5.68227 -0.96814

H 0.96485 -6.12806 -0.56204

N -3.47604 -6.21203 -1.53927

H -4.42880 -5.92639 -1.71687

H -3.15668 -7.13966 -1.78304

N 3.08890 -1.18904 0.22680

N 4.27855 -3.20894 0.41716

C 3.15230 -2.54809 0.20145

H 2.17348 -0.81776 -0.00151

N 5.29015 -5.25909 0.53496

C 4.16829 -4.53883 0.33041

H 6.15221 -4.77090 0.73481

N 1.95893 -3.11693 -0.07435

C 1.98333 -4.42887 -0.12393

N 3.04244 -5.21505 0.05963

H 5.25669 -6.26661 0.47489

N 4.00290 3.30328 0.05618

H 4.76749 3.96824 0.05785

N 5.68351 1.87474 0.60699

C 4.38157 2.03328 0.28264

N 7.35528 0.40275 1.15847

C 6.06319 0.62213 0.81252

H 7.99642 1.18240 1.18488

N 3.51425 1.01990 0.16739

C 4.03740 -0.17969 0.38559

N 5.28094 -0.47087 0.71552

H 7.67939 -0.54850 1.25406

H -0.45408 5.88783 -1.62993

H -2.95677 0.71092 0.17028

2BH,13H

C 3.09165 3.81884 -0.26876

N 4.35642 4.25559 -0.39047

C 5.28316 3.30366 -0.38006

C 3.77070 1.69314 -0.14740

N 2.74679 2.53777 -0.14660

H 6.79684 4.66157 -0.60307

N 5.05121 1.98251 -0.25886

N 2.15718 4.80385 -0.27330

N 6.57490 3.68149 -0.50046

N 3.34801 0.36839 -0.01896

H 7.29621 2.97510 -0.50419

H 2.33603 0.28678 0.01822

H 2.54218 5.73898 -0.34657

N -1.62952 7.32893 0.02528

H -2.62265 7.38727 0.20440

N -1.86285 5.05456 0.10299

C -1.05381 6.10185 0.00802

H -1.04274 8.14979 0.05013

N -1.97856 2.73314 0.15373

C -1.26380 3.86181 0.06530

N 0.28161 6.05585 -0.11136

C 0.76118 4.80856 -0.14255

N 0.08446 3.69051 -0.06929

H -1.43661 1.88373 0.03403

N -6.98602 3.45358 1.02666

H -7.84925 3.00979 0.72881

N -5.97975 1.50228 0.37155

C -5.81805 2.78877 0.68340

H -6.96355 4.46251 0.94939

C -4.87821 0.80427 0.08101

B -4.44965 3.47183 0.68568

C -3.37913 2.50375 0.28878

N -3.63674 1.19735 0.01398

N 3.19351 -1.88180 0.17628

C 4.01660 -0.80692 0.08380

N 3.09591 -4.22497 0.37777

C 3.82473 -3.01910 0.27622

N 5.33863 -0.86954 0.09699

C 5.85137 -2.09689 0.21207

N 5.13430 -3.23174 0.30548

H 3.72355 -5.01473 0.48711

N 7.19202 -2.20512 0.23545

H 7.74897 -1.36359 0.16950

H 7.62483 -3.11348 0.32468

N -1.29233 -2.94663 -0.18489

N -0.84804 -5.20253 0.17682

C -0.43040 -3.93168 0.02840

H -0.90143 -2.00584 -0.17497

N -0.28857 -7.38970 0.54576

C 0.06380 -6.10586 0.38327

H -1.28155 -7.59594 0.51149

N 0.87234 -3.54082 0.10089

C 1.76533 -4.45228 0.29851

N 1.39980 -5.78618 0.44102

H 0.36663 -8.13077 0.75465

N -5.22706 -0.55073 -0.22233

H -6.23409 -0.65613 -0.29255

N -5.30369 -2.77866 -0.65932

C -4.55376 -1.68688 -0.36127

N -5.35953 -5.03265 -1.08528

C -4.64815 -3.91388 -0.79377

H -6.33549 -4.92811 -1.32769

N -3.20970 -1.74573 -0.20185

C -2.70905 -2.94632 -0.35445

N -3.31040 -4.07733 -0.65733

H -4.85691 -5.86840 -1.34709

H -4.32787 4.64393 0.98291

H 2.09971 -6.50245 0.59695

2BH,14H

C -2.21141 -4.33129 0.01874

N -3.34368 -5.05436 0.02746

C -4.46530 -4.34167 0.02602

C -3.35967 -2.41306 0.00863

N -2.16483 -3.00154 0.00942

H -5.63133 -6.02269 0.04147

N -4.54031 -2.99820 0.01651

N -1.07952 -5.08700 0.01957

N -5.63788 -5.01268 0.03489

N -3.25709 -1.02583 -0.00152

H -6.50265 -4.49141 0.03313

H -2.29314 -0.70747 -0.00541

H -1.26071 -6.08476 0.02425

N 3.13703 -6.80768 0.00141

H 4.13603 -6.66851 -0.06188

N 2.92610 -4.52983 -0.00616

C 2.33276 -5.71800 -0.00198

H 2.72747 -7.72816 -0.05798

N 2.59619 -2.23208 -0.00858

C 2.10366 -3.48042 -0.00232

N 1.01065 -5.93254 0.00465

C 0.29099 -4.80471 0.01026

N 0.74159 -3.57340 0.00787

H 1.89044 -1.50336 -0.00091

N 7.72880 -1.95147 -0.11623

H 8.43672 -1.27841 0.15928

N 6.26702 -0.18900 -0.03192

C 6.41594 -1.51472 -0.05887

H 7.89654 -2.90913 0.16447

C 5.01461 0.27218 -0.01591

B 5.22882 -2.48167 -0.05329

C 3.92945 -1.73159 -0.02441

N 3.88270 -0.37452 -0.01117

N -3.60157 1.20407 -0.01580

C -4.17497 -0.01896 -0.00786

N -3.97717 3.52263 -0.03071

C -4.45257 2.19917 -0.02132

N -5.47858 -0.24180 -0.00671

C -6.23679 0.86075 -0.01462

N -5.77804 2.12408 -0.02191

H -4.71284 4.22185 -0.05065

N -7.57156 0.68682 -0.01503

H -7.94096 -0.25424 -0.00995

H -8.18331 1.49076 -0.02099

N 0.59760 2.93997 0.05364

N -0.30854 5.07579 -0.00293

C -0.48842 3.72346 0.02271

H 0.43368 1.93528 0.05775

N -1.24885 7.19273 -0.07167

C -1.41942 5.86358 -0.04221

H -0.33219 7.61883 -0.09211

N -1.67884 3.17233 0.01583

C -2.71633 4.00508 -0.02057

N -2.62996 5.35524 -0.05020

H -2.07566 7.77663 -0.11148

N 5.02452 1.70612 0.00177

H 5.97709 2.05925 -0.00704

N 4.51192 3.92904 0.03782

C 4.08666 2.63529 0.02377

N 3.97172 6.15452 0.04800

C 3.58349 4.85539 0.05288

H 4.95983 6.34947 0.14494

N 2.77047 2.30247 0.03455

C 1.95682 3.31093 0.04903

N 2.24036 4.61984 0.05848

H 3.29932 6.88474 0.22899

H 5.40392 -3.68248 -0.06262

H 0.71895 5.35755 0.01220

2BH,15H

C -1.86367 4.42213 0.48666

N -2.95467 5.14458 0.78757

C -4.08302 4.45071 0.88724

C -3.06402 2.54046 0.39592

N -1.87059 3.10208 0.28118

H -5.16012 6.11435 1.38840

N -4.20410 3.12089 0.70350

N -0.72187 5.14523 0.37910

N -5.21180 5.12336 1.19809

N -3.01257 1.15404 0.17194

H -6.07278 4.61040 1.32165

H -2.05777 0.81977 0.09127

H -0.85097 6.14644 0.47491

N 3.50754 6.58424 -0.56774

H 4.47043 6.37523 -0.79244

N 3.16149 4.32831 -0.41941

C 2.65541 5.55415 -0.35527

H 3.15868 7.53107 -0.55966

N 2.68118 2.06030 -0.20176

C 2.29382 3.33959 -0.19051

N 1.37442 5.86558 -0.09690

C 0.60743 4.79102 0.10153

N 0.96928 3.53467 0.08018

H 1.95060 1.40562 0.05669

N 7.63813 1.23748 -1.25803

H 8.35047 0.58237 -0.95159

N 6.14366 -0.28562 -0.42553

C 6.34564 0.96773 -0.83760

H 7.91166 2.21169 -1.27265

C 4.90317 -0.60696 -0.05144

B 5.23445 2.02121 -0.86530

C 3.95166 1.43711 -0.36689

N 3.83539 0.13729 0.01304

N -3.55834 -1.02767 -0.24650

C -3.99083 0.25581 -0.09102

N -4.19198 -3.23392 -0.70855

C -4.52422 -1.88972 -0.53004

N -5.25282 0.62772 -0.20306

C -6.12478 -0.34037 -0.49021

N -5.81026 -1.63760 -0.66844

H -4.97594 -3.84101 -0.92898

N -7.41457 0.00481 -0.61388

H -7.67415 0.97563 -0.49645

H -8.10455 -0.69645 -0.84676

N 0.35916 -3.04351 0.22382

N -0.62507 -5.09824 -0.26702

C -0.69211 -3.79915 -0.09654

H 0.29717 -2.03297 0.12443

N -1.67765 -7.05396 -0.77433

C -1.75398 -5.73538 -0.58832

H -0.78027 -7.51231 -0.66900

N -1.89656 -3.13939 -0.26375

C -3.00079 -3.86687 -0.57544

N -2.96860 -5.16328 -0.74554

H -2.50424 -7.57663 -1.03449

N 4.83996 -1.97135 0.37564

H 5.76403 -2.36595 0.51654

N 4.19369 -4.05284 1.02743

C 3.84198 -2.82822 0.56489

N 3.51068 -6.14151 1.68904

C 3.20623 -4.90513 1.22498

H 4.45994 -6.32617 1.98326

N 2.55794 -2.49259 0.28780

C 1.69917 -3.45200 0.51917

N 1.89290 -4.66012 0.99843

H 2.75828 -6.75526 1.96649

H 5.44102 3.15699 -1.24602

H -2.05380 -2.12125 -0.13597

3BH,7H

C 4.43114 2.38406 0.06635

N 5.06855 3.44135 0.54860

C 4.25657 4.52750 0.60588

C 2.46511 3.41702 -0.20727

N 3.21312 2.26791 -0.35030

H 5.77211 5.68088 1.36888

N 2.97903 4.56181 0.26337

N 5.28612 1.23728 -0.00819

N 4.81187 5.67274 1.05865

N 1.19789 3.26604 -0.56949

H 4.22397 6.49141 1.14037

H 1.00426 2.31808 -0.87441

H 6.25773 1.49968 0.12816

N 6.83881 -3.09486 -0.51641

H 6.63095 -4.08428 -0.59355

N 4.58779 -2.75503 -0.27465

C 5.79637 -2.26917 -0.34469

H 7.79713 -2.78045 -0.58942

N 2.31531 -2.28313 -0.01004

C 3.59217 -1.86819 -0.10479

N 6.03960 -0.92531 -0.25011

C 4.96283 -0.05626 -0.09013

N 3.75142 -0.53479 -0.01572

H 1.62613 -1.53625 0.06445

N 2.59295 -6.92313 0.08994

H 2.12526 -7.81759 0.14182

N 0.53671 -5.90895 0.12794

C 1.85864 -5.79226 0.08286

H 3.60043 -6.86043 0.07184

C -0.14236 -4.75292 0.11939

N 2.53329 -4.62767 0.03117

C 1.74840 -3.57224 0.02534

N 0.42268 -3.54377 0.06783

B -1.27711 3.26382 -0.65541

C -0.00438 4.02829 -0.44613

N -3.82411 3.81698 -0.31706

C -2.44785 4.16353 -0.34399

N 0.09223 5.31663 -0.12397

C -1.05820 5.96530 0.01941

N -2.28900 5.46026 -0.04915

H -4.45595 4.60873 -0.24836

N -0.97015 7.34420 0.25688

H -0.08269 7.61236 0.66850

H -1.77996 7.71472 0.74262

N -3.41656 -0.71802 -0.00377

N -5.60444 0.20032 -0.02070

C -4.29529 0.36862 -0.05425

H -2.44634 -0.42354 -0.05851

N -7.63260 1.27280 -0.01312

C -6.27991 1.36667 -0.07387

H -8.05435 0.35599 -0.04541

N -3.63968 1.51338 -0.13898

C -4.40433 2.60875 -0.21069

N -5.75302 2.58035 -0.17165

H -8.18488 2.10645 -0.15482

N -1.49237 -4.92260 0.17394

H -1.77079 -5.89706 0.22393

N -3.74219 -4.72381 0.25838

C -2.58876 -4.05956 0.17185

N -6.02987 -4.52018 0.34314

C -4.81993 -3.92889 0.25559

H -6.09279 -5.52551 0.41297

N -2.43021 -2.75907 0.08735

C -3.59255 -2.06181 0.08952

N -4.80114 -2.59673 0.17271

H -6.85574 -3.93776 0.34766

H -1.26854 2.09783 -1.00611

H 6.97933 -0.55793 -0.35198

3BH,9H

C 4.97064 1.39406 0.19167

N 5.80498 2.40529 0.27225

C 5.18367 3.60448 0.11006

C 3.13284 2.68720 -0.14911

N 3.67091 1.41625 -0.02272

H 6.96447 4.59504 0.30950

N 3.89047 3.78652 -0.08820

N 5.57634 0.13323 0.37287

N 5.97048 4.69530 0.16228

N 1.82149 2.71522 -0.32278

H 5.54373 5.60453 0.04135

H 1.39510 1.79611 -0.34006

H 6.57767 0.15621 0.53945

N 5.82878 -4.41335 0.84429

H 5.36471 -5.31276 0.86058

N 3.79323 -3.48767 0.39408

C 5.10886 -3.31412 0.60384

H 6.82111 -4.33389 1.02655

N 1.75135 -2.46856 -0.07799

C 3.08204 -2.41506 0.16138

N 5.75702 -2.13975 0.59864

C 5.00281 -1.08259 0.38623

N 3.65945 -1.17117 0.16965

H 1.20276 -1.61552 -0.00497

N 1.24880 -6.91131 -1.31760

H 0.63939 -7.69370 -1.51365

N -0.59684 -5.70785 -0.67367

C 0.71317 -5.74802 -0.89810

H 2.22984 -6.94432 -1.55686

C -1.07572 -4.52046 -0.28358

N 1.55489 -4.70950 -0.74146

C 0.96330 -3.62070 -0.30404

N -0.32633 -3.43316 -0.06870

B -0.59599 3.23157 -0.35920

C 0.81290 3.73388 -0.36645

N -2.95821 4.40790 -0.20494

C -1.54442 4.41236 -0.30046

N 1.19991 5.00780 -0.37622

C 0.21975 5.90294 -0.37893

N -1.09247 5.67176 -0.32338

H -3.38113 5.33073 -0.21791

N 0.60144 7.24588 -0.46915

H 1.54609 7.41308 -0.14090

H -0.09144 7.88619 -0.09746

N -3.56744 -0.07001 0.21633

N -5.50702 1.30533 0.28410

C -4.19818 1.17341 0.18415

H -2.56369 0.02436 0.09605

N -7.23694 2.81092 0.32287

C -5.90388 2.59459 0.21392

H -7.85649 2.01719 0.39535

N -3.30898 2.14049 0.03479

C -3.80611 3.37574 -0.03572

N -5.12111 3.65667 0.05300

H -7.59266 3.75114 0.22791

N -2.42580 -4.51700 -0.08691

H -2.85386 -5.43023 -0.19702

N -4.60066 -3.95893 0.25426

C -3.35744 -3.50006 0.11096

N -6.79948 -3.37075 0.56996

C -5.51547 -2.99130 0.40659

H -7.03530 -4.35266 0.57159

N -2.97792 -2.24374 0.10889

C -3.99166 -1.35973 0.25280

N -5.26692 -1.67731 0.40902

H -7.50748 -2.65803 0.68044

H -0.82045 2.03426 -0.37677

H 3.21105 -0.21481 0.00643

3BH,13H

C 4.69370 1.97859 0.04262

N 5.43548 3.04812 0.33987

C 4.72729 4.18960 0.34937

C 2.80088 3.16973 -0.18231

N 3.41911 1.95753 -0.24330

H 6.40155 5.29124 0.78092

N 3.42222 4.31089 0.11167

N 5.42671 0.78673 0.03928

N 5.40330 5.32717 0.63717

N 1.48548 3.09259 -0.45333

H 4.90319 6.20466 0.60883

H 1.21437 2.13670 -0.65200

H 6.42608 0.92274 0.14053

N 6.67389 -3.60160 -0.11570

H 6.42354 -4.57817 -0.16938

N 4.42872 -3.12946 -0.09500

C 5.69398 -2.67163 -0.08379

H 7.63902 -3.30391 -0.13225

N 2.16121 -2.47356 -0.03891

C 3.52753 -2.16836 -0.05300

N 6.05643 -1.39337 -0.04281

C 5.03940 -0.51823 -0.01379

N 3.75063 -0.86011 -0.01273

H 1.57264 -1.64602 0.00003

N 2.00573 -7.09410 -0.14719

H 1.46697 -7.94852 -0.14742

N 0.04005 -5.90512 -0.02279

C 1.38181 -5.90339 -0.08879

H 3.01533 -7.11132 -0.20159

C -0.48184 -4.68416 0.02729

N 2.14521 -4.81046 -0.10057

C 1.49646 -3.65850 -0.04139

N 0.14310 -3.53683 0.02284

B -0.98745 3.30732 -0.52611

C 0.36868 3.95901 -0.35408

N -3.47436 4.11590 -0.22729

C -2.05783 4.31678 -0.25966

N 0.56786 5.23983 -0.08655

C -0.52505 6.00127 0.04108

N -1.78904 5.61418 -0.00699

H -4.01170 4.97790 -0.18970

N -0.29021 7.36898 0.23182

H 0.62679 7.55477 0.62368

H -1.05018 7.84350 0.70704

N -3.47094 -0.42274 -0.00646

N -5.59322 0.67704 -0.03190

C -4.27956 0.75491 -0.03702

H -2.48069 -0.18320 -0.06843

N -7.51810 1.93558 -0.02009

C -6.16273 1.91077 -0.06103

H -8.02523 1.06562 -0.08689

N -3.51654 1.81237 -0.07943

C -4.17363 2.99077 -0.14250

N -5.52618 3.06742 -0.11858

H -7.98991 2.82134 -0.13835

N -1.89177 -4.75251 0.10369

H -2.17923 -5.72574 0.12428

N -4.14480 -4.31361 0.23593

C -2.85413 -3.80324 0.12609

N -6.43880 -3.92811 0.37754

C -5.19794 -3.43143 0.26486

H -6.64606 -4.91514 0.44615

N -2.65164 -2.53112 0.05095

C -3.74575 -1.71362 0.07955

N -5.01587 -2.14690 0.18681

H -7.19756 -3.25492 0.39975

H -1.08268 2.12762 -0.81947

H -4.30238 -5.31207 0.30813

3BH,15H

C 4.98173 0.83008 0.02536

N 5.94661 1.69983 0.32906

C 5.56489 2.97898 0.17421

C 3.49079 2.43035 -0.48302

N 3.78046 1.10170 -0.40916

H 7.41349 3.66009 0.74197

N 4.35909 3.40064 -0.20468

N 5.36293 -0.50302 0.21932

N 6.49085 3.93049 0.43519

N 2.21928 2.66277 -0.85859

H 6.22584 4.90100 0.34172

H 1.72770 1.78827 -1.00031

H 6.34065 -0.61424 0.46375

N 5.34217 -5.00173 0.97538

H 4.82876 -5.86065 1.11079

N 3.33383 -3.98388 0.52969

C 4.66608 -3.86970 0.68307

H 6.33672 -4.95327 1.14690

N 1.34934 -2.80227 0.05742

C 2.74547 -2.84369 0.23266

N 5.36189 -2.74297 0.57306

C 4.63565 -1.65070 0.29226

N 3.31184 -1.65274 0.10762

H 0.98265 -1.85608 0.03448

N 0.40302 -7.23060 -0.78871

H -0.27056 -7.96596 -0.95426

N -1.34283 -5.75901 -0.53475

C -0.01648 -5.97699 -0.55971

H 1.39726 -7.41432 -0.82260

C -1.66557 -4.49544 -0.33516

N 0.91123 -5.03912 -0.37622

C 0.47018 -3.81337 -0.15803

N -0.85072 -3.46636 -0.14842

B -0.14571 3.40478 -0.68515

C 1.32560 3.75729 -0.72035

N -2.33929 4.74583 -0.10592

C -0.93668 4.63183 -0.35952

N 1.82246 4.97567 -0.58216

C 0.93926 5.96077 -0.38438

N -0.37139 5.85066 -0.24227

H -2.65903 5.70602 -0.00990

N 1.47339 7.25335 -0.33710

H 2.45438 7.26260 -0.07977

H 0.89800 7.90876 0.18025

N -3.33979 0.31864 0.25960

N -5.10899 1.88713 0.59898

C -3.84490 1.65894 0.32868

H -2.32803 0.34797 0.14038

N -6.66356 3.55469 0.89286

C -5.37947 3.21953 0.62223

H -7.31533 2.83017 1.15570

N -2.87937 2.50758 0.10309

C -3.24762 3.80767 0.13209

N -4.51684 4.19560 0.40117

H -6.90010 4.53138 0.99850

N -3.04745 -4.29231 -0.31883

H -3.59006 -5.14163 -0.44728

N -5.09625 -3.31467 -0.15621

C -3.79450 -3.17743 -0.14998

N -7.13093 -2.28093 0.01934

C -5.80118 -2.17484 0.00870

H -7.56710 -3.18607 -0.10115

N -3.18408 -1.97403 0.01134

C -3.97825 -0.84294 0.15009

N -5.28543 -0.95280 0.15714

H -7.68768 -1.44191 0.13022

H -0.51821 2.25595 -0.86482

H -2.15095 -1.98100 0.01531

2BH,8H

C 1.19924 4.72440 -0.01993

N 2.25017 5.58889 -0.02636

C 3.44752 5.07042 -0.02129

C 2.53814 2.91609 -0.00558

N 1.32882 3.38645 -0.01030

H 4.38168 6.86440 -0.03552

N 3.64706 3.71446 -0.01008

N -0.00394 5.28655 -0.02406

N 4.53136 5.86229 -0.02672

N 2.70719 1.57268 0.00393

H 5.47381 5.49759 -0.02488

H 1.84376 1.03203 0.00579

H -0.01771 6.30269 -0.03266

N -4.50391 6.08810 -0.02777

H -5.45359 5.74086 -0.04175

N -3.81379 3.90944 -0.00308

C -3.49266 5.19200 -0.01957

H -4.30164 7.07616 -0.06195

N -2.99322 1.72967 0.01901

C -2.79251 3.04199 0.00469

N -2.23576 5.69109 -0.02703

C -1.31955 4.72937 -0.01713

N -1.47588 3.44003 -0.00240

H -2.14820 1.16536 0.01422

N -7.91450 0.29307 0.04958

H -8.43752 -0.53714 0.30971

N -6.08764 -1.08240 0.03665

C -6.53621 0.16903 0.05529

H -8.29152 1.17605 0.36883

C -4.75879 -1.26862 0.00586

B -5.60235 1.38460 0.05766

C -4.18093 0.93425 0.02528

N -3.80803 -0.37809 -0.00146

N 3.65457 -0.47802 0.01656

C 3.87347 0.81530 0.01024

N 4.66080 -2.58213 0.02733

C 4.75665 -1.23229 0.02214

N 5.05271 1.43711 0.00949

C 6.10389 0.58168 0.01711

N 6.00502 -0.74134 0.02338

H 5.54593 -3.07826 0.03324

N 7.33916 1.11752 0.01816

H 7.46801 2.11846 0.01498

H 8.13860 0.49761 0.02435

N 0.09889 -3.31115 -0.00672

N 1.54329 -5.16560 0.01125

C 1.34433 -3.85606 0.00476

H 0.08213 -2.29705 0.00004

N 3.07158 -6.86829 0.03252

C 2.82362 -5.54321 0.02450

H 2.28936 -7.50852 0.03734

N 2.33244 -2.92959 0.01056

C 3.54317 -3.43211 0.02239

N 3.88264 -4.71746 0.03005

H 4.02482 -7.20121 0.05092

N -4.47747 -2.66497 -0.01933

H -5.34157 -3.19567 -0.02805

N -3.66286 -4.77080 -0.05584

C -3.38615 -3.44464 -0.02749

N -2.83200 -6.90983 -0.07594

C -2.61167 -5.57069 -0.06308

H -3.77782 -7.24654 -0.18649

N -2.13818 -2.94732 -0.00915

C -1.18467 -3.87026 -0.02215

N -1.32219 -5.18138 -0.04997

H -2.04209 -7.52841 -0.18818

H -6.03834 2.51852 0.09421

H 4.55811 3.21706 -0.00473

2BH,12H

C 1.50385 4.54878 -0.53982

N 2.54262 5.31957 -0.90021

C 3.70633 4.69341 -1.02598

C 2.81803 2.75170 -0.44052

N 1.60294 3.23387 -0.29164

H 4.65843 6.39790 -1.62425

N 3.91191 3.37488 -0.81233

N 0.32568 5.19459 -0.41426

N 4.78002 5.42043 -1.39711

N 2.86027 1.35602 -0.17882

H 5.66128 4.95415 -1.55833

H 1.92222 0.96718 -0.11291

H 0.37718 6.20008 -0.53774

N -3.98412 6.31104 0.61981

H -4.94071 6.03018 0.78703

N -3.47234 4.08892 0.44987

C -3.06323 5.34866 0.38121

H -3.72665 7.28255 0.52885

N -2.82702 1.86421 0.20848

C -2.53821 3.16577 0.20192

N -1.81204 5.75829 0.10072

C -0.97405 4.74345 -0.11007

N -1.23428 3.46447 -0.08713

H -2.05415 1.26474 -0.06217

N -7.71133 0.65666 1.22358

H -8.22775 -0.19009 1.44021

N -6.08017 -0.75656 0.45895

C -6.38675 0.47923 0.85647

H -7.91077 1.45724 1.80976

C -4.82291 -0.97495 0.06191

B -5.36686 1.62230 0.87942

C -4.04592 1.14247 0.37550

N -3.82438 -0.14145 -0.01379

N 3.64529 -0.76082 0.29267

C 3.90809 0.58373 0.14279

N 4.42336 -2.91182 0.74956

C 4.67098 -1.59089 0.62128

N 5.11695 1.05064 0.32728

C 6.06914 0.16688 0.65805

N 5.89423 -1.16014 0.81206

H 5.22870 -3.48454 0.98461

N 7.29773 0.64655 0.85516

H 7.46001 1.64105 0.75288

H 8.04739 0.01985 1.11960

N -0.10173 -3.07863 -0.24566

N 1.05197 -5.05484 0.17485

C 1.03368 -3.73723 0.05363

H -0.09825 -2.07202 -0.11963

N 2.26446 -6.93939 0.62163

C 2.22113 -5.60551 0.48852

H 1.41241 -7.46933 0.48949

N 2.13697 -2.94920 0.24149

C 3.23389 -3.62543 0.54781

N 3.37071 -4.92601 0.68811

H 3.12911 -7.39419 0.88076

N -4.65202 -2.32791 -0.36423

H -5.54085 -2.79805 -0.49608

N -3.86110 -4.35535 -1.01259

C -3.58784 -3.10841 -0.55974

N -3.03618 -6.39430 -1.66873

C -2.81455 -5.13518 -1.21353

H -3.97020 -6.64157 -1.96512

N -2.33435 -2.67867 -0.29749

C -1.39947 -3.57482 -0.52797

N -1.52406 -4.79807 -1.00015

H -2.24263 -6.94974 -1.95442

H -5.65916 2.73348 1.27850

H 2.72566 -1.23940 0.15271

Group 3:

1BH,10H

C 2.93231 3.87820 -0.15694

N 2.94139 5.22408 -0.15239

C 1.75900 5.81521 -0.09146

C 0.70467 3.83961 -0.05660

N 1.80818 3.14612 -0.11771

H 2.60485 7.66713 -0.12937

N 0.56708 5.16634 -0.03380

N 4.13159 3.27818 -0.20827

N 1.72913 7.16268 -0.08383

N -0.44421 3.04289 -0.01335

H 0.85280 7.66129 -0.04777

H -0.26441 2.04024 -0.03880

H 4.93088 3.90179 -0.25702

N 7.51077 0.20224 -0.36835

H 7.81009 -0.76322 -0.39024

N 5.37431 -0.57925 -0.15269

C 6.18795 0.46145 -0.26431

H 8.16278 0.96110 -0.50078

N 3.13592 -1.23505 0.07672

C 4.07493 -0.29047 -0.04909

N 5.81794 1.75859 -0.28304

C 4.50087 1.90985 -0.18692

N 3.58517 0.98847 -0.07239

H 2.18397 -0.88953 0.05204

N 5.66988 -5.06049 0.82221

H 5.69766 -5.98118 1.24303

B 3.16305 -5.40609 0.36753

C 4.40387 -4.55126 0.55735

H 6.32396 -4.38748 1.21122

C 1.96540 -4.55662 0.09004

N 4.35430 -3.21823 0.46712

C 3.19000 -2.66080 0.17775

N 2.00804 -3.21673 -0.00834

N -2.64851 2.46654 0.08989

C -1.73897 3.40124 0.06361

N -4.91497 1.97037 0.18827

C -3.92072 2.86882 0.16664

N -2.04845 4.73041 0.11193

C -3.37046 5.07123 0.19527

N -4.31477 4.16478 0.22362

H -5.85462 2.35396 0.22848

N -3.68221 6.37360 0.24714

H -2.98136 7.10213 0.23528

H -4.66245 6.62258 0.31132

N -2.66307 -2.05926 0.00498

N -5.01330 -2.07601 -0.00470

C -3.85787 -1.42632 0.03255

H -1.85630 -1.44800 0.07539

N -7.29638 -1.93185 -0.00107

C -6.10297 -1.30870 0.03003

H -7.31505 -2.94140 -0.05529

N -3.74751 -0.07337 0.10734

C -4.89162 0.55571 0.12606

N -6.11137 0.03694 0.09324

H -8.14878 -1.39055 0.02003

N 0.71103 -5.21077 -0.10912

H 0.81264 -6.21812 -0.16512

N -1.43767 -5.89008 -0.33567

C -0.57584 -4.85306 -0.16586

N -3.61644 -6.57041 -0.57491

C -2.71621 -5.57046 -0.38624

H -3.29033 -7.52612 -0.54827

N -0.98310 -3.57255 -0.05156

C -2.29355 -3.41106 -0.11513

N -3.22845 -4.32638 -0.27856

H -4.59926 -6.35553 -0.49082

H 3.13753 -6.62967 0.45158

H -1.22499 5.37302 0.07837

1BH,11H

C -3.04649 -3.78723 -0.39878

N -4.31815 -4.20850 -0.49967

C -5.24257 -3.25793 -0.44264

C -3.71638 -1.66824 -0.20895

N -2.69713 -2.50205 -0.25618

H -6.75997 -4.60402 -0.68676

N -5.00063 -1.93716 -0.29273

N -2.11569 -4.76427 -0.45356

N -6.53624 -3.62936 -0.54000

N -3.27754 -0.32979 -0.07483

H -7.25689 -2.92240 -0.53982

H -2.26413 -0.24623 -0.14560

H -2.49262 -5.69894 -0.56944

N 1.73035 -7.22403 -0.39580

H 2.73918 -7.26325 -0.34506

N 1.91417 -4.96247 -0.12123

C 1.12914 -6.01502 -0.30774

H 1.17701 -8.04682 -0.58324

N 1.95812 -2.64339 0.15926

C 1.28845 -3.78712 -0.03628

N -0.21192 -5.99077 -0.41704

C -0.71252 -4.75910 -0.33284

N -0.06544 -3.63776 -0.15400

H 1.39750 -1.80531 0.06598

N 6.14064 -4.13599 1.53480

H 6.99954 -3.90009 2.01660

B 5.99711 -1.69964 0.72549

C 5.40372 -3.06415 1.04433

H 5.58570 -4.86648 1.97154

C 4.94437 -0.77248 0.21821

N 4.10567 -3.32495 0.86438

C 3.34677 -2.36549 0.35496

N 3.66184 -1.12958 0.02889

N -3.12886 1.90815 0.16742

C -3.92601 0.81380 0.15415

N -3.03184 4.23372 0.41351

C -3.68348 3.05901 0.38646

N -5.25391 0.82912 0.35890

C -5.79806 1.99192 0.58636

N -5.05490 3.14517 0.60719

H -3.56457 5.09159 0.51868

N -7.11651 2.08088 0.80814

H -7.64138 1.21281 0.79694

H -7.59552 2.94860 1.00895

N 1.40067 3.00174 -0.09496

N 0.84378 5.27726 -0.09443

C 0.49810 3.99808 -0.01867

H 1.02937 2.06813 0.05170

N 0.16736 7.46108 -0.05283

C -0.15063 6.15469 0.01114

H 1.13954 7.71679 -0.16552

N -0.78463 3.56565 0.15601

C -1.65345 4.53134 0.23269

N -1.45195 5.84045 0.17661

H -0.55280 8.16351 0.03515

N 5.30821 0.56513 -0.13639

H 6.30970 0.69547 -0.22413

N 5.40007 2.78376 -0.62402

C 4.63908 1.70918 -0.28930

N 5.47063 5.03152 -1.08542

C 4.75007 3.92243 -0.76852

H 6.43929 4.91244 -1.34776

N 3.30403 1.78721 -0.10523

C 2.79893 2.99031 -0.28533

N 3.41987 4.10443 -0.62129

H 4.96777 5.86583 -1.35173

H 7.17439 -1.39449 0.88672

H -5.49142 4.04079 0.79527

1BH,12H

C -1.47066 4.51211 -0.59384

N -1.08096 5.74730 -0.94459

C 0.23119 5.92998 -1.03860

C 0.64833 3.83758 -0.44416

N -0.62129 3.50964 -0.32632

H 0.00630 7.86876 -1.63881

N 1.17014 4.98763 -0.80230

N -2.80779 4.33761 -0.50038

N 0.67613 7.15065 -1.39905

N 1.50015 2.73676 -0.16482

H 1.66658 7.29685 -1.53230

H 0.97317 1.86806 -0.11134

H -3.35623 5.17965 -0.63844

N -6.96010 2.66652 0.42768

H -7.55334 1.88001 0.65275

N -5.21791 1.18877 0.32881

C -5.63919 2.44511 0.23833

H -7.32109 3.60816 0.38936

N -3.33412 -0.19737 0.16787

C -3.91173 1.01022 0.12571

N -4.87443 3.52030 -0.02698

C -3.59254 3.20765 -0.20637

N -3.04103 2.02439 -0.15757

H -2.36044 -0.19621 -0.10983

N -6.66267 -3.03673 1.76351

H -7.17102 -3.90214 1.63224

B -4.59876 -4.14862 0.71434

C -5.43147 -2.94165 1.13185

H -7.22190 -2.18927 1.74775

C -3.30378 -3.71119 0.12159

N -5.00293 -1.68902 0.94602

C -3.81959 -1.52330 0.37439

N -2.95453 -2.42301 -0.04290

N 3.37813 1.49118 0.32904

C 2.79750 2.73133 0.17840

N 5.27330 0.21501 0.79669

C 4.69233 1.42664 0.67359

N 3.49155 3.82194 0.38149

C 4.77761 3.67078 0.73070

N 5.42020 2.49714 0.88297

H 6.25934 0.22726 1.04044

N 5.47957 4.78351 0.94880

H 5.02251 5.68165 0.84804

H 6.45065 4.72043 1.22760

N 1.73117 -2.58492 -0.25386

N 3.83111 -3.49961 0.15109

C 3.03419 -2.44752 0.04718

H 1.13581 -1.77731 -0.10478

N 5.92357 -4.30621 0.58817

C 5.09751 -3.25573 0.47282

H 5.55178 -5.23553 0.43812

N 3.45675 -1.16155 0.25758

C 4.73953 -1.06234 0.57071

N 5.62023 -2.03041 0.69778

H 6.88818 -4.16465 0.85461

N -2.37540 -4.68620 -0.36433

H -2.78796 -5.59765 -0.52803

N -0.54275 -5.82449 -1.08803

C -1.06126 -4.67592 -0.58317

N 1.32327 -6.95035 -1.81038

C 0.75765 -5.82064 -1.31011

H 0.71266 -7.68825 -2.13311

N -0.29864 -3.59557 -0.29233

C 0.97800 -3.75049 -0.55654

N 1.59937 -4.78861 -1.07909

H 2.28172 -6.90602 -2.12577

H -4.94905 -5.31627 0.84591

H 2.92365 0.55978 0.17710

3BH,10H

C -4.86973 1.56508 -0.00570

N -5.69647 2.53042 -0.39352

C -5.09489 3.73995 -0.41272

C -3.10250 2.92866 0.25019

N -3.62587 1.66714 0.35844

H -6.83422 4.64944 -1.00655

N -3.82170 3.98947 -0.12748

N -5.47882 0.28488 0.00660

N -5.86493 4.79390 -0.76569

N -1.80213 2.97602 0.55104

H -5.44074 5.71063 -0.80052

H -1.45048 2.05670 0.79258

H -6.48906 0.30665 -0.08609

N -6.07410 -4.24201 0.12216

H -5.71211 -5.18247 0.16984

N -3.91031 -3.43339 0.08247

C -5.24368 -3.18054 0.08896

H -7.07155 -4.07522 0.14979

N -1.77292 -2.44917 0.00201

C -3.17026 -2.32255 0.03107

N -5.78466 -1.97388 0.06491

C -4.92529 -0.93909 0.03098

N -3.58875 -1.08743 -0.00028

H -1.26563 -1.56543 -0.02634

N -1.51884 -7.08688 0.07683

H -0.94745 -7.92372 0.07492

N 0.41346 -5.84634 -0.00027

C -0.89284 -5.90206 0.04046

H -2.52574 -7.16903 0.11189

C 0.97467 -4.61223 -0.03355

N -1.65689 -4.77092 0.04746

C -1.01577 -3.56589 0.00447

N 0.28742 -3.46289 -0.03517

B 0.65011 3.36135 0.53110

C -0.74335 3.92037 0.41116

N 3.06749 4.36023 0.19661

C 1.64706 4.45279 0.25512

N -1.05165 5.18772 0.16135

C -0.02307 6.02079 0.00909

N 1.27063 5.72223 0.02503

H 3.53540 5.25924 0.13032

N -0.34782 7.37296 -0.16935

H -1.28201 7.50199 -0.54306

H 0.37077 7.89208 -0.66230

N 3.52987 -0.16909 0.00047

N 5.50852 1.14515 -0.04192

C 4.19321 1.06933 0.01196

H 2.52290 -0.04674 0.05904

N 7.29269 2.58845 -0.10630

C 5.94802 2.42399 -0.02701

H 7.88434 1.77159 -0.06344

N 3.32875 2.06153 0.08252

C 3.86681 3.28999 0.11082

N 5.19878 3.51396 0.04920

H 7.67621 3.51694 -0.00123

N 2.31550 -4.62402 -0.07140

H 2.73334 -5.54993 -0.08614

N 4.51646 -4.07172 -0.17451

C 3.28170 -3.59172 -0.09629

N 6.74055 -3.49493 -0.27508

C 5.45394 -3.10559 -0.19488

H 6.96784 -4.47792 -0.32101

N 2.90543 -2.34337 -0.03757

C 3.93581 -1.45502 -0.06344

N 5.21496 -1.79455 -0.14211

H 7.46178 -2.78648 -0.29558

H -2.70387 -4.70406 0.07820

H 0.83821 2.18717 0.80092

2BH,10H

C -1.30574 4.71882 -0.02381

N -2.36792 5.54722 -0.02841

C -3.55818 4.97115 -0.02108

C -2.63671 2.92995 -0.00742

N -1.41285 3.38008 -0.01400

H -4.50868 6.77208 -0.03497

N -3.77286 3.62971 -0.00953

N -0.10051 5.30354 -0.03017

N -4.64449 5.76968 -0.02499

N -2.70949 1.53238 0.00262

H -5.57472 5.37933 -0.02241

H -1.80529 1.06240 0.00247

H -0.10804 6.31850 -0.03986

N 4.38438 6.17431 -0.04073

H 5.33859 5.84080 -0.06190

N 3.72907 3.98491 -0.00927

C 3.38492 5.26309 -0.02944

H 4.16631 7.15845 -0.08642

N 2.94399 1.79327 0.02187

C 2.72027 3.10579 0.00280

N 2.12393 5.74110 -0.03713

C 1.21455 4.76924 -0.02330

N 1.39968 3.47974 -0.00451

H 2.10737 1.21764 0.01708

N 7.89607 0.45735 0.07354

H 8.43146 -0.36141 0.34504

N 6.09773 -0.95693 0.04759

C 6.51903 0.30478 0.07191

H 8.24904 1.34618 0.40453

C 4.77450 -1.16933 0.00972

B 5.55973 1.49788 0.07283

C 4.14596 1.01996 0.03132

N 3.80323 -0.30014 -0.00052

N -3.60611 -0.56457 0.01852

C -3.78693 0.72787 0.01154

N -4.60212 -2.66160 0.03144

C -4.70458 -1.32420 0.02663

N -5.02881 1.29561 0.01309

C -6.10965 0.45812 0.02397

N -5.97213 -0.84434 0.03078

H -5.48055 -3.17135 0.03889

N -7.33169 1.00811 0.02731

H -7.47828 2.00842 0.02352

H -8.13058 0.38470 0.03537

N -0.03163 -3.32405 -0.00960

N -1.44617 -5.20235 0.00904

C -1.26214 -3.88978 0.00325

H -0.03064 -2.30938 -0.00141

N -2.94826 -6.92787 0.03178

C -2.71900 -5.60106 0.02399

H -2.15674 -7.55711 0.03321

N -2.26941 -2.97911 0.01130

C -3.46553 -3.50419 0.02440

N -3.79322 -4.78904 0.03164

H -3.89631 -7.27573 0.04954

N 4.52480 -2.57412 -0.02082

H 5.40303 -3.08159 -0.02971

N 3.76091 -4.70050 -0.06346

C 3.45611 -3.37947 -0.03130

N 2.98048 -6.85808 -0.08982

C 2.72960 -5.52448 -0.07297

H 3.93417 -7.17243 -0.19911

N 2.19434 -2.91277 -0.01140

C 1.26663 -3.85782 -0.02689

N 1.42969 -5.16527 -0.05860

H 2.20569 -7.49522 -0.20295

H 5.97242 2.64005 0.11634

H -5.02999 2.34166 0.00574

2BH,12H

C 1.51575 -4.54447 0.54707

N 2.55668 -5.31096 0.91024

C 3.71793 -4.68034 1.03691

C 2.82357 -2.74283 0.44669

N 1.61043 -3.22957 0.29696

H 4.67530 -6.38054 1.63903

N 3.91908 -3.36149 0.82138

N 0.34022 -5.19470 0.41951

N 4.79360 -5.40287 1.41104

N 2.86163 -1.34752 0.18239

H 5.67310 -4.93329 1.57229

H 1.92252 -0.96137 0.11568

H 0.39572 -6.20036 0.53972

N -3.95804 -6.32354 -0.64819

H -4.90327 -6.04529 -0.87340

N -3.45842 -4.10039 -0.45741

C -3.04270 -5.35857 -0.39989

H -3.68186 -7.29381 -0.62603

N -2.82448 -1.87460 -0.19348

C -2.53039 -3.17484 -0.19501

N -1.79092 -5.76464 -0.11682

C -0.96003 -4.74771 0.11121

N -1.22711 -3.47003 0.10084

H -2.05505 -1.27392 0.08428

N -7.68567 -0.65585 -1.30550

H -8.34390 0.05757 -1.00820

N -6.08137 0.73977 -0.45660

C -6.38010 -0.49250 -0.87047

H -8.03914 -1.60396 -1.32073

C -4.82298 0.96309 -0.06547

B -5.35920 -1.63493 -0.88616

C -4.04178 -1.15366 -0.37453

N -3.82230 0.13228 0.00982

N 3.64140 0.77035 -0.29342

C 3.90725 -0.57341 -0.14224

N 4.41416 2.92234 -0.75451

C 4.66477 1.60210 -0.62496

N 5.11672 -1.03810 -0.32832

C 6.06649 -0.15283 -0.66202

N 5.88858 1.17363 -0.81736

H 5.21784 3.49646 -0.99185

N 7.29568 -0.63025 -0.86088

H 7.46013 -1.62432 -0.75789

H 8.04346 -0.00242 -1.12792

N -0.10927 3.08031 0.24886

N 1.03963 5.05896 -0.17404

C 1.02418 3.74138 -0.05242

H -0.10348 2.07367 0.12298

N 2.24744 6.94570 -0.62433

C 2.20700 5.61179 -0.49060

H 1.39419 7.47379 -0.49257

N 2.12880 2.95553 -0.24171

C 3.22371 3.63377 -0.55071

N 3.35756 4.93452 -0.69222

H 3.11017 7.40187 -0.88741

N -4.65688 2.31553 0.36393

H -5.54742 2.78430 0.48879

N -3.87334 4.34661 1.01003

C -3.59544 3.09959 0.56029

N -3.05563 6.38969 1.66196

C -2.82952 5.13053 1.20963

H -3.99149 6.63735 1.95189

N -2.34023 2.67287 0.30173

C -1.40859 3.57305 0.52968

N -1.53773 4.79741 0.99772

H -2.26438 6.95139 1.94153

H -5.65367 -2.75160 -1.26747

H 2.72100 1.24731 -0.15247

2BH,11H

C -2.90524 -3.93764 -0.33881

N -4.15554 -4.41258 -0.47623

C -5.11887 -3.50106 -0.47414

C -3.66845 -1.84633 -0.21505

N -2.61413 -2.63583 -0.20720

H -6.56933 -4.91203 -0.75515

N -4.93663 -2.16865 -0.34406

N -1.93800 -4.87672 -0.33611

N -6.39244 -3.92745 -0.61071

N -3.29422 -0.48714 -0.08712

H -7.13892 -3.24996 -0.66392

H -2.28397 -0.35749 -0.12879

H -2.27854 -5.82895 -0.41697

N 1.94382 -7.24670 0.01327

H 2.94003 -7.26584 0.18428

N 2.08471 -4.96569 0.08426

C 1.32233 -6.04368 -0.01623

H 1.39262 -8.09203 0.01443

N 2.11344 -2.64341 0.12927

C 1.44376 -3.79296 0.03664

N -0.01588 -6.05147 -0.15121

C -0.53619 -4.82367 -0.18954

N 0.08606 -3.67839 -0.11273

H 1.54739 -1.80921 0.00993

N 7.14191 -3.16695 1.01863

H 7.98498 -2.67487 0.73912

N 6.05334 -1.24891 0.40293

C 5.94712 -2.54687 0.68285

H 7.16682 -4.17325 0.91291

C 4.92516 -0.58763 0.11749

B 4.61101 -3.29282 0.65967

C 3.50617 -2.36131 0.28008

N 3.70449 -1.03751 0.03416

N -3.25930 1.75693 0.13932

C -4.00243 0.62484 0.11349

N -3.27768 4.08539 0.36906

C -3.87389 2.88084 0.33197

N -5.33477 0.57919 0.28326

C -5.93964 1.71628 0.48375

N -5.25329 2.90449 0.51241

H -3.84990 4.91744 0.47328

N -7.26653 1.74409 0.66886

H -7.74874 0.85167 0.65239

H -7.79131 2.58918 0.85075

N 1.20658 3.04420 -0.10243

N 0.55956 5.29601 -0.04440

C 0.26321 4.00343 -0.01099

H 0.86858 2.09224 0.00143

N -0.20452 7.44945 0.04728

C -0.47116 6.13045 0.06994

H 0.75820 7.74672 -0.04168

N -1.00219 3.51562 0.12887

C -1.91109 4.44302 0.22114

N -1.76047 5.76016 0.20582

H -0.95334 8.12011 0.14422

N 5.21941 0.78371 -0.15311

H 6.22079 0.93647 -0.20132

N 5.22364 3.02164 -0.51760

C 4.49957 1.90294 -0.26767

N 5.20760 5.29046 -0.86141

C 4.52852 4.13912 -0.62258

H 6.19445 5.23515 -1.07062

N 3.15974 1.90726 -0.13061

C 2.60570 3.09867 -0.25519

N 3.18889 4.25423 -0.50500

H 4.68109 6.12709 -1.06657

H 4.54158 -4.47729 0.92588

H -5.73797 3.78045 0.67304

3BH,11H

C 4.51834 2.29785 0.04070

N 5.18618 3.39392 0.39726

C 4.39639 4.48375 0.45520

C 2.54560 3.35791 -0.14542

N 3.25455 2.19361 -0.25920

H 5.98632 5.67294 0.96840

N 3.09050 4.52321 0.21119

N 5.33932 1.15590 -0.02487

N 4.99109 5.64872 0.80340

N 1.24693 3.20515 -0.42957

H 4.42906 6.48827 0.82549

H 1.03632 2.24411 -0.67220

H 6.33001 1.36355 0.03772

N 6.85886 -3.12062 -0.51129

H 6.66983 -4.10018 -0.66596

N 4.58920 -2.80418 -0.33412

C 5.82561 -2.26363 -0.36494

H 7.79439 -2.74995 -0.60706

N 2.28637 -2.30600 -0.10035

C 3.64586 -1.90217 -0.16896

N 6.10347 -0.97018 -0.26219

C 5.04184 -0.15937 -0.12609

N 3.77422 -0.59524 -0.06444

H 1.64019 -1.51737 -0.10243

N 2.59735 -6.88687 0.31616

H 2.18339 -7.80339 0.42231

N 0.51908 -5.84087 0.21726

C 1.88630 -5.75549 0.21360

H 3.60738 -6.79275 0.31558

C -0.22645 -4.66689 0.13635

N 2.49686 -4.60970 0.11360

C 1.72636 -3.51347 0.01498

N 0.36878 -3.51777 0.03660

B -1.23125 3.25475 -0.52249

C 0.06743 3.99309 -0.31951

N -3.77217 3.88120 -0.24075

C -2.37764 4.18376 -0.24796

N 0.18198 5.28068 -0.01948

C -0.95905 5.95959 0.10815

N -2.19604 5.48651 0.03340

H -4.37544 4.69692 -0.19423

N -0.82934 7.33821 0.33221

H 0.06406 7.57903 0.74830

H -1.62887 7.73800 0.81179

N -3.45912 -0.65025 -0.01495

N -5.64217 0.30034 -0.06737

C -4.33272 0.45556 -0.06118

H -2.48788 -0.34972 -0.03776

N -7.64917 1.41688 -0.07870

C -6.29354 1.48631 -0.10642

H -8.08841 0.51106 -0.15433

N -3.65119 1.57815 -0.09851

C -4.38849 2.69833 -0.16780

N -5.74111 2.68874 -0.16106

H -8.18013 2.26506 -0.21724

N -1.55582 -4.86789 0.17022

H -1.89613 -5.82249 0.23540

N -3.80543 -4.65747 0.17863

C -2.66455 -3.98065 0.13347

N -6.09744 -4.44117 0.19389

C -4.88716 -3.85362 0.15207

H -6.17166 -5.44697 0.24514

N -2.47959 -2.69517 0.06984

C -3.64401 -1.98026 0.04850

N -4.85346 -2.52479 0.08797

H -6.92092 -3.85403 0.17803

H -1.24076 2.07930 -0.84604

H 0.05595 -6.73986 0.28955

3BH,12H

C 5.05266 0.23148 0.01774

N 6.10893 0.97822 0.33513

C 5.88741 2.29354 0.15054

C 3.77230 1.98823 -0.54304

N 3.90530 0.63226 -0.45340

H 7.78647 2.75622 0.76830

N 4.74904 2.84926 -0.25667

N 5.25966 -1.13980 0.25337

N 6.91911 3.12876 0.41120

N 2.54534 2.36496 -0.93362

H 6.76721 4.12396 0.32276

H 1.95301 1.55465 -1.07178

H 6.20863 -1.36363 0.53313

N 4.58172 -5.53611 1.27843

H 3.94714 -6.29817 1.46994

N 2.74890 -4.27493 0.70452

C 4.08351 -4.34287 0.89424

H 5.56654 -5.61244 1.49329

N 0.96534 -2.86247 0.08417

C 2.35353 -3.08573 0.31051

N 4.93306 -3.33467 0.73577

C 4.38507 -2.16736 0.36539

N 3.07399 -2.00010 0.12801

H 0.73463 -1.87180 0.05048

N -0.38251 -7.11714 -1.03458

H -1.11583 -7.78026 -1.25179

N -1.99906 -5.52798 -0.72152

C -0.69452 -5.85481 -0.73819

H 0.59247 -7.39028 -1.05942

C -2.26444 -4.26833 -0.46926

N 0.32119 -5.01988 -0.48408

C 0.02533 -3.77505 -0.21174

N -1.28846 -3.34683 -0.22947

B 0.28177 3.36067 -0.70071

C 1.77591 3.55488 -0.78523

N -1.73819 4.94113 -0.08834

C -0.36662 4.67298 -0.37446

N 2.41047 4.71360 -0.68150

C 1.64537 5.78769 -0.47375

N 0.33289 5.82071 -0.29385

H -1.94765 5.93018 0.01295

N 2.31204 7.01950 -0.45931

H 3.29454 6.92855 -0.22418

H 1.82160 7.73343 0.06828

N -3.27872 0.66693 0.31382

N -4.81854 2.44357 0.68745

C -3.60006 2.04599 0.38773

H -2.28245 0.54227 0.15988

N -6.14944 4.28882 1.00316

C -4.92120 3.79429 0.70897

H -6.87349 3.64948 1.29657

N -2.54185 2.78095 0.13630

C -2.74839 4.11071 0.17158

N -3.94993 4.65877 0.46539

H -6.25493 5.28607 1.12574

N -3.55913 -3.89458 -0.43426

H -4.22290 -4.64566 -0.60037

N -5.46450 -2.68921 -0.18166

C -4.14840 -2.64690 -0.18727

N -7.37300 -1.43941 0.09749

C -6.03293 -1.48797 0.05191

H -7.91511 -2.27821 -0.05729

N -3.36216 -1.60074 -0.00184

C -4.04736 -0.42815 0.19013

N -5.36807 -0.35114 0.23596

H -7.82188 -0.54540 0.24999

H -0.21647 2.25588 -0.84730

H -1.62735 -2.38098 -0.04289
